# The efficacy and safety of Fuzheng traditional Chinese medicine injections in the treatment of acute ischemic stroke: a systematic review and network meta-analysis

**DOI:** 10.3389/fphar.2025.1658367

**Published:** 2025-11-14

**Authors:** Qiaosheng Ren, Linlin Guo, Peichi Zhang, Xuefeng Wu, Yingzhi Xu, Kegang Cao

**Affiliations:** Dongzhimen Hospital, Beijing University of Chinese Medicine, Beijing, China

**Keywords:** acute ischemic stroke, traditional Chinese medicine injections, network meta-analysis, randomized controlled trials, NIHSS

## Abstract

**Objective:**

The primary objective of this network meta-analysis (NMA) was to compare and rank the relative efficacy and safety of seven distinct Fuzheng traditional Chinese medicine injections (TCMIs) combined with conventional treatment (CT), against CT, for the management of acute ischemic stroke (AIS). The overarching hypothesis was that although TCMIs + CT as a class provides benefit over CT alone, their therapeutic profiles differ.

**Methods:**

The Cochrane Library, Embase, PubMed, Web of science, CNKI, Wanfang, VIP, and SinoMed were comprehensively searched from their inception to 11 January 2025, for randomized controlled trials (RCTs) focusing on the use of Fuzheng TCMIs + CT to treat AIS. The quality of the included RCTs was assessed using the risk-of-bias 2 (RoB2) tool. NMA in the frequentist framework was designed to access the efficacy of different Fuzheng TCMIs.

**Results:**

A total of 70 RCTs involving 6,227 patients were enrolled. The study showed that Mailuoning injection (MLN), Huangqi injection (HQ), Shengmai injection (SHM), Ciwujia injection (CWJ), Shenfu injection (SF), and Shenmai injection (SM) combined with CT significantly improved performance compared with CT alone in treating AIS. In terms of the increase in the clinical efficacy rate, MLN + CT was most likely to be the best course of action, as was CWJ + CT in terms of the National Institute of Health Stroke Scale Score (NIHSS), Barthel Index (BI), blood lipids, the low-cut viscosity of whole blood (LCV), and interleukin-6 (IL-6). CWJ + CT was associated with the lowest rate of adverse reactions (ADRs) although the evidence base for safety comparisons remains limited. Additionally, MLN + CT was most likely to be the best treatment in terms of plasma viscosity (PV), fibrinogen (FIB) and modified Rankin Scale (mRS). Given that SF + CT ameliorated the activities of daily living (ADL) rating and C-reactive protein (CRP) level, it was most likely to be the best course of action. The treatment that was most likely to be the best in terms of the high-cut viscosity of whole blood (HCV) was SHM + CT. Considering the decrease in the tumor necrosis factor-α (TNF-α) level, SM + CT had the best chance of being the best treatment.

**Conclusion:**

The combination of CT and TCMIs had a more beneficial impact on the treatment of AIS. Both CWJ + CT and MLN + CT performed best. However, these findings should be interpreted with caution due to the methodological limitations of the included trials. Therefore, to substantiate the findings, more excellent research is required.

**Systematic Review Registration:**

CRD42025614168.

## 1 Introduction

As one of the main causes of death in China and worldwide, stroke is characterized by elevated rates of death and disability, which can be mainly classified into ischemic and hemorrhagic types (Ma et al., 2021; Yin et al., 2025). In China, stroke is the most common cause of death, and its prevalence is increasing at a rate of 8.7%, with approximately 2 million new cases occurring there each year. This rate is higher than the global average (Wang et al., 2017; Ma et al., 2024). Approximately 85% of all stroke cases are brought on by vascular occlusion, which causes a brain infarction and is more precisely referred to as acute ischemic stroke (AIS) (Bogenschutz et al., 2025). Reperfusion treatments, such as intravenous thrombolysis and endovascular thrombectomy (EVT), can restore cerebral blood flow and greatly enhance functional outcomes in the early stages of ischemic stroke (Xiong et al., 2025, p. 202; Zhou et al., 2025). In addition, there are other treatments, such as antiplatelet aggregation (Lim et al., 2024), anticoagulation (Lv et al., 2025), reducing the fibrinogen levels (Barakzie et al., 2025) and blood lipids (Chen et al., 2025), and neuroprotection (Wang et al., 2024). The internationally accepted cornerstone of treating AIS is administered intravenously with alteplase 4.5 h after the onset of symptoms (Sun et al., 2024). However, patients receiving intravenous thrombolysis are at risk of experiencing symptoms of intracerebral hemorrhage, and China has a relatively small population of patients who receive thrombolysis because of its stringent treatment window (Nisar et al., 2019; Zhu et al., 2020). Therefore, it is particularly important to find a safe and efficient adjuvant medication treatment for AIS in clinical practice.

Since ancient times, traditional Chinese medicine (TCM) has been applied to numerous medical conditions and is proven to have good therapeutic efficacy and safety (Xiaoqiang et al., 2022; Liu et al., 2024; Ning et al., 2024). According to the theories of TCM, AIS is mainly due to the deficiency of “zheng qi”; therefore, an approach of “fu zheng” combined with conventional treatments (CTs) allows the treatment of AIS to be broadened (Zhou et al., 2020). TCMIs are made by using contemporary scientific procedures and techniques to extract and purify potent compounds from herbs (Liu et al., 2018). Because of their immediate efficacy and high bioavailability, TCMIs are frequently used in conjunction with conventional treatments to treat acute illnesses in China (Li et al., 2022). Nowadays, a variety of Fuzheng TCMIs are widely used in treating acute ischemic stroke patients, including Mailuoning injection (MLN), Huangqi injection (HQ), Shengmai injection (SHM), Ciwujia injection (CWJ), Shenfu injection (SF), Shenmai injection (SM), and Shenqifuzheng injection (SQFZ). The Chinese State Pharmaceutical Administration has authorized each injection although there are differences in neurologic recovery, quality of life, lipids, blood rheology, immunity, and adverse effects with each injection, so independent assessment is needed. Clinicians must be aware of the benefits and drawbacks of each Fuzheng TCMIs to make appropriate clinical decisions.

One technique for comparing multiple interventions simultaneously is network meta-analysis (NMA), which combines direct and indirect evidence to estimate and rank their relative efficacy and safety. The efficacy of multiple TCMIs has been compared in prior network meta-analyses, but these are no longer enough to offer timely and sufficient support, and an updated synthesis of the evidence in conjunction with several recently published clinical trials is needed (Li et al., 2022; Xu et al., 2023). Consequently, we conducted this NMA to comprehensively evaluate and compare the efficacy and safety profiles of these seven Fuzheng TCMIs—CWJ, HQ, MLN, SF, SHM, SM, and SQFZ—when used as adjunctive therapy with CT in patients with AIS, thereby providing evidence-based guidance for clinical decision-making.

Therefore, the primary hypothesis of this NMA was that the seven included Fuzheng TCMIs differ in their efficacy and safety profiles when added to CT. The goal is to rank these interventions across multiple outcomes to inform clinical decision-making, rather than solely to test the consistent benefit of TCMIs as a group over CT alone, which is established by the pairwise comparisons within the network. This approach allows for the identification of the most promising TCMI options for specific therapeutic goals in AIS management.

## 2 Data and methods

This systematic review and meta-analysis was carried out in accordance with the Preferred Reporting Items for Systematic Reviews and Meta-Analyses (PRISMA) Statement. PROSPERO had the study protocol registered (registration number: CRD42025614168).

The PRISMA 2020 and the PRISMA-NMA extension statement for network meta-analyses were closely adhered to in this study (Page et al., 2021), as shown in Supplementary File 1.

### 2.1 Literature screening

Studies were retrieved from the Cochrane Library, PubMed, Embase.com, and Web of Science databases using a combination of MeSH and free-text terms starting from the establishment of each database to 31 December 2024. However, due to the limited number of relevant studies identified from these databases, additional searches were carried out in Chinese databases, such as CNKI, WanFang, VIP, and SinoMed, starting from the establishment of each database to 11 January 2025, to ensure a more comprehensive coverage of literature, particularly from regions where TCM interventions are more commonly studied and reported. In addition, to minimize the risk of publication bias, we also searched for the gray literature and ongoing trials. In particular, we conducted a systematic search of clinical trial registries, including ClinicalTrials.gov (www.clinicaltrials.gov), ITMCTR (http://itmctr.ccebtcm.org.cn/), and the WHO International Clinical Trials Registry Platform (ICTRP) (https://www.who.int/tools/clinical-trials-registry-platform/the-ictrp-search-portal). Supplementary File 1 presents the specific search strategy.

### 2.2 Inclusion and exclusion criteria

The inclusion criteria met the PICOS criteria as follows.

1. Participants (P): included patients diagnosed with AIS who had clear diagnostic criteria and had been diagnosed within 2 weeks of onset.
2. Interventions and comparisons (I/C): patients in the control group who received CTs, including antiplatelet, anticoagulant, cerebral circulation enhancement, nerve nourishment, Defibrase, statins, and radical scavenging, whereas the experimental group received one of the following seven kinds of Fuzheng TCMIs: CWJ, HQ, MLN, SF, SHM, SM, and SQFZ. The TCMIs selected by NMA are sourced from the China Medical Information Platform.
3. Outcomes (O): the primary outcome indicators for this analysis were the clinical effective rate and the National Institute of Health Stroke Scale Score (NIHSS), and the former was judged according to the NIHSS evaluation: “basic cure,” “notable progress,” and “progress” are associated with reductions of 91%–100%, 46%–90%, and 18%–45%, respectively. Secondary outcomes (core functional outcomes) included the scores of the Barthel Index (BI), the activities of daily living (ADL), and modified Rankin Scale (mRS). Exploratory outcomes (laboratory biomarkers): to investigate potential mechanisms, we also extracted data on laboratory biomarkers, including lipid profiles [total cholesterol (TC), triglycerides (TGs), low-density lipoprotein cholesterol (LDL-C), and high-density lipoprotein cholesterol (HDL-C)], hemorheology indices [the high-cut viscosity of whole blood (HCV), the low-cut viscosity of whole blood (LCV), plasma viscosity (PV), and fibrinogen (FIB)], and hemorheology indices [C-reactive protein (CRP) level, interleukin-6 (IL-6) level, and tumor necrosis factor-α (TNF-α)]. Additionally, the safety outcome was the rate of adverse reactions (ADRs).
4. Study design (S): randomized controlled trials.

Exclusions applied to studies that were non-RCT, not available in full text, non-original articles such as animal experiments, case reports, reviews, and conference papers, and those with duplicate or non-applicable data.

### 2.3 Data extraction

EndNote 20.3 (Clarivate Analytics, London, United Kingdom) was used to manage all of the articles and selected by two independent reviewers (Linlin Guo and Qiaosheng Ren). The reviewers conducted their own independent data extraction, cross-checking, and literature screening. When there were disagreements, they were resolved through discussion or by consulting a third reviewer (Peichi Zhang). After screening the literature by looking at the titles, articles that were blatantly irrelevant were eliminated, and then abstracts and full texts were examined in more detail. To gather crucial information that was unclear but essential for this investigation, efforts were made, if necessary, to contact the original authors of the studies by phone or email. The extracted data included the following: 1) the title, first author, publication year, each group’s sample size, age, and other basic details of the included studies; 2) specifics of the intervention, including the kinds of TCMIs, dosage, and treatment plan; 3) outcome indicators, such as clinical efficacy rate, NIHSS, BI, ADL rating, TC, TG, LDL-C, HDL-C, HCV, LCV, PV, FIB level, CRP level, IL-6 level, TNF-α level, mRS, and ADRs; and 4) key elements of risk-of-bias evaluation.

### 2.4 Quality assessment

The included RCTs were evaluated for methodological quality using the Cochrane risk-of-bias tool (RoB 2), as recommended in the Cochrane Handbook for Systematic Reviews of Interventions, version 6.5 (2024). Any disputes were settled by consensus or by a third research worker, Peichi Zhang. We plotted a “traffic light” plot and a weighted bar plot using the robvis package in R to show the risk-of-bias assessment results (McGuinness and Higgins, 2021). Bias arising from the randomization process, bias due to deviations from intended interventions, bias due to missing outcome data, bias in measurement of the outcome, and bias in selection of the reported result were among the items on the tool assessment.

### 2.5 Statistical analysis

To generate the risk-of-bias diagram for quality evaluation, RevMan 5.4 software was used. Network graphs and the surface under the cumulative ranking area (SUCRA) were created using two packages created with STATA 14 software (College Station, TX, United States): network and mvmeta. Interventions were represented by nodes in the network graphs, and direct comparisons were shown by link lines. SUCRA was used to rank the probability of the intervention for each outcome; a higher area under the curve denoted a better cure (Zhou et al., 2020). Publication bias was assessed using comparison-corrected funnel plots, with *p* < 0.05 considered statistically significant. NMAs were performed with standard mean differences (SMDs) and corresponding 95% confidence intervals (CIs) for continuous outcomes and odds ratios (OR) and corresponding 95% CIs for dichotomous outcomes. We separated multi-arm studies into two-arm trials when possible and assessed heterogeneity with I^2^ (low <75%; high ≥75%) based on techniques that have already been published. To assess the robustness of results, sensitivity analysis was conducted. Additionally, as this NMA lacked closed loops, the premise of consistency between direct and indirect evidence was not applied in this NMA because of the absence of closed loop. Finally, forest maps were drawn to present the results of individual studies.

### 2.6 Data synthesis and analysis

The results were converted from micrograms per liter (μg/L) to nanograms per liter (ng/L), along with the means and standard deviations of the changes. The following formula was used for studies with missing standard deviation data:

$$\text{SD}=\text{ABS}\left(\text{mean}1-\text{mean}2\right)÷\text{TINV}\left(P,\left(n1+n2-2\right)\right)÷\text{SQRT}\left(1/n1+1/n2\right).$$

### 2.7 Data synthesis and analysis

The GRADE method was used to evaluate the evidence’s strength (Alonso-Coello et al., 2018). Strength of evidence was categorized into four grades: high, moderate, low, or very low. Randomized controlled trials had a default initial grade of “high” and were then downgraded according to the following prespecified criteria: risk of bias (bias in randomization, allocation concealment, and blinding of included studies), inconsistency (poor overlap of confidence intervals across studies and I^2^ value of the combined results greater than 75%), indirectness (presence of factors limiting the generalizability of the results), imprecision (small sample sizes and wide confidence intervals for the included studies), and other considerations.

## 3 Results

### 3.1 Literature selection

**FIGURE 1:**
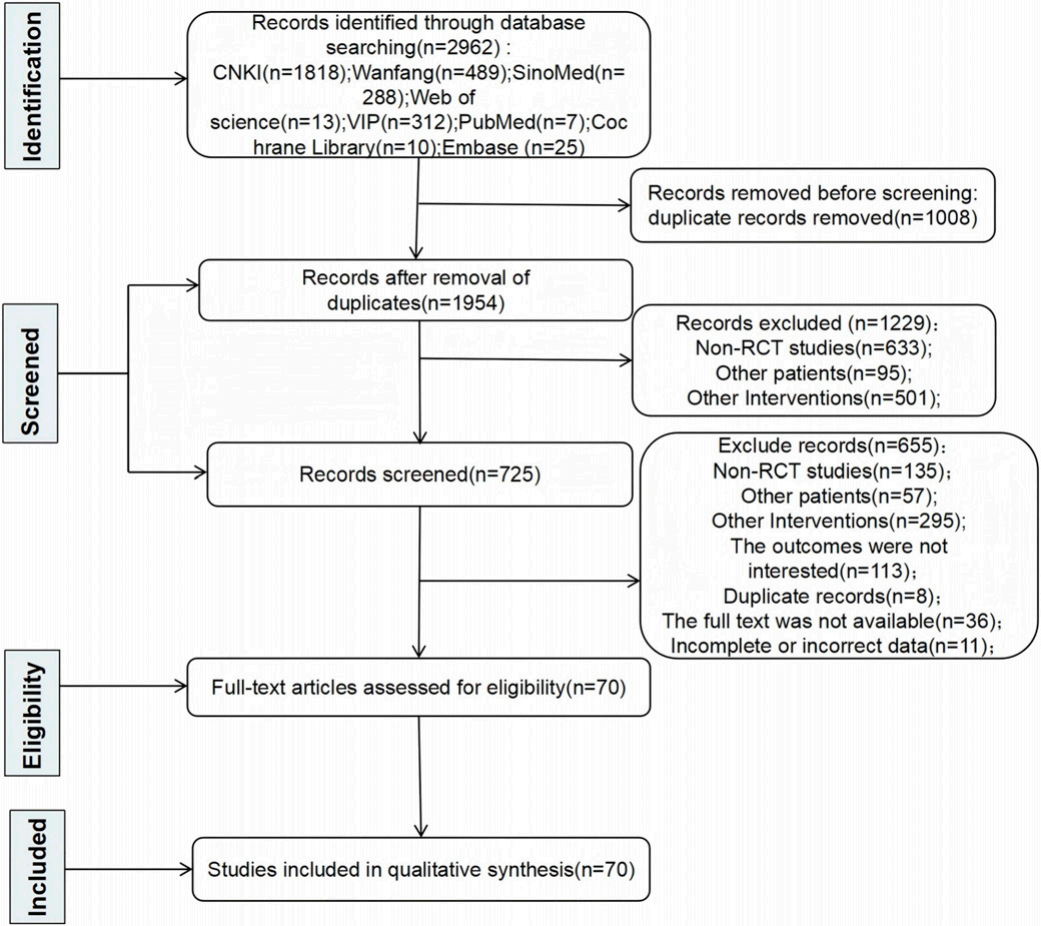
Literature search flow.

A total of 2,962 pertinent articles were found applying the search approach. Duplicate articles were eliminated, yielding 1,954 articles. After that, 1,229 articles were excluded because they were non-RCT studies, involved other patient populations, or were otherwise irrelevant. Seven hundred twenty-five articles were evaluated for eligibility. Then, 655 articles were excluded for the following reasons: non-RCT studies, involvement of other patient populations, other irrelevant studies, outcomes not of interest, duplicate reports, full text not available, or incomplete or incorrect data. In the end, 70 RCTs were included in this NMA after a multitiered selection procedure. The results and selection procedure are shown in Figure 1.

### 3.2 Study characteristics

**FIGURE 2:**
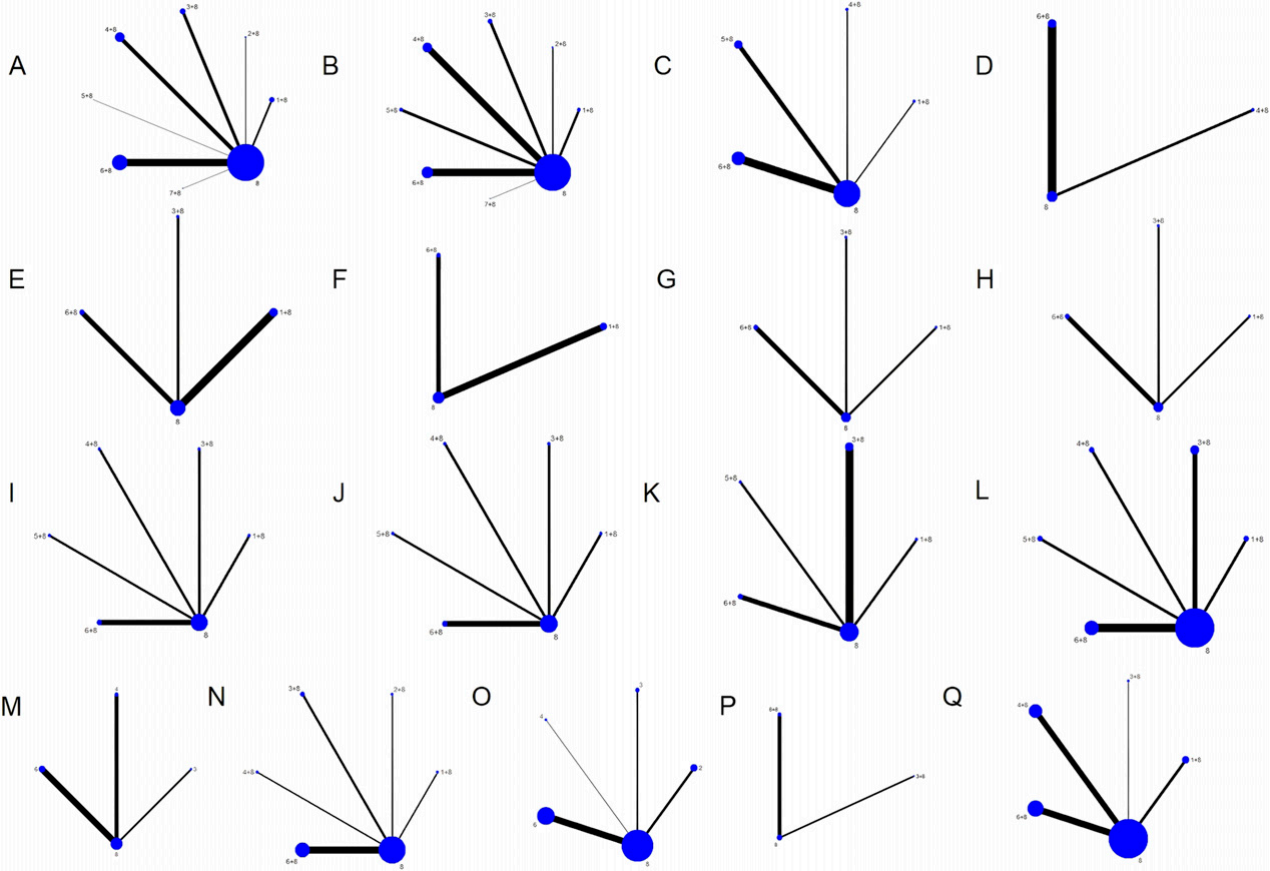
Network diagrams of the outcomes. Note: **(A)** clinical efficacy rate; **(B)** NIHSS; **(C)** BI; **(D)** ADL; **(E)** TC; **(F)** TG; **(G)** LDL-C; **(H)** HDL-C; **(I)** HCV; **(J)** LCV; **(K)** PV; **(L)** FIB; **(M)** CRP; **(N)** IL-6; **(O)** TNF-α; **(P)** mRS; **(Q)** ADRs.

There were seven different kinds of TCMIs included, namely, CWJ, HQ, MLN, SF, SHM, SM, and SQFZ. Overall, the 70 RCTs involved 6,227 patients (3,141 in the test group and 3,086 in the control group). A total of seven comparisons were assessed: CWJ + CT vs. CT (n = 7), HQ + CT vs. CT (n = 5), MLN + CT vs. CT (n = 9), SF + CT vs. CT (n = 16), SHM + CT vs. CT (n = 7), SM + CT vs. CT (n = 25), and SQFZ + CT vs. CT (n = 1). The control groups received CTs, which mostly consisted of antiplatelet, anticoagulation, nerve nutrition, radical scavenging, Defibrase, and statins. Table A.39 (see Supplementary File 1) shows the characteristics of the RCTs, and Figure 2 presents the network graphs of the seven TCMIs with various results.

### 3.3 Bias risk assessment chart

**FIGURE 3:**
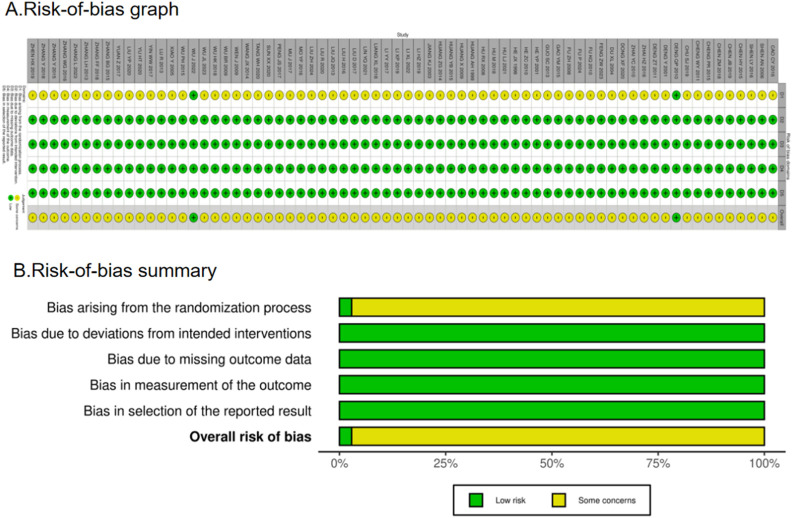
Risk-of-bias summary **(A)** and graph **(B)** are summarized for all included studies.

There were a total of 70 RCTs. Of these, 36 articles clearly stated the method they used to generate a random sequence, whereas 5 articles grouped patients according to their order of hospitalization; the remaining RCTs simply mentioned the term “random.” Only two publications offered thorough explanations of the techniques utilized for sequence allocation that was hidden and were considered “low risk.” The majority of trials using randomized allocation provided outcome data, with the few patients lost to follow-up not considered to have influenced outcome values. Consequently, outcome assessment was not significantly affected, and all trials were rated as “low risk” (100%) in the “missing outcome data” domain. Furthermore, all included studies were rated as “low risk” for outcome measurement as they used appropriate outcome measurement methods and maintained methodological consistency throughout the trials, thereby ensuring objectivity. Nearly all articles reported their results, and no evidence of selective outcome reporting was found. The assessment for “selection of the reported result” was rated as “low risk.” Overall, the trials included in this network meta-analysis were rated as “some concerns.” However, it is crucial to interpret this “some concerns” rating with caution. The predominance of this category is primarily driven by the pervasive lack of reporting on allocation concealment and blinding. In practice, the absence of these critical methodological safeguards introduced a high risk of performance and detection bias. The fact that only 2.9% (2/70) of studies adequately described allocation concealment is a significant limitation. Consequently, although formally categorized as “some concerns” due to insufficient reporting, the actual risk of bias in many included studies is likely substantial, potentially leading to an overestimation of the treatment effects observed in this analysis. Figure 3 shows the bias risk assessment.

### 3.4 Results

The results for the primary and secondary functional outcomes (clinical efficacy rate, NIHSS, BI, ADL, and mRS) are presented first, followed by the results of the exploratory analyses on laboratory biomarkers.

#### 3.4.1 Clinical efficacy rates

**FIGURE 4:**
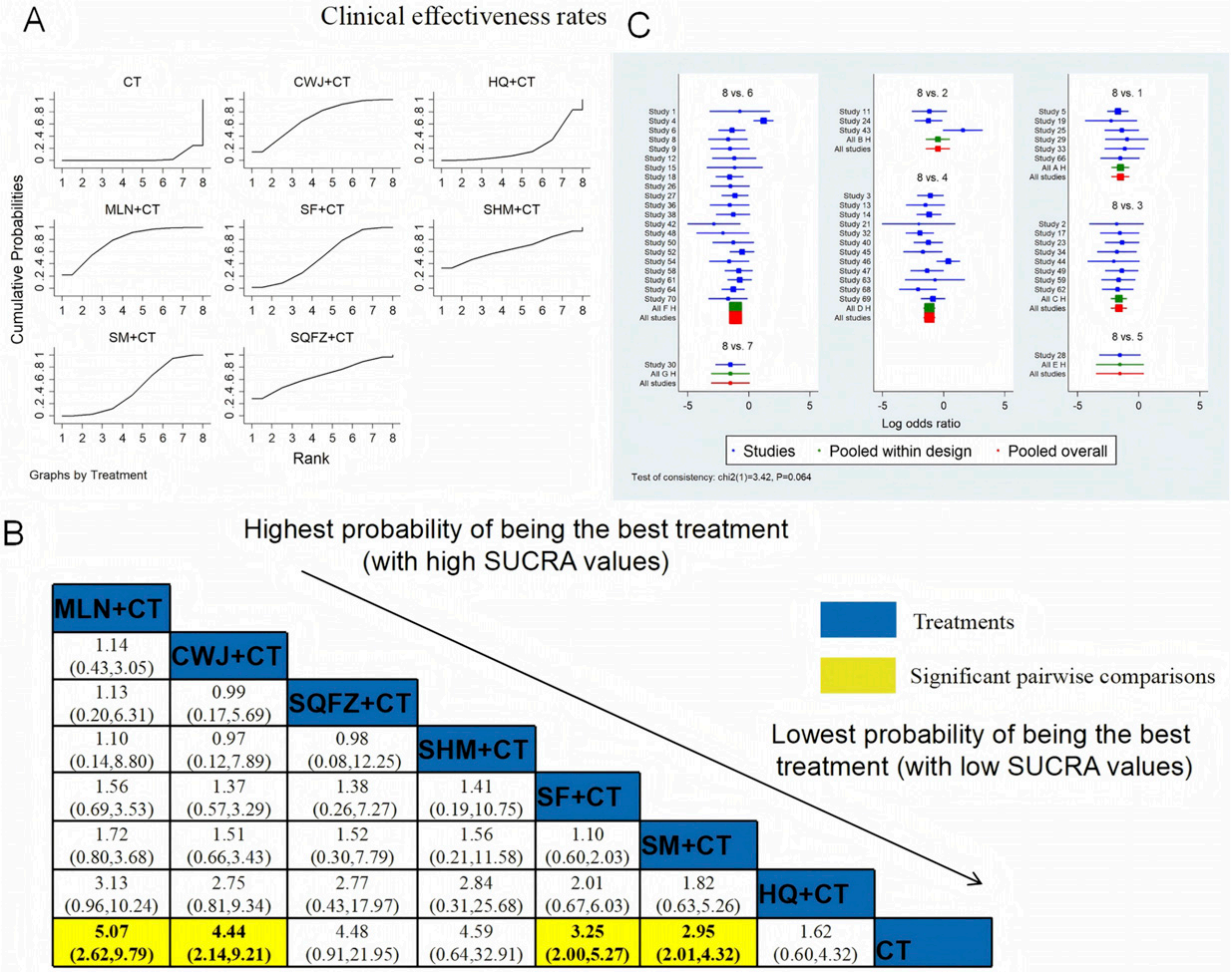
NWA-based relative effect sizes for the clinical efficacy rate. **(A)** Clinical efficacy rate SUCRA graph; **(B)** ORs with clinical 95% CIs efficacy rates; noteworthy, pairwise comparisons are indicated with yellow highlights; **(C)** forest plot of network effect sizes for the clinical efficacy rates.

The clinical efficacy rates were compared across 52 RCTs: CWJ + CT vs. CT (n = 6), HQ injection + CT vs. CT (n = 3), MLN + CT vs. CT (n = 8), SF + CT vs. CT (n = 12), SHM + CT vs. CT (n = 1), SM + CT vs. CT (n = 21), and SQFZ + CT vs. CT (n = 1). Figure 4 shows the comparisons of MLN + CT vs. CT (OR = 5.07; 95% CI: 2.62 and 9.79), CWJ + CT vs. CT (OR = 4.44; 95% CI: 2.14 and 9.21), SF + CT vs. CT (OR = 3.25; 95% CI: 2.00 and 5.27), and SM + CT vs. CT (OR = 2.95; 95% CI: 2.01 and 4.32). The pairwise comparisons of other TCMI types showed no discernible differences. The SUCRA is shown in Table 1: CWJ + CT vs. CT (70.4%), HQ + CT vs. CT (20.8%), MLN + CT vs. CT (77.7%), SF + CT vs. CT (51.7%), SHM + CT vs. CT (64.7%), SM + CT vs. CT (44.6%), and SQFZ + CT (66.3%), and CT (3.9%). Based on SUCRA rankings, MLN + CT had the highest probability of being the most effective intervention for improving the clinical efficacy rate although the pairwise comparisons did not show statistically significant differences between the most active interventions.

**TABLE 1:** SUCRA values (%) of each treatment for outcomes.

Treatment	Outcome																
	Clinical efficacy rate	NIHSS	BI	ADL	mRS	TC	TG	LDL-C	HDL-C	HCV	LCV	PV	FIB	CRP	IL-6	TNF-α	ADRs
CWJ + CT	70.4^*^	**77.5** ^*^	**86.1** ^*^			**85.0** ^*^	**86.6**	**99.9** ^*^	**100.0** ^*^	48.9	**67.7**	26.9	66.6^*^		**76.0**		**85.7**
HQ + CT	20.8	52.3^*^													35.2	40.9	
MLN + CT	**77.7** ^*^	69.9^*^			**99.0** ^*^	36.5		52.6	39.6	47.4	37.6	**93.4** ^*^	**83.5** ^*^	26.2	31.4	46.6	42.3
SF + CT	51.7^*^	71.3^*^	50.3^*^	**86.4**						33.8	47.1		23.7	**81.6** ^*^	70.4	71.4	40.7
SHM + CT	64.7	51.2^*^	52.1^*^							**75.3**	59.1	70.0^*^	43.0				
SM + CT	44.6^*^	41.7^*^	61.3^*^	40.2	51.0^*^	55.1	36.8	34.3	42.7	69.7	64.3	53.1^*^	73.6^*^	76.6^*^	71.7^*^	**73.0** ^*^	49.9
SQFZ + CT	66.3	32.7															
CT	3.9	3.3	0.2	23.4	0	23.4	26.6	13.1	17.7	24.9	24.2	6.6	9.6	15.5	15.3	18.2	31.4

Bold values represent the best-ranked intervention (highest SUCRA value) for each outcome. * indicates that the comparison between this intervention and CT showed a statistically significant difference (p < 0.05).

#### 3.4.2 NIHSS

**FIGURE 5:**
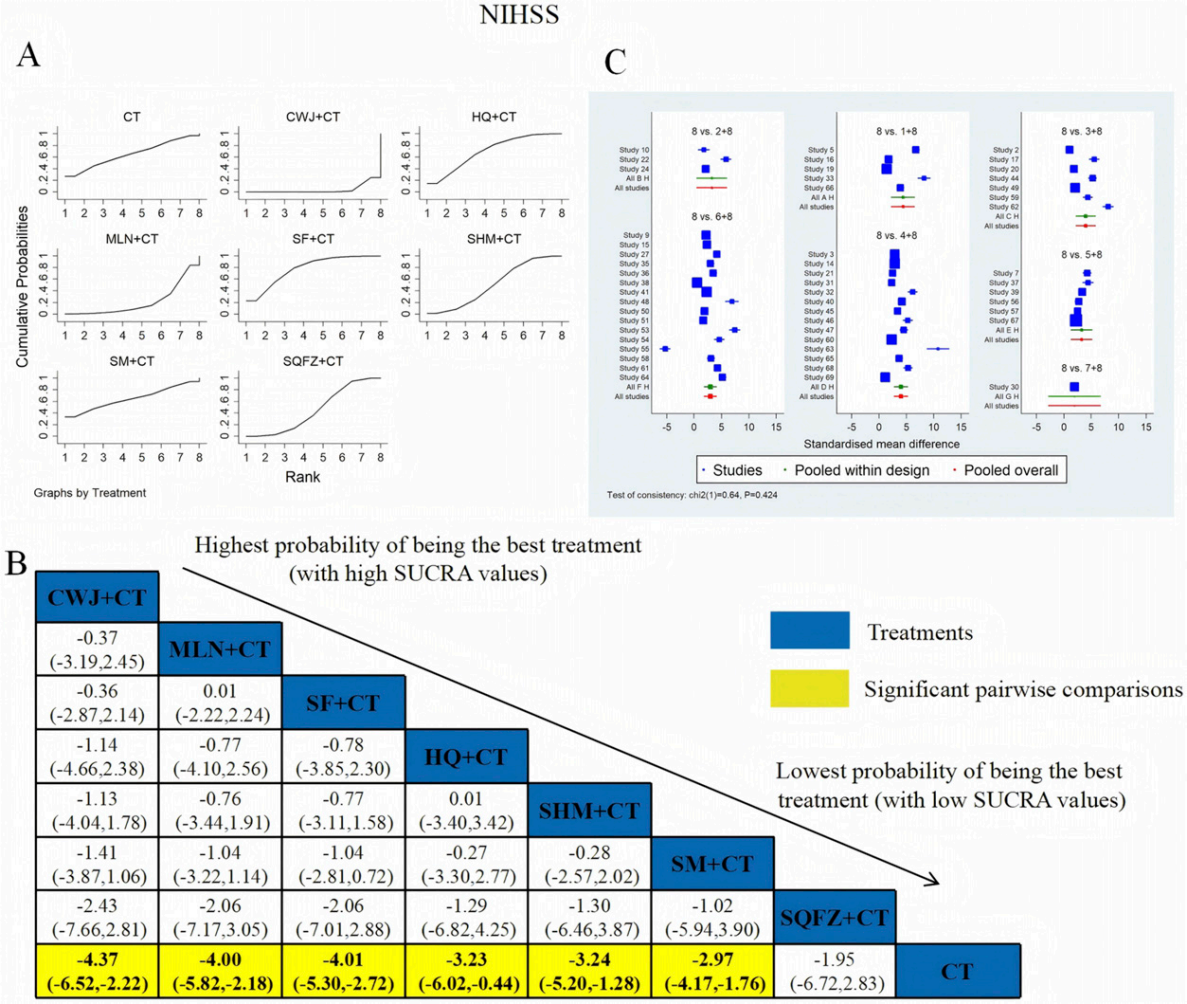
Relative effect sizes of the NIHSS according to NWA. **(A)** SUCRA graph for the NIHSS; **(B)** MDs with 95% CIs of the NIHSS; significant pairwise comparisons are highlighted in yellow; **(C)** forest plot of network effect sizes for the NIHSS.

NIHSS was examined in 52 RCTs: CWJ + CT vs. CT (n = 5), HQ + CT vs. CT (n = 3), MLN + CT vs. CT (n = 7), SF + CT vs. CT (n = 14), SHM + CT vs. CT (n = 6), SM + CT vs. CT (n = 16), and SQFZ + CT vs. CT (n = 1). Figure 5 shows the comparisons of CWJ + CT vs. CT (MD = −4.37; 95% CI: −6.52 and −2.22), MLN + CT vs. CT (MD = −4.00; 95% CI: −5.82 and −2.18), SF + CT vs. CT (MD = −4.01; 95% CI: −5.30 and −2.72), HQ + CT vs. CT (MD = −3.23; 95% CI: −6.02 and −0.44), SHM + CT vs. CT (MD = −3.24; 95% CI: −5.20 and −1.28), and SM + CT vs. CT (MD = −2.97; 95% CI: −4.17 and −1.76). The pairwise comparisons of other TCMI types showed no discernible differences. The SUCRA is shown in Table 1: CWJ + CT vs. CT (77.5%), HQ + CT vs. CT (52.3%), MLN + CT vs. CT (69.9%), SF + CT vs. CT (71.3%), SHM + CT vs. CT (51.2%), SM + CT vs. CT (41.7%), SQFZ + CT (32.7%), and CT (3.3%). Therefore, treatment with CWJ + CT was most likely the highest-ranked intervention for NIHSS improvement.

#### 3.4.3 BI

**FIGURE 6:**
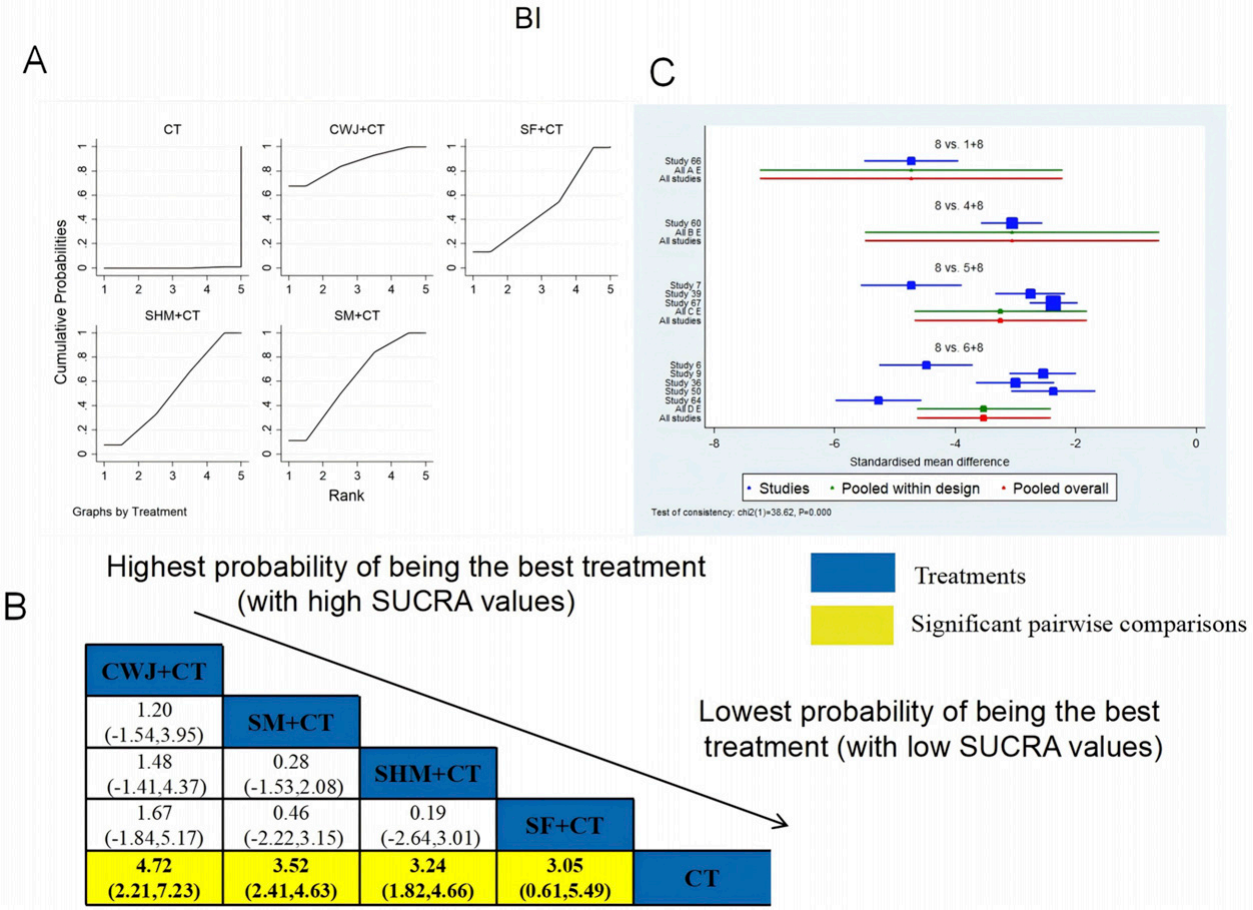
Relative effect sizes of the BI based on NWA. **(A)** The BI’s SUCRA graph; **(B)** MDs with the BI’s 95% CIs; yellow highlights indicate significant pairwise comparisons; **(C)** forest plot of network effect sizes for BI.

BI was examined in 10 RCTs: CWJ + CT vs. CT (n = 1), SM + CT vs. CT (n = 5), SHM + CT vs. CT (n = 3), and SF + CT vs. CT (n = 1). Figure 6 shows the comparisons of CWJ + CT vs. CT (MD = 4.72; 95% CI: 2.21 and 7.23), SM + CT vs. CT (MD = 3.52; 95% CI: 2.41 and 4.63), SHM + CT vs. CT (MD = 3.24; 95% CI: 1.82 and 4.66), and SF + CT vs. CT (MD = 3.05; 95% CI: 0.61 and 5.49). There were no significant differences in the pairwise comparisons of other types of TCMIs. The SUCRA is shown in Table 1: CWJ + CT vs. CT (86.1%), SF + CT vs. CT (50.3%), SHM + CT vs. CT (52.1%), SM + CT vs. CT (61.3%), and CT (0.2%). Therefore, CWJ + CT was most likely the highest-ranked intervention for improving BI.

#### 3.4.4 ADL

**FIGURE 7:**
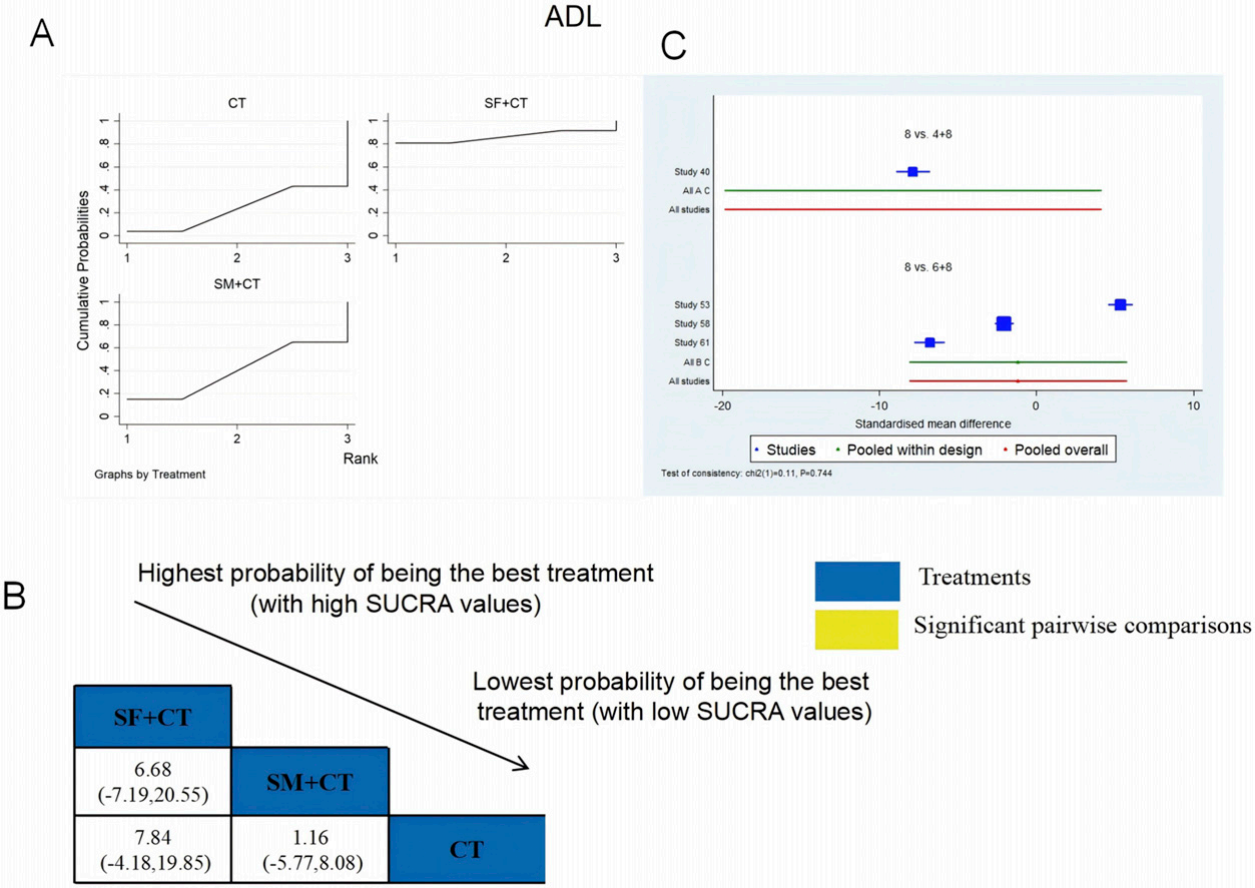
Relative effect sizes of ADL based on NWA. **(A)** ADL’s SUCRA graph; **(B)** MDs with ADL’s 95% CIs; significant pairwise comparisons are highlighted in yellow; **(C)** forest plot of network effect sizes for ADL.

ADL was examined in four RCTs: SF + CT vs. CT (n = 1) and SM + CT vs. CT (n = 3). Figure 7 shows the comparisons of SF + CT vs. CT (MD = 7.84; 95% CI: −4.18 and 19.85) and SM + CT vs. CT (MD = 1.16; 95% CI: −5.77 and 8.08). The TCMI pairwise comparisons showed no discernible differences. The SUCRA is shown in Table 1: SF + CT vs. CT (86.4%), SM + CT vs. CT (40.2%), and CT (23.4%). Therefore, In terms of improving ADL, SF + CT had the highest probability of being the most successful intervention.

#### 3.4.5 mRS

**FIGURE 8:**
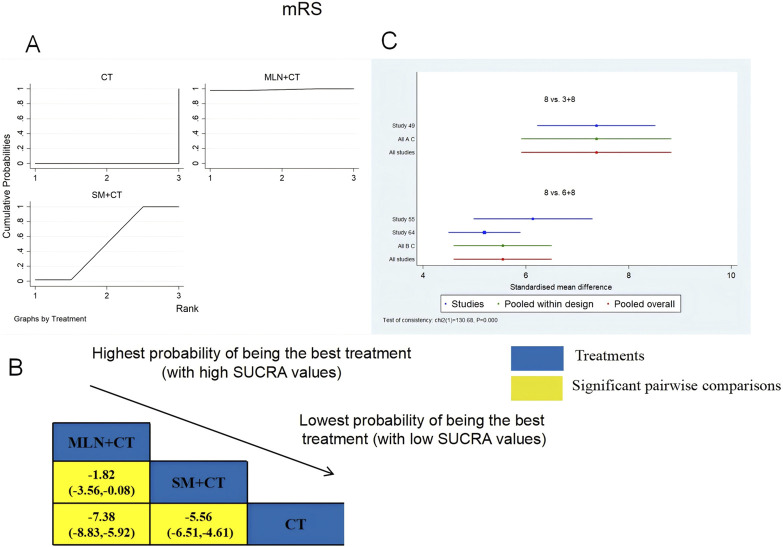
Relative effect sizes of the mRS based on NWA. **(A)** The mRS’ SUCRA graph; **(B)** MDs with the mRS’ 95% CIs; yellow highlights indicate significant pairwise comparisons; **(C)** forest plot of network effect sizes for mRS.

A total of three RCTs examined mRS: MLN + CT vs. CT (n = 1) and SM + CT vs. CT (n = 2). Figure 8 shows the comparisons of MLN + CT vs. CT (MD = −7.38; 95% CI: −8.83 and −5.92), MLN + CT vs. SM + CT (MD = −1.82; 95% CI: −3.56 and −0.08), and SM + CT vs. CT (MD = −5.56, 95% CI: −6.51, −4.61). The SUCRA is shown in Table 1: MLN + CT vs. CT (99.0%), SM + CT vs. CT (51.0%), and CT (0%). Therefore, MLN + CT was most likely the highest ranked intervention for lowering the modified Rankin Scale.

#### 3.4.6 Exploratory analysis of laboratory biomarkers

##### 3.4.6.1 Lipid profiles

###### 3.4.6.1.1 TC

**FIGURE 9:**
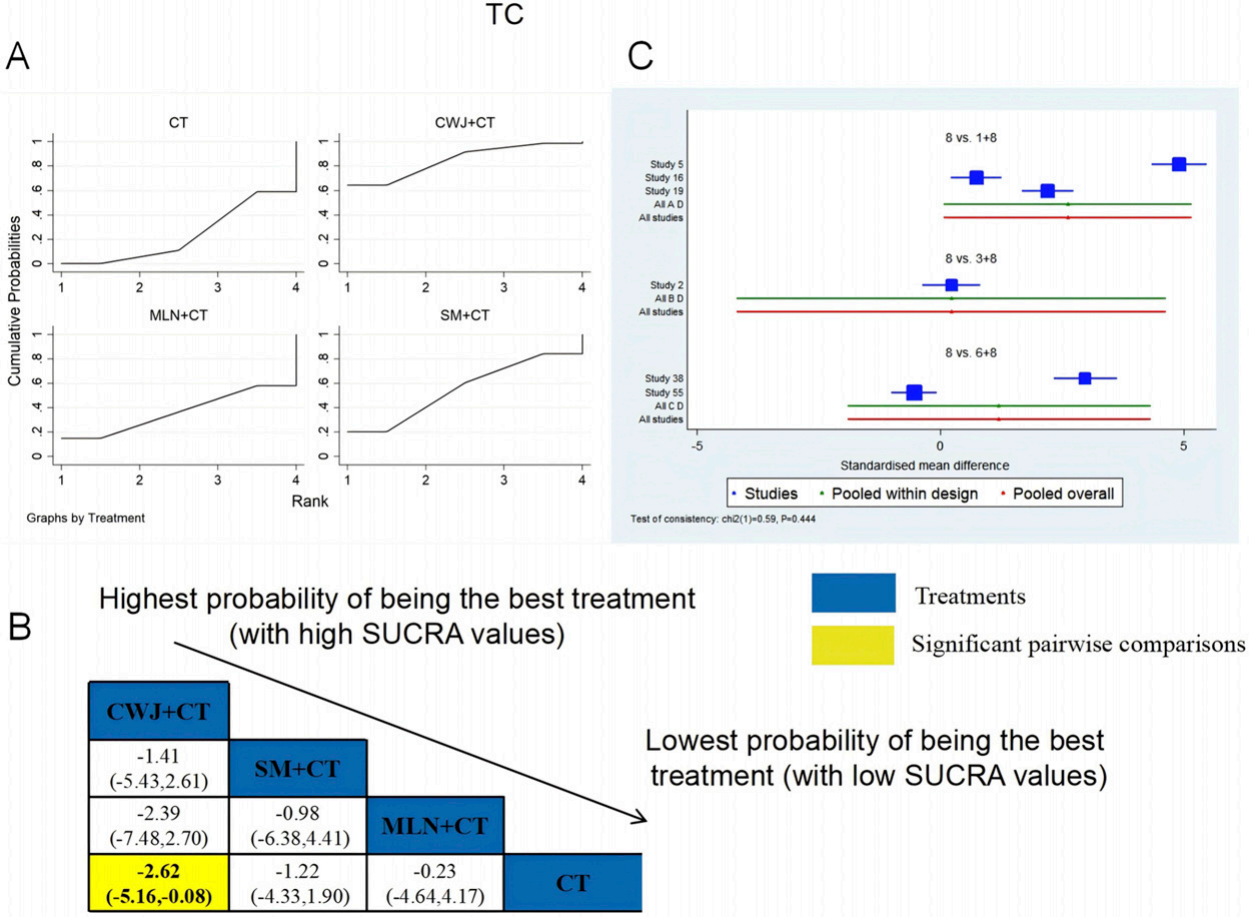
Relative effect sizes of TC based on NWA. **(A)** TC’s SUCRA graph; **(B)** MDs with TC’s 95% CIs; yellow highlights indicate significant pairwise comparisons; **(C)** forest plot of network effect sizes for TC.

A total of six RCTs examined TC: CWJ + CT vs. CT (n = 3), MLN + CT vs. CT (n = 1), and SM + CT vs. CT (n = 2). Figure 9 shows the comparisons of CWJ + CT vs. CT (MD = −2.62; 95% CI: −5.16 and −0.08). When comparing other types of TCMIs pairwise, there were no significant differences. The SUCRA is shown in Table 1: CWJ + CT vs. CT (85.0%), SM + CT vs. CT (55.1%), MLN + CT vs. CT (36.5%), and CT (23.4%). Therefore, treatment with CWJ + CT had the highest probability of being the most successful strategy for decreasing TC levels.

###### 3.4.6.1.2 TG

**FIGURE 10:**
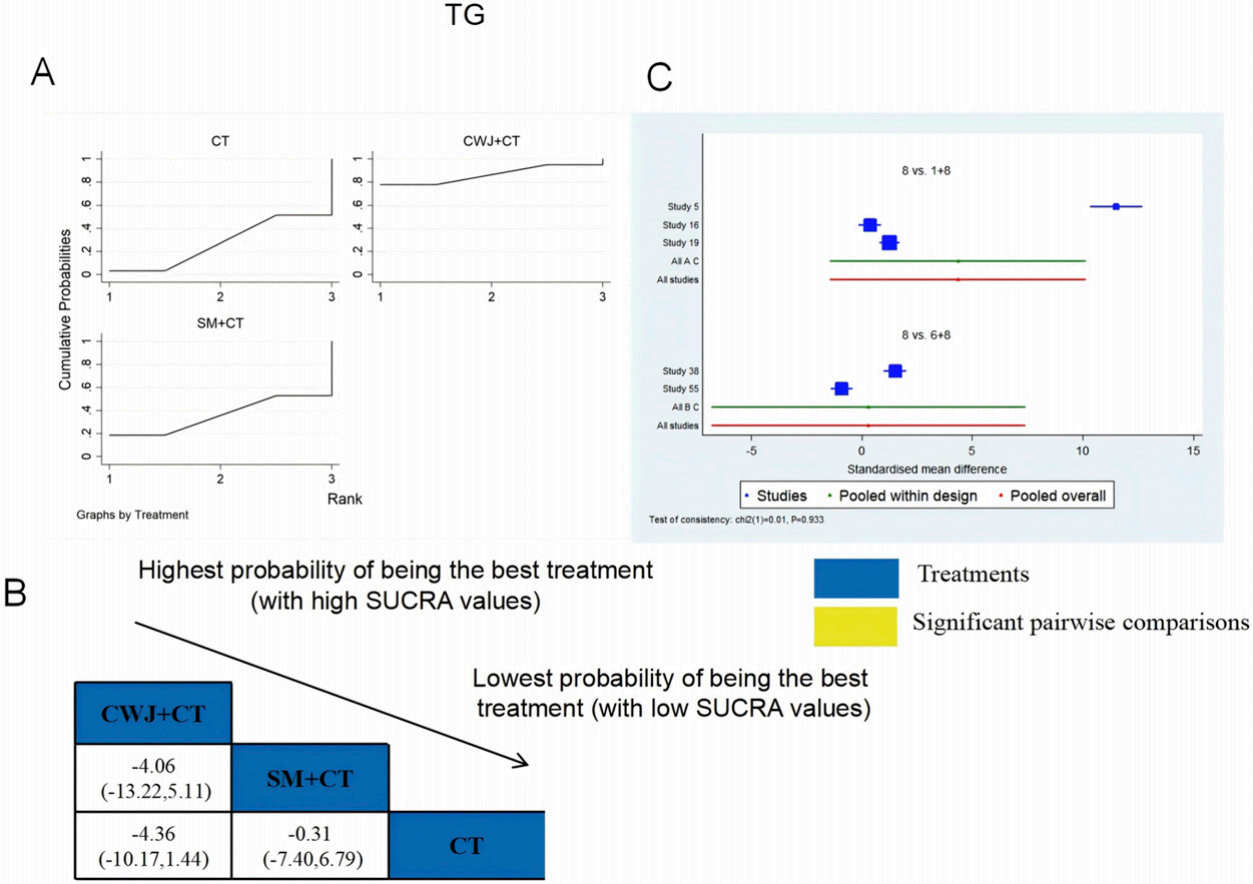
Relative effect sizes of TG based on NWA. **(A)** TG’s SUCRA graph; **(B)** MDs with TG’s 95% CIs; significant pairwise comparisons are highlighted in yellow; **(C)** forest plot of network effect sizes for TG.

A total of six RCTs examined TG: CWJ + CT vs. CT (n = 3), MLN + CT vs. CT (n = 1), and SM + CT vs. CT (n = 2). Figure 10 shows the comparisons of CWJ + CT vs. CT (MD = −2.62, 95% CI: −5.16 and −0.08). The pairwise comparisons of TCMIs showed no discernible differences. The SUCRA is shown in Table 1: CWJ + CT vs. CT (86.6%), SM + CT vs. CT (36.8%), and CT (26.6%). Consequently, CWJ + CT was most likely the highest ranked intervention for lowering TG levels.

###### 3.4.6.1.3 LDL-C

**FIGURE 11:**
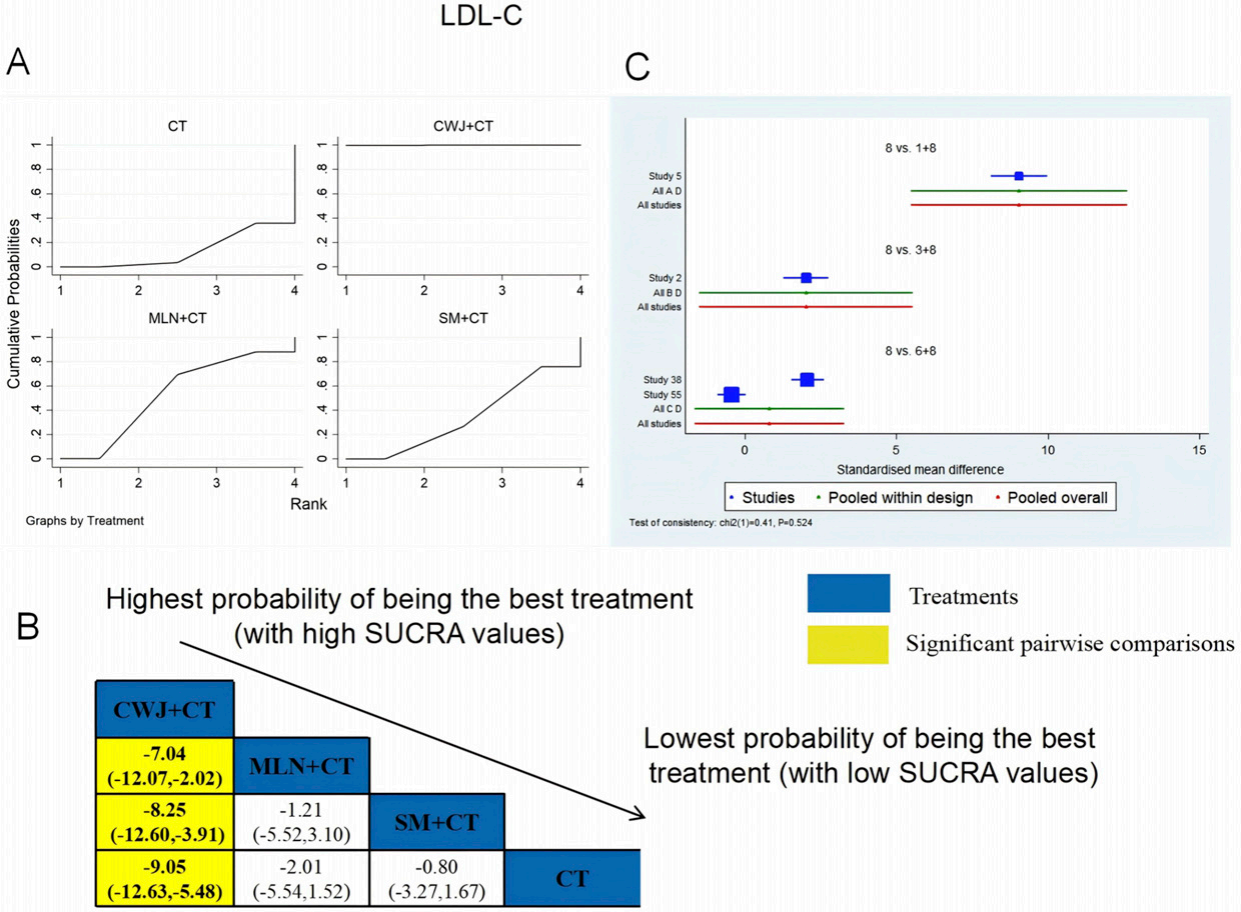
Relative effect sizes of LDL-C based on NWA. **(A)** LDL-C’s SUCRA graph; **(B)** MDs with LDL-C’s 95% CIs; yellow highlights indicate significant pairwise comparisons; **(C)** forest plot of network effect sizes for LDL-C.

A total of four RCTs examined LDL-C: CWJ + CT vs. CT (n = 1), MLN + CT vs. CT (n = 1), and SM + CT vs. CT (n = 2). Figure 11 shows the comparisons of CWJ + CT vs. MLN + CT (MD = −7.04; 95% CI: −12.07 and −2.02), CWJ + CT vs. SM + CT (MD = −8.25; 95% CI: −12.60 and −3.91), and CWJ + CT vs. CT (MD = −9.05; 95% CI: −12.63 and −5.48). The pairwise comparisons of other TCMI types did not reveal any significant differences. The SUCRA is shown in Table 1: CWJ + CT vs. CT (99.9%), MLN + CT vs. CT (52.6%), SM + CT vs. CT (34.3%), and CT (13.1%). Consequently, CWJ + CT was most likely the highest ranked intervention for lowering LDL-C levels.

###### 3.4.6.1.4 HDL-C

**FIGURE 12:**
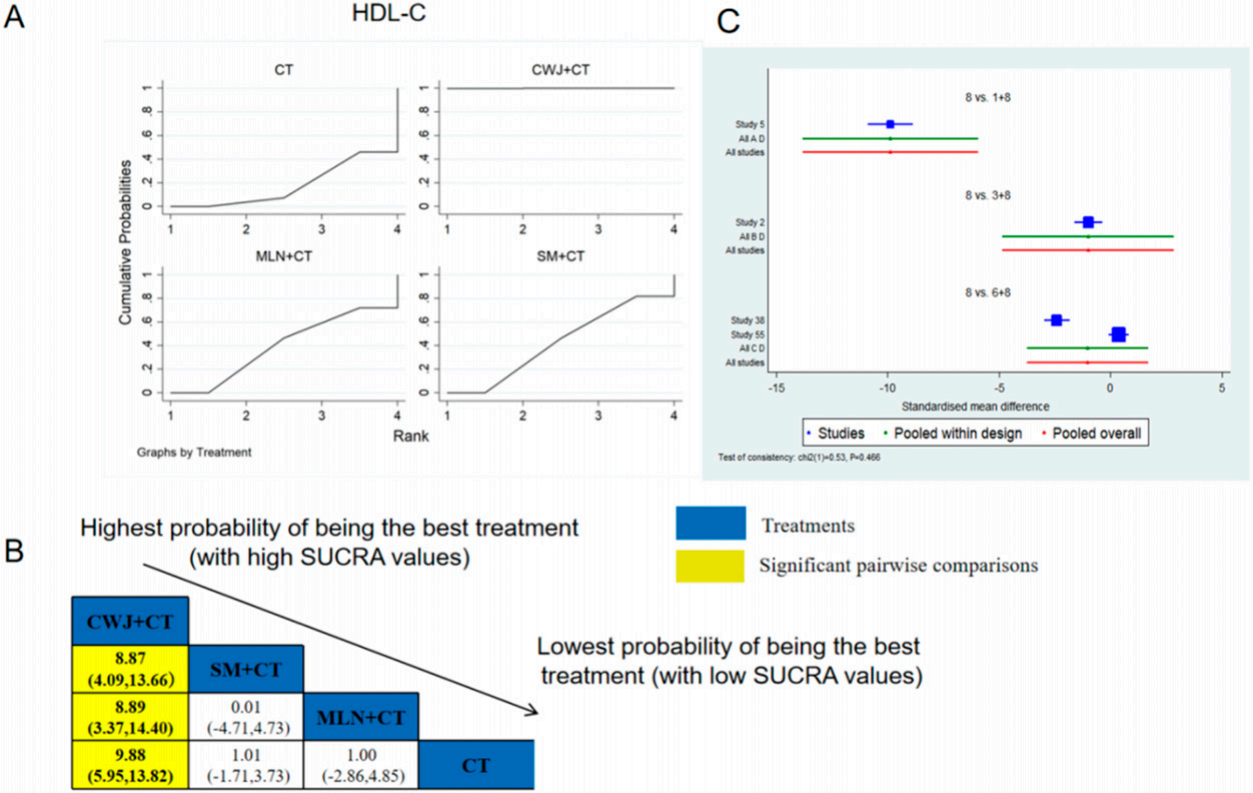
Relative effect sizes of HDL-C based on NWA. **(A)** HDL-C’s SUCRA graph; **(B)** MDs with HDL-C’s 95% CIs; yellow highlights indicate significant pairwise comparisons; **(C)** forest plot of network effect sizes for HDL-C.

A total of four RCTs examined HDL-C: CWJ + CT vs. CT (n = 1), MLN + CT vs. CT (n = 1), and SM + CT vs. CT (n = 2). Figure 12 shows the comparisons of CWJ + CT vs. SM + CT (MD = 8.87; 95% CI: 4.09 and 13.66), CWJ + CT vs. MLN + CT (MD = 8.89; 95% CI: 3.37 and 14.40), and CWJ + CT vs. CT (MD = 9.88; 95% CI: 5.95 and 13.82). In the pairwise comparisons of other TCMI types, there were no significant differences. The SUCRA is shown in Table 1: CWJ + CT vs. CT (100.0%), SM + CT vs. CT (42.7%), MLN + CT vs. CT (39.6%), and CT (17.7%). Consequently, CWJ + CT was most likely the highest ranked intervention for improving HDL-C levels.

##### 3.4.6.2 Hemorheology indices

###### 3.4.6.2.1 HCV

**FIGURE 13:**
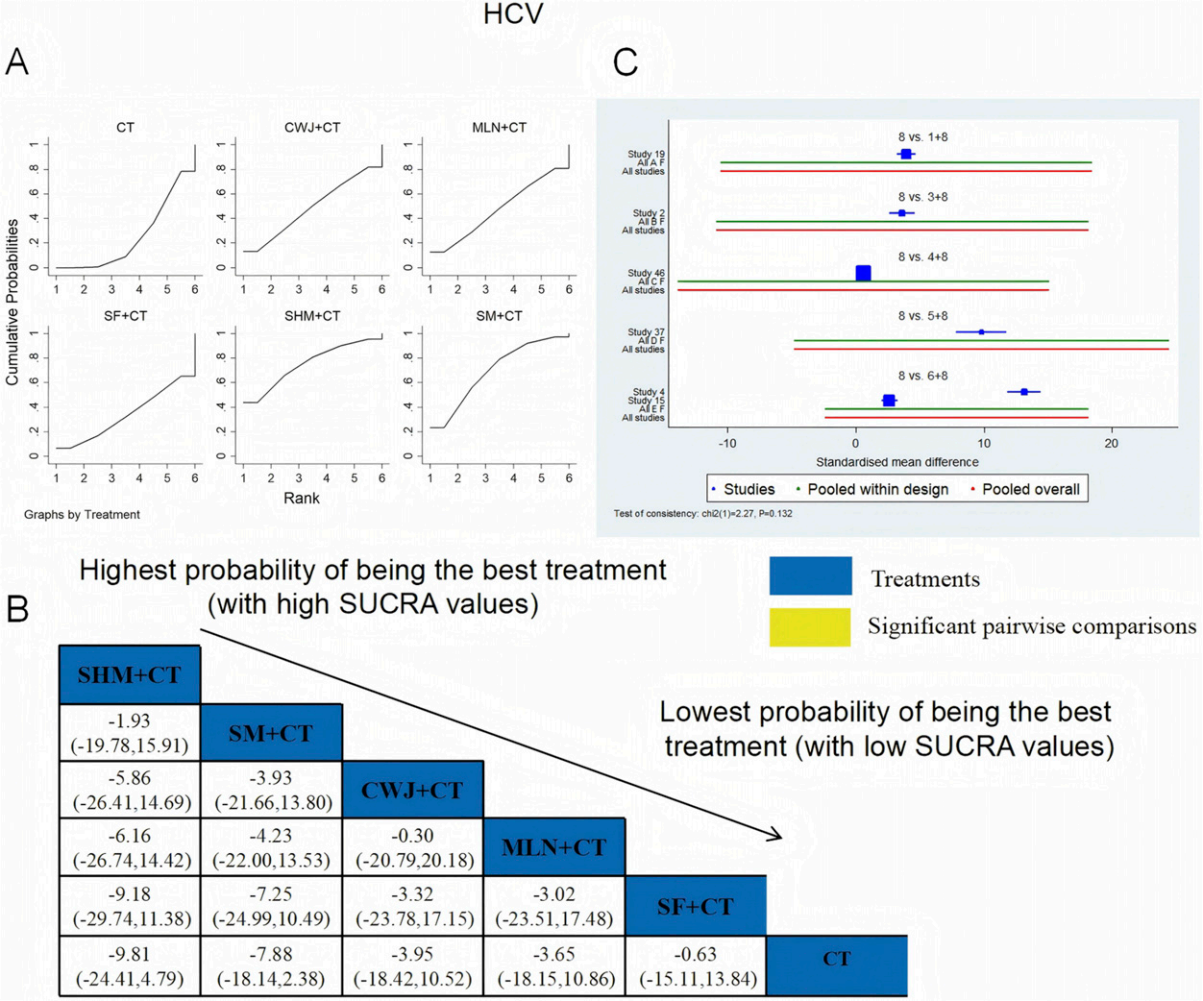
Relative effect sizes of HCV based on NWA. **(A)** HCV’s SUCRA graph; **(B)** MDs with HCV’s 95% CIs; significant pairwise comparisons are highlighted in yellow; **(C)** forest plot of network effect sizes for HCV.

A total of six RCTs examined HCV: CWJ + CT vs. CT (n = 1), MLN + CT vs. CT (n = 1), SF + CT vs. CT (n = 1), SHM + CT vs. CT (n = 1), and SM + CT vs. CT (n = 2). Figure 13 shows that the pairwise comparisons of TCMIs did not reveal any significant differences. The SUCRA is shown in Table 1: SHM + CT vs. CT (75.3%), SM + CT vs. CT (69.7%), CWJ + CT vs. CT (48.9%), MLN + CT vs. CT (47.4%), SF + CT vs. CT (33.8%), and CT (24.9%). Treatment with SHM + CT had the highest probability of being the most successful intervention for lowering HCV levels.

###### 3.4.6.2.2 LCV

**FIGURE 14:**
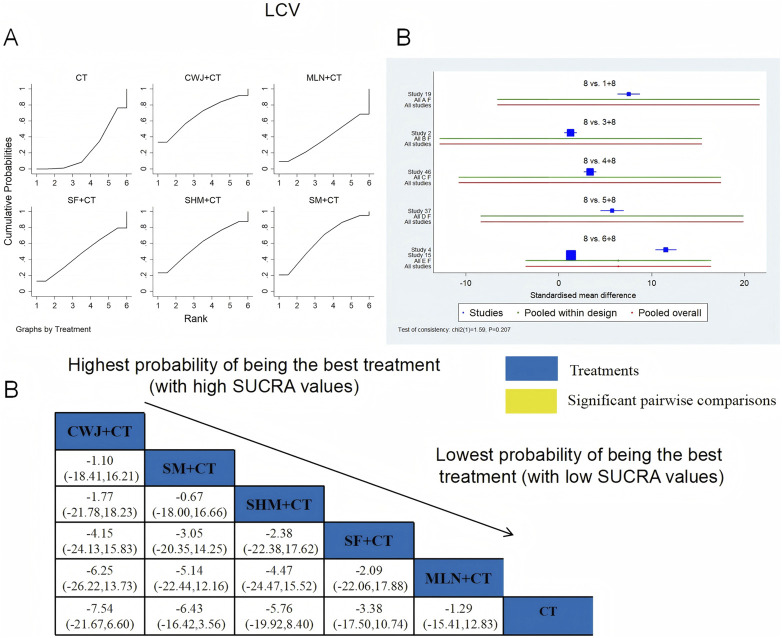
Relative effect sizes of LCV based on NWA. **(A)** LCV’s SUCRA graph; **(B)** MDs with LCV’s 95% CIs; significant pairwise comparisons are highlighted in yellow; **(C)** forest plot of network effect sizes for LCV.

A total of six RCTs examined LCV: CWJ + CT vs. CT (n = 1), MLN + CT vs. CT (n = 1), SF + CT vs. CT (n = 1), SHM + CT vs. CT (n = 1), and SM + CT vs. CT (n = 2). Figure 14 demonstrates that the pairwise comparisons of TCMIs did not reveal any significant differences. The SUCRA is shown in Table 1: SHM + CT vs. CT (59.1%), SM + CT vs. CT (64.3%), CWJ + CT vs. CT (67.7%), MLN + CT vs. CT (37.6%), SF + CT vs. CT (47.1%), and CT (24.2%). Consequently, CWJ + CT had the highest probability of being the most successful intervention for lowering LCV levels.

###### 3.4.6.2.3 PV

**FIGURE 15:**
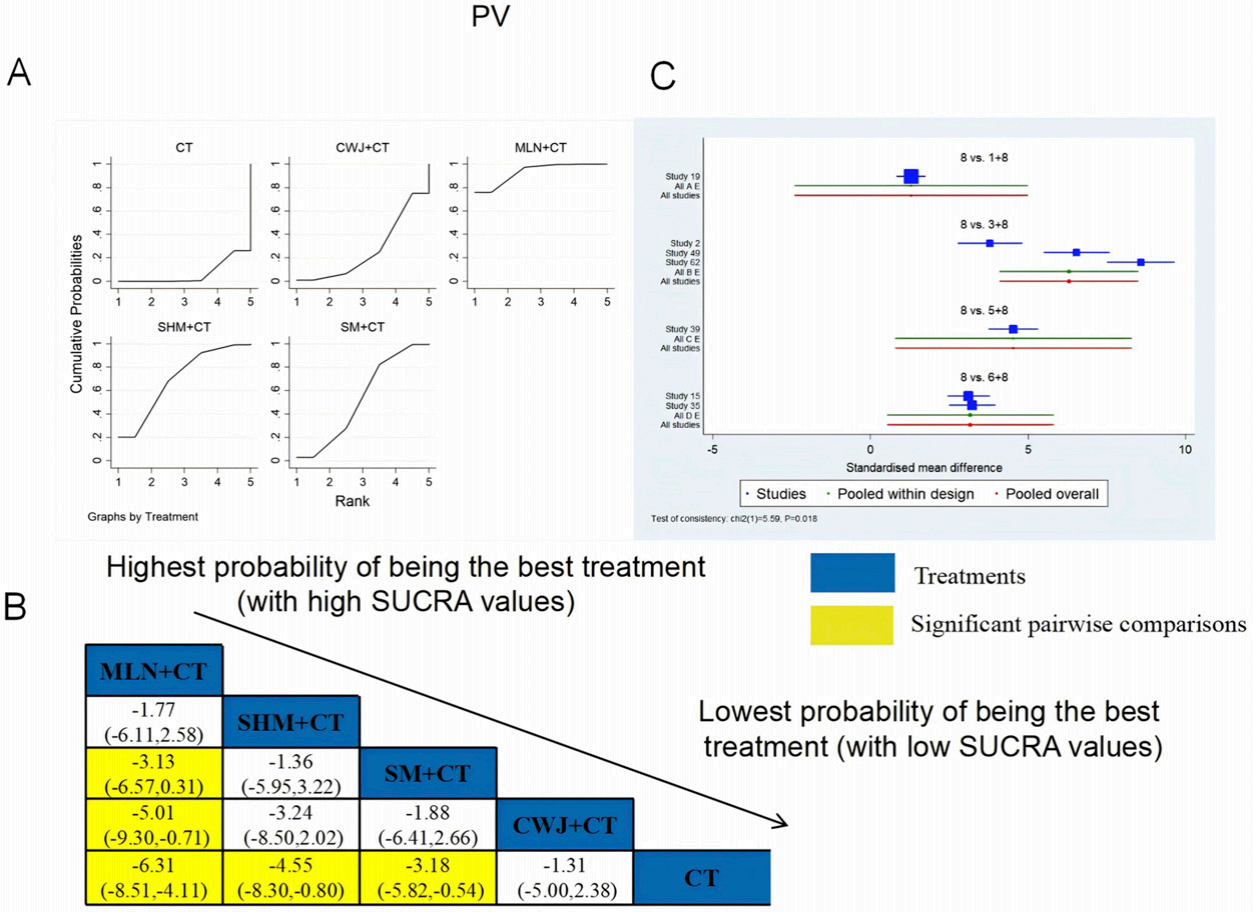
Relative effect sizes of PV based on NWA. **(A)** PV’s SUCRA graph; **(B)** MDs with PV’s 95% CIs; yellow highlights indicate significant pairwise comparisons; **(C)** forest plot of network effect sizes for PV.

A total of seven RCTs examined PV: CWJ + CT vs. CT (n = 1), MLN + CT vs. CT (n = 3), SHM + CT vs. CT (n = 1), and SM + CT vs. CT (n = 2). Figure 15 shows the comparisons of MLN + CT vs. SM + CT (MD = −3.13; 95% CI: −6.57 and 0.31), MLN + CT vs. CWJ + CT (MD = −5.01; 95% CI: −9.30 and −0.71), MLN + CT vs. CT (MD = −6.31; 95% CI: −8.51, −4.11), SHM + CT vs. CT (MD = −4.55; 95% CI: −8.30 and −0.80), and SM + CT vs. CT (MD = −3.18; 95% CI: −5.82 and −0.54). Pairwise comparisons of other TCMI types showed no discernible differences. The SUCRA is shown in Table 1: CWJ + CT vs. CT (26.9%), MLN + CT vs. CT (93.4%), SHM + CT vs. CT (70.0%), SM + CT vs. CT (53.1%), and CT (6.6%). Thus, MLN + CT had the highest probability of being the most effective intervention for lowering PV levels.

###### 3.4.6.2.4 FIB

**FIGURE 16:**
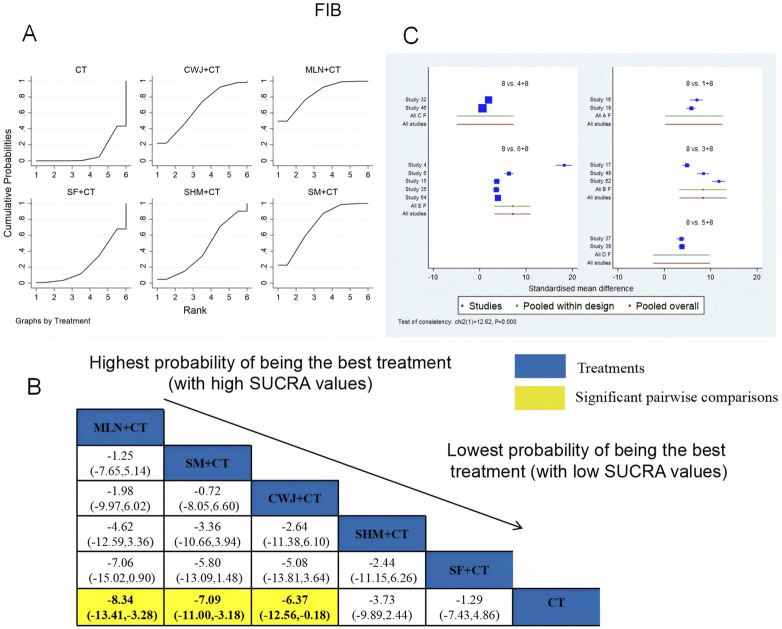
Relative effect sizes of FIB based on NWA. **(A)** FIB’s SUCRA graph; **(B)** MDs with FIB’s 95% CIs; yellow highlights indicate significant pairwise comparisons; **(C)** forest plot of network effect sizes for FIB.

A total of 14 RCTs examined FIB: CWJ + CT vs. CT (n = 2), MLN + CT vs. CT (n = 3), SF + CT vs. CT (n = 2), SHM + CT vs. CT (n = 2), and SM + CT vs. CT (n = 5). Figure 16 shows the comparisons of MLN + CT vs. CT (MD = −8.34; 95% CI: −13.41 and −3.28), SM + CT vs. CT (MD = −7.09; 95% CI: −11.00 and −3.18), and CWJ + CT vs. CT (MD = −6.37; 95% CI: −12.56 and −0.18). The pairwise comparisons of other TCMI types did not reveal any significant differences. The SUCRA is shown in Table 1; SUCRA values (%) of each treatment for outcomes were as follows: CWJ + CT vs. CT (66.6%), MLN + CT vs. CT (83.5%), SF + CT vs. CT (23.7%), SHM + CT vs. CT (43.0%), SM + CT vs. CT (73.6%), and CT (9.6%). Consequently, MLN + CT therapy had the highest probability of being the most successful intervention for reducing FIB.

##### 3.4.6.3 Inflammatory markers

###### 3.4.6.3.1 CRP

**FIGURE 17:**
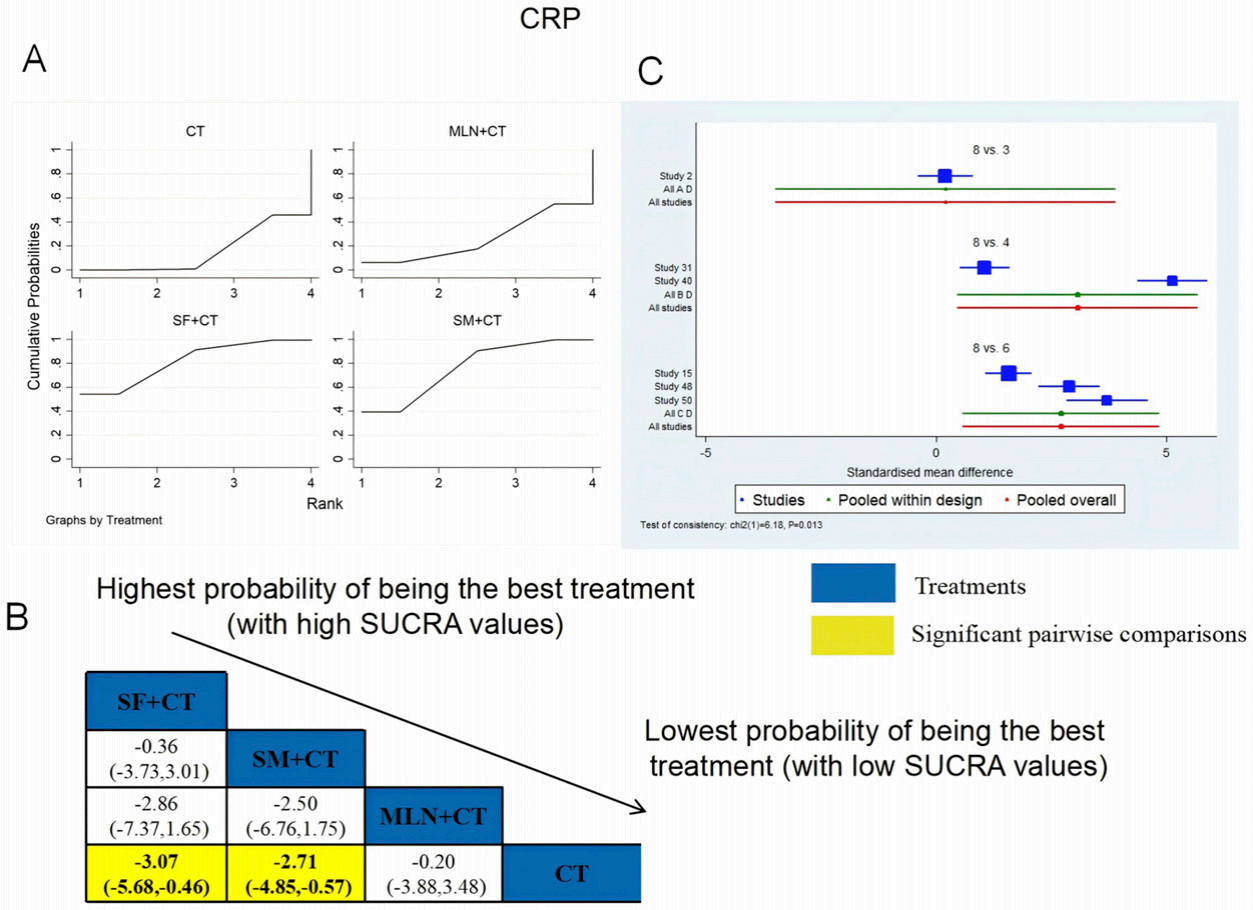
Relative effect sizes of CRP based on NWA. **(A)** CRP’s SUCRA graph; **(B)** MDs with CRP’s 95% CIs; yellow highlights indicate significant pairwise comparisons; **(C)** forest plot of network effect sizes for CRP.

A total of six RCTs examined CRP: MLN + CT vs. CT (n = 1), SF + CT vs. CT (n = 2), and SM + CT vs. CT (n = 3). Figure 17 shows the comparisons of SF + CT vs. CT (MD = −3.07; 95% CI: −5.86 and −0.46) and SM + CT vs. CT (MD = −2.71; 95% CI: −4.85 and −0.57). In the pairwise comparisons of other TCMI types, there were no significant differences. The SUCRA is shown in Table 1: MLN + CT vs. CT (26.2%), SF + CT vs. CT (81.6%), SM + CT vs. CT (76.6%), and CT (15.5%). Thus, SF + CT had the highest probability of being the most effective intervention for lowering CRP levels.

###### 3.4.6.3.2 IL-6

**FIGURE 18:**
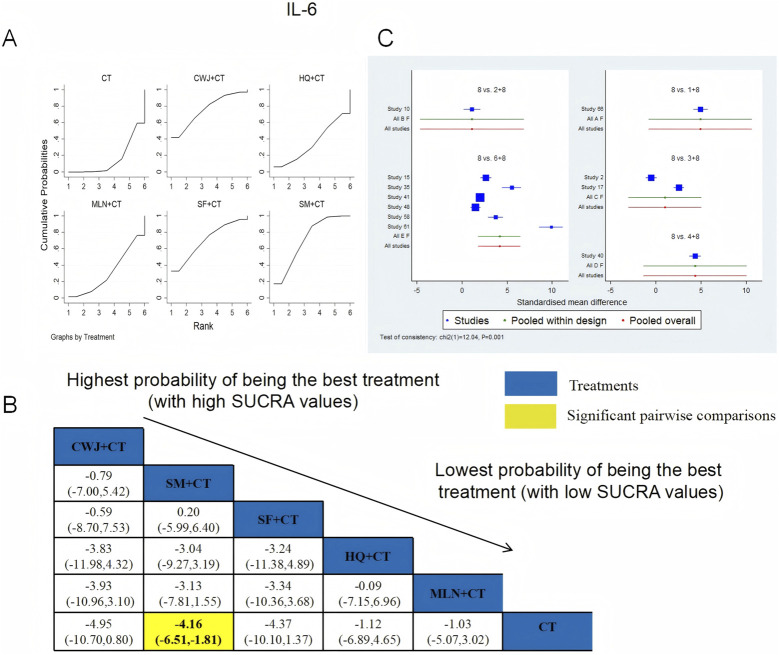
Relative effect sizes of IL-6 based on NWA. **(A)** IL-6’s SUCRA graph; **(B)** MDs with IL-6’s 95% CIs; yellow highlights indicate significant pairwise comparisons; **(C)** forest plot of network effect sizes for IL-6.

IL-6 was examined in 11 RCTs: CWJ + CT vs. CT (n = 1), HQ + CT vs. CT (n = 1), MLN + CT vs. CT (n = 2), SF + CT vs. CT (n = 1), and SM + CT vs. CT (n = 6). Figure 18 shows the comparisons of CWJ + CT vs. CT (MD = −4.95; 95% CI: −10.70 and 0.80) and SM + CT vs. CT (MD = −4.16; 95% CI: −6.51 and −1.81). The pairwise comparisons of the other TCMI types showed no discernible differences. The SUCRA is shown in Table 1: CWJ + CT vs. CT (76.0%), HQ + CT vs. CT (35.2%), MLN + CT vs. CT (31.4%), SF + CT vs. CT (70.4%), SM + CT vs. CT (71.7%), and CT (15.3%). Treatment with CWJ + CT had the highest probability of being the best strategy for reducing IL-6 levels.

###### 3.4.6.3.3 TNF-α

**FIGURE 19:**
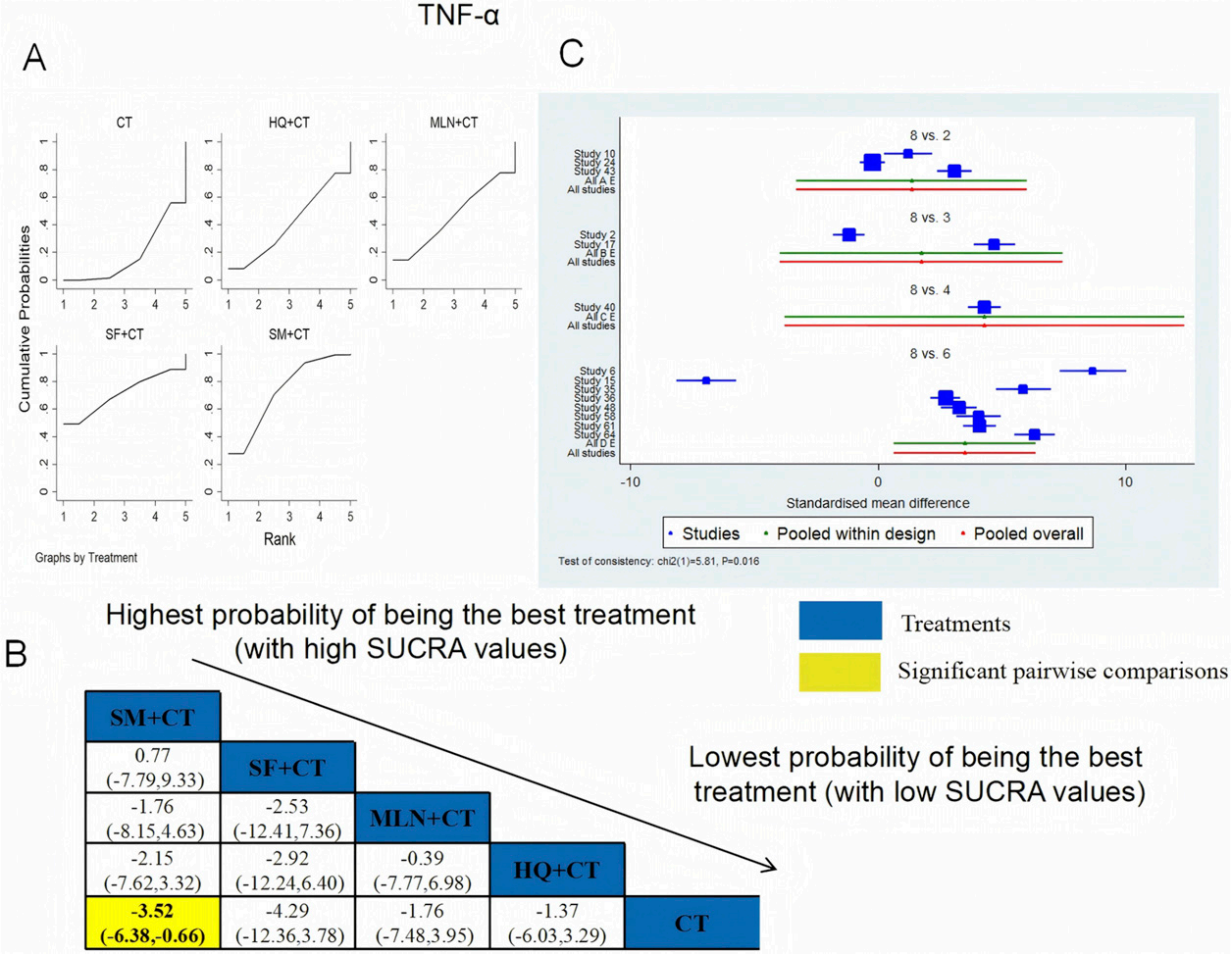
Relative effect sizes of TNF-α based on NWA. **(A)** TNF-α′s SUCRA graph; **(B)** MDs with TNF-α′s 95% CIs; yellow highlights indicate significant pairwise comparisons; **(C)** forest plot of network effect sizes for TNF-α.

TNF-α was examined in 14 RCTs in total: HQ + CT vs. CT (n = 3), MLN + CT vs. CT (n = 2), SF + CT vs. CT (n = 1), and SM + CT vs. CT (n = 8). Figure 19 shows the comparisons of SM + CT vs. CT (MD = −3.52; 95% CI: −6.38 and −0.66). In the pairwise comparisons of other TCMI types, there were no significant differences. The SUCRA is shown in Table 1: HQ + CT vs. CT (40.9%), MLN + CT vs. CT (46.6%), SF + CT vs. CT (71.4%), SM + CT vs. CT (73.0%), and CT (18.2%). Therefore, to reduce the level of TNF-α, SM + CT had the highest probability of being the most successful intervention.

### 3.5 Safety

**FIGURE 20:**
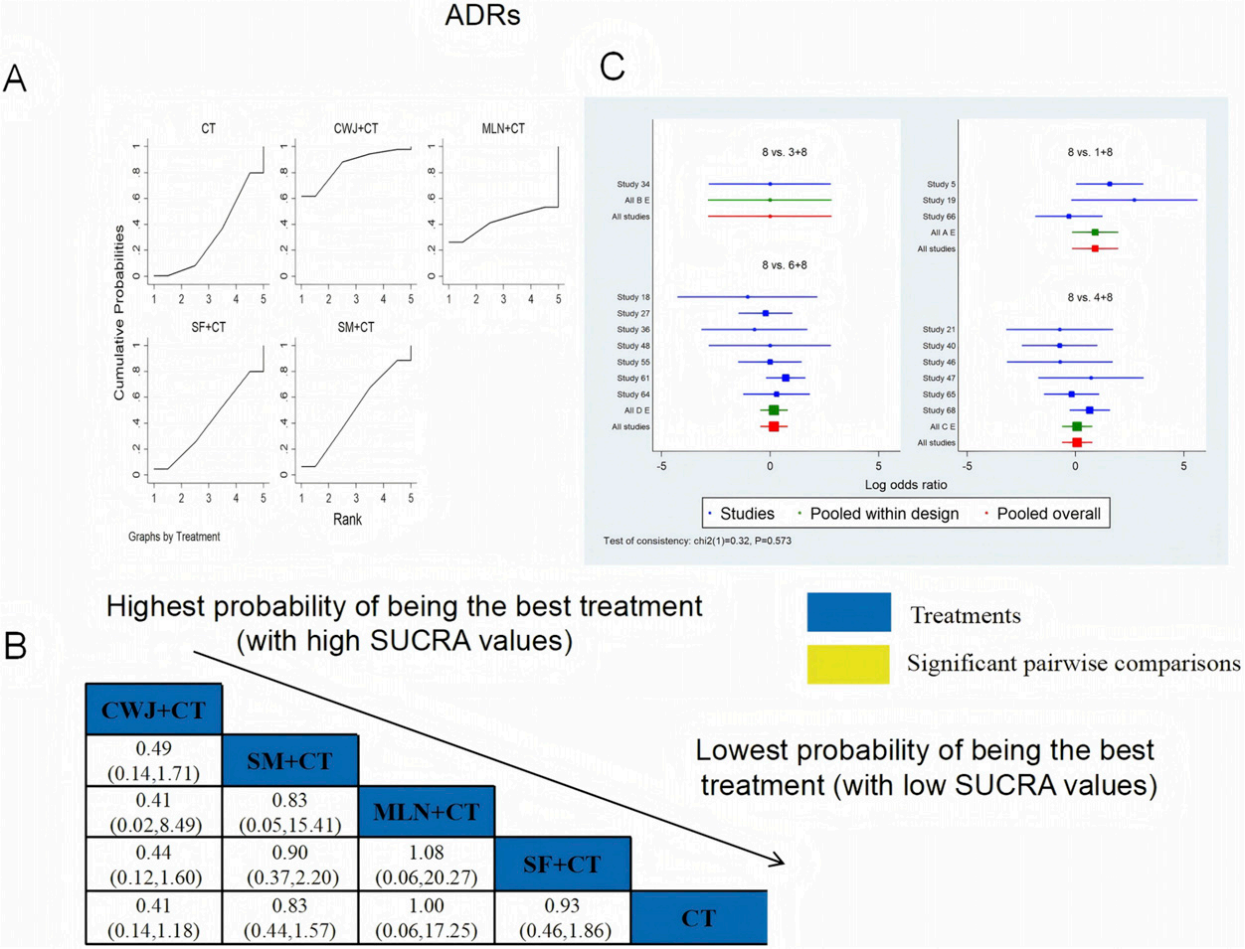
Relative effect sizes of the ADRs based on NWA. **(A)** The ADRs’ SUCRA graph; **(B)** MDs with the ADRs’ 95% CIs; **(C)** forest plot of network effect sizes for ADRs.

Seventeen RCTs examined ADRs: CWJ + CT vs. CT (n = 3), MLN + CT vs. CT (n = 1), SF + CT vs. CT (n = 6), and SM + CT vs. CT (n = 7), with 15 studies detailing specific adverse reactions. These included the following manifestations: headache, palpitations, shortness of breath/chest tightness, nausea/vomiting, diarrhea, skin heating, rash, blooding, and others. The specific results are shown in Table 2. Figure 20 indicates that the pairwise comparisons of TCMIs did not reveal any significant differences. The SUCRA is shown in Table 1: CWJ + CT vs. CT (85.7%), MLN + CT vs. CT (42.3%), SF + CT vs. CT (40.7%), SM + CT vs. CT (49.9%), and CT (31.4%). Consequently, the most effective intervention for enhancing ADRs was most likely CWJ + CT.

**TABLE 2:** Adverse reactions.

Intervention	No. of RCTs (reporting ADRs/total RCTs)	Specific ADRs reported (number of cases)	Reporting of severity/seriousness
CWJ + CT	3/7	• Nausea/vomiting (n = 2) • Skin heating (n = 1) • Others (n = 1)	No study reported the severity or seriousness of ADRs
HQ + CT	0/5	Not reported	Not applicable
MLN + CT	1/9	• Rash (n = 1)	No study reported the severity or seriousness of ADRs
SF + CT	6/16	• Palpitations (n = 2) • Nausea/vomiting (n = 6) • Skin heating (n = 6) • Rash (n = 2) • Blooding (n = 2) • Others (n = 6)	No study reported the severity or seriousness of ADRs
SHM + CT	0/7	Not reported	Not applicable
SM + CT	7/25	• Headache (n = 2) • Shortness of breath/chest tightness (n = 1) • Nausea/vomiting (n = 9) • Skin heating (n = 3) • Rash (n = 3) • Blooding (n = 3) • Others (n = 4)	No study reported the severity or seriousness of ADRs
SQFZ + CT	0/1	Not reported	Not applicable
CT	17/70	• Headache (n = 3) • Palpitations (n = 1) • Shortness of breath/chest tightness (n = 2) • Nausea/vomiting (n = 15) • Diarrhea (n = 13) • Skin heating (n = 1) • Rash (n = 14) • Blooding (n = 10) • Others (n = 9)	No study reported the severity or seriousness of ADRs

### 3.6 Publication bias

Publication bias of this NMA was indicated by the clinical efficacy rate’s nonsymmetrical funnel plot, supplemented by Egger’s regression tests (see Supplementary File 1).

**FIGURE 21:**
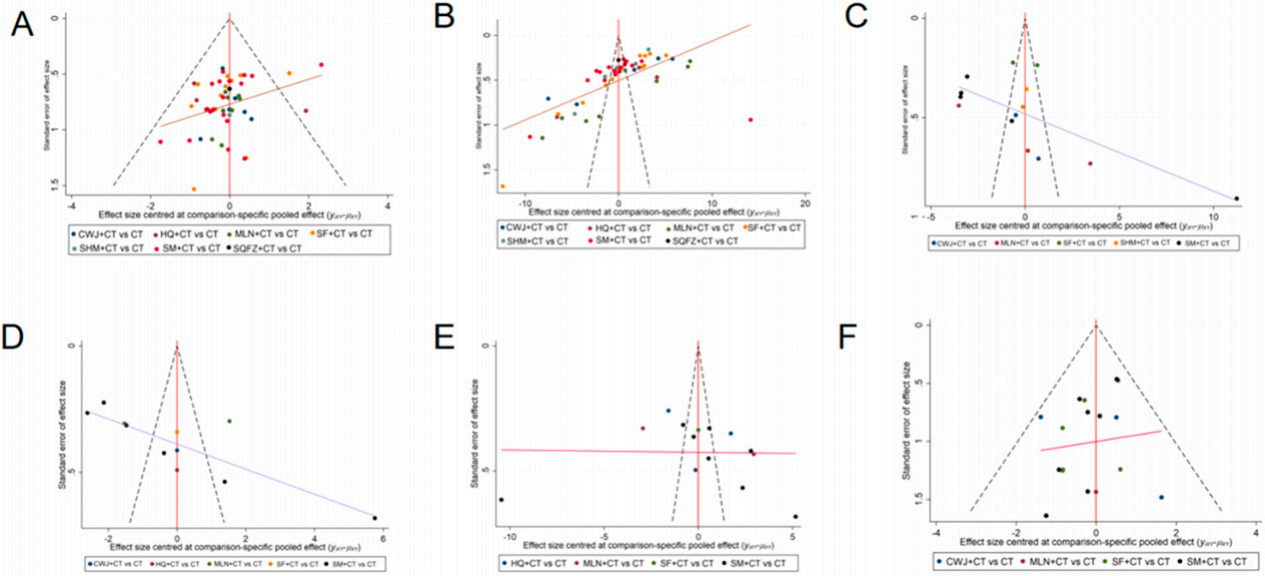
Funnel plot. Note. **(A)** Clinical efficacy rates; **(B)** NIHSS; **(C)** FIB; **(D)** IL-6; **(E)** TNF-α; **(F)** ADRs.

Because the number of RCTs reporting clinical efficacy rates, NIHSS, FIB, IL-6, TNF-α, and ADRs exceeded 10, publication bias was assessed for these outcomes using funnel plots. Dots of various colors represent comparisons between interventions. Figure 21 shows that the funnel plots for clinical efficacy rates, NIHSS, FIB, and IL-6 outcomes were not visually symmetrical, indicating bias. Egger’s regression test shows that TNF-α and ADRs did not have publication bias, whereas other outcomes had. The lack of negative results and large-scale clinical controlled trials may also have helped the bias. Because of this NMA’s non-closed loop, the assumption that direct and indirect evidence were consistent was not applied.

### 3.7 GRADE assessment

Table 3 shows a summary of the evidence quality evaluation of the outcomes conducted using the GRADE method. The evidence was graded from very low to moderate. The most important factor leading to evidence degradation was the risk of bias, followed by imprecision and inconsistency. The included studies were mostly biased in randomization, allocation concealment, and blinding, which suggested that there were common defects in the design of research on TCMIs in the treatment of AIS.

**TABLE 3:** GRADE assessment for the outcomes.

Outcome	No. of studies	Certainty assessment					Summary of findings			Certainty	Importance
							No. of patients		OR/SMD (95% CI)		
		Risk of bias	Inconsistency	Indirectness	Imprecision	Other considerations	TCMIs + CT	CT			
Clinical efficacy rates	52	Serious^a^	Not serious	Not serious	Not serious	None	2,209/2,407 (91.8%)	1,849/2,353 (78.6%)	OR = 3.06 (2.56, 3.65)	⨁⨁⨁○ Moderate	Critical
NIHSS	52	Serious^a^	Not serious	Not serious	Not serious	None	2,360	2,334	SMD = −3.52 (−4.01, −3.04)	⨁⨁⨁○ Moderate	Critical
BI	10	Serious^a^	Serious^b^	Not serious	Not serious	None	517	521	SMD = 3.49 (2.84, 4.15)	⨁⨁○○ Low	Important
ADL	4	Serious^a^	Not serious	Not serious	Not serious	None	211	211	SMD = 2.82 (−2.78, 8.43)	⨁⨁⨁○ Moderate	Important
TC	6	Serious^a^	Not serious	Not serious	Not serious	None	274	272	SMD = −1.76 (−3.39, −0.12)	⨁⨁⨁○ Moderate	Important
TG	5	Serious^a^	Not serious	Not serious	Not serious	None	251	251	SMD = −2.69 (−5.06, −0.31)	⨁⨁⨁○ Moderate	Important
LDL-C	4	Serious^a^	Not serious	Not serious	Serious^c^	None	199	197	SMD = -3.15 (−6.42, 0.12)	⨁⨁○○ Low	Important
HDL-C	4	Serious^a^	Not serious	Not serious	Serious^c^	None	199	197	SMD = 3.20 (−0.10, 6.50)	⨁⨁○○ Low	Important
HCV	6	Serious^a^	Not serious	Not serious	Not serious	None	283	281	SMD = −5.56 (−8.43, −2.70)	⨁⨁⨁○ Moderate	Important
LCV	6	Serious^a^	Not serious	Not serious	Not serious	None	283	281	SMD = −5.11 (−7.84, −2.38)	⨁⨁⨁○ Moderate	Important
PV	7	Serious^a^	Serious^b^	Not serious	Not serious	None	306	304	SMD = −4.42 (−6.14, −2.70)	⨁⨁○○ Low	Important
FIB	41	Serious^a^	Serious^b^	Not serious	Not serious	None	700	702	SMD = −5.88 (−7.45, −4.32)	⨁⨁○○ Low	Important
CRP	6	Serious^a^	Serious^b^	Not serious	Not serious	None	217	215	SMD = −2.41 (−3.76, −1.05)	⨁⨁○○ Low	Important
IL-6	11	Serious^a^	Serious^b^	Not serious	Not serious	None	446	444	SMD = −3.38 (−4.58, −2.17)	⨁⨁○○ Low	Important
TNF-α	14	Serious^a^	Serious^b^	Not serious	Not serious	None	538	559	SMD = −3.44 (−5.21, −1.67)	⨁⨁○○ Low	Important
mRS	3	Serious^a^	Serious^b^	Not serious	Serious^c^	None	152	152	SMD = −5.87 (−6.40, −5.34)	⨁○○○ Very low	Important
ADRs	16	Serious^a^	Not serious	Not serious	Not serious	None	887	859	OR = 0.88 (0.61, 1.28)	⨁⨁⨁○ Moderate	Important

a: the experimental design had a large bias in random, distributive findings or was blind; b: the I2 statistic exceeded 75% in the heterogeneity analysis; c: the confidence interval was not narrow enough, or the simple size is too small.

### 3.8 Tests of inconsistency and heterogeneity

In this study, we did not form a closed loop, so inconsistency tests could not be performed. The results of the heterogeneity analysis showed that there was no significant heterogeneity in the outcome measures such as the clinical efficacy rate (I^2^ = 29%) and ADRs (I^2^ = 42%). In addition, there were too few mRS studies to perform a heterogeneity analysis, and there was significant heterogeneity in the other outcome measures (I^2^ > 75%). More detailed information can be found in Supplementary File 1 in the appendix.

## 4 Discussion

Acute ischemic stroke, which accounts for 70%–80% of all stroke cases, continues to be a major cause of death worldwide (Vasu et al., 2021). Zhengqi is a type of qi that represents the body’s anti-disease substances and anti-disease capabilities. The treatment method of Fuzheng uses Chinese herbs that can replenish and strengthen the body’s energy, thereby improving the body’s condition, enhancing its ability to resist disease, and ultimately preventing and treating disease.

To identify the best option and offer some references for the clinical management of AIS, we conducted an NMA on seven Fuzheng TCMIs and compared the results. A total of 70 RCTs with 6,227 patients were included in this NMA to assess the clinical efficacy rate, NIHSS, BI, ADL, TC, TG, LDL-C, HDL-C, HCV, LCV, PV, FIB, CRP, IL-6, TNF-α, mRS, and ADRs following the use of both CT alone and seven Fuzheng TCMIs in combination. Nevertheless, applying the GRADE methodology to assess the quality of the evidence supporting the findings, we found that clinical efficacy rates, NIHSS, ADL, TC, TG, HCV, LCV, and ADRs were rated as moderate in terms of strength of evidence, with the remaining being low or very low. This indicates that the current conclusions are not very solid, so they should be interpreted with caution. Moreover, the NMA did not form a closed loop, making it impossible to perform consistency checks, which further reduced the reliability of the results.

This NMA suggests that the use of Fuzheng TCMIs, particularly MLN and CWJ injections, alongside CT, may offer benefits in multiple outcome domains for AIS patients, including clinical efficacy, NIHSS, quality of life, ADL, lipid metabolism, hemorheology, inflammatory markers, and safety. However, these findings must be interpreted with substantial caution due to critical methodological limitations within the primary evidence base. Mailuoning injection consists of five botanical drugs in a ratio of 3:3:3:2:1 (Liu et al., 2023). According to the clinical pharmacological research and animal experiments, Mailuoning may enhance blood circulation, shield brain tissue from ischemia injury, and prevent it (Yang et al., 2015). Ciwujia injection is extracted from Araliaceae Acanthopanax, the main active ingredients of which are Acanthopanax glycosides, isofraxidin glucoside, clove glycosides, and Acanthopanax polysaccharides (Hu et al., 2010). Furthermore, investigation of the chemical components of Ciwujia injection using ultrahigh-performance liquid chromatography–quadrupole-electrostatic field orbitrap high-resolution mass spectrometry shows that one of the material bases for the treatment of neurological disorders in Ciwujia injection is phenylpropanoid compounds (Yu et al., 2023).

The BI, ADL, and mRS scales are all commonly used to assess the quality of life in acute stroke patients. Considered a reliable disability scale for stroke patients, the BI is used to measure the patient’s performance in 10 activities of daily living (Quinn et al., 2011). The higher the score, the better the patient’s quality of life (Sulter et al., 1999). In this NMA, we discovered that CWJ + CT had the greatest impact in increasing the BI scale score. The score of mRS is related to the poor functional outcome of patients after stroke (Irie et al., 2025); it evaluates self-reliance rather than task performance. The mRS incorporates both psychological and physical adaptations to neurological deficits (van Swieten et al., 1988) and is divided into six levels, from 0 to 5, with 0 being asymptomatic and 5 being severely disabled. In this NMA, we found that MLN + CT was most likely to be the best intervention for reducing the score of mRS. Understanding the prognosis for the capacity to carry out daily living activities such as dressing, mobility, and bathing is critical to optimizing stroke management in the first few months (Veerbeek et al., 2011). We discovered that the SF + CT had the highest probability of being the most effective method for increasing the ADL score in this NMA.

Although our analysis of core functional outcomes suggests potential benefits of MLN + CT and CWJ + CT, we also conducted exploratory analyses on various laboratory biomarkers to generate hypotheses regarding their possible mechanisms of action. Antiplatelet drugs and lipid-lowering therapy are recommended in the European Stroke Organisation (ESO) guidelines as secondary prevention for stroke (Wardlaw et al., 2024). A significant risk factor for AIS is a high blood lipid level, such as high cholesterol (Shahid et al., 2025), elevated triglyceride–glucose (Oh et al., 2025), elevated low-density lipoprotein (Ding et al., 2025), and high HDL-C levels (Nguyen et al., 2025). In this NMA, we discovered that CWJ ranked the highest for increasing HDL-C and decreasing LDL-C, TC, and TG levels. Because of inadequate vascular autoregulatory mechanisms, AIS is a sudden focal or global neurological impairment brought on by insufficient blood flow to the relevant brain tissue (Uygun et al., 2025). Hemorheology represents the resistance of blood to flow, and abnormal hemorheological indicators, particularly elevated levels of FIB, PV, LCV, and HCV, play a significant role in the occurrence of ischemic stroke (Tikhomirova et al., 2011). The results of this NMA showed that MLN + CT was the most effective at lowering PV and FIB levels, CWJ + CT was the most effective at lowering the level of LCV, and SHM + CT was the most effective at lowering the level of HCV. AIS generates an immune response and is distinguished by the infiltration of immune cells into the brain parenchyma and robust secretion of pro-inflammatory cytokines, which contribute to neuronal loss (DeLong et al., 2022). The markers of this immune response are IL-6 (Zhu et al., 2022), TNF-α (Tuttolomondo et al., 2012), and CRP (Idicula et al., 2009). According to this NMA, CWJ was the most effective at lowering the level of IL-6, SF was the most effective at lowering the level of CRP, and SM was the most effective at lowering the level of TNF-α. It is important to note that these findings on surrogate endpoints should be interpreted with caution and do not constitute direct evidence of clinical efficacy. Their value lies in informing future mechanistic studies.

In addition to clinical efficacy, adequate consideration should be given to TCMI safety in the management of AIS. This NMA reveals a critical gap in the current evidence base regarding the comprehensive safety evaluation of Fuzheng TCMIs. Although our analysis incorporated ADRs as an outcome, only 17 out of the 70 included RCTs (24.3%) systematically reported ADRs, and merely 15 of these provided specific details on adverse events (Table 2). Furthermore, none of the studies that reported ADRs provided details on their severity or whether they were classified as serious adverse events. This profound lack of high-quality, detailed safety data precludes any definitive conclusions regarding the comparative safety of these interventions. Consequently, although the available sparse data did not signal major safety concerns within the reported studies, current evidence is insufficient to robustly characterize the safety of these Fuzheng TCMIs in the treatment of AIS. The safety profiles of several injections, particularly those with fewer trials, remain notably unclear.

Nevertheless, our findings must be interpreted in the context of several important limitations, with the most critical being the methodological quality of the included primary studies. A significant methodological concern is the inadequate reporting of randomization and allocation concealment in the included trials. Among the 70 RCTs, only 36 studies clearly described the method of random sequence generation, whereas the remaining studies either used inappropriate methods (e.g., allocation by order of hospitalization) or merely mentioned the term “random” without further elaboration. Crucially, inadequate reporting and implementation of essential bias-prevention measures were evident: allocation concealment was sufficiently described in only 2/70 studies (2.9%), and some trials had a small number of participants, potentially leading to biased results. The predominant “some concerns” risk rating in bias assessment does not denote methodological neutrality; rather, it signifies critical transparency deficits, whereas unreported methods inherently constitute a risk of bias. Empirical evidence consistently demonstrates that such deficiencies, particularly absent allocation concealment and blinding, systematically inflate treatment effect estimates, especially for subjective endpoints such as NIHSS or mRS scales. Consequently, the ostensibly favorable outcomes for interventions such as MLN + CT and CWJ + CT may represent overestimations of true efficacy. These fundamental flaws necessitated a “serious” risk-of-bias downgrading in GRADE assessments for most outcomes, yielding predominantly low- or moderate-quality evidence. Further diminishing reliability, comprehensive safety data (ADRs) were reported in only 24.3% of trials, and the funnel plot asymmetry suggested possible publication bias. Although observed biological mechanisms for certain TCMIs offer plausibility, they cannot compensate for the clinical evidence’s methodological weaknesses. Additional constraints include geographical limitation to Chinese populations and variable sample sizes. In addition, another methodological limitation of this NMA is the absence of closed loops within the treatment network, which further reduces the reliability of the results. All included RCTs exclusively compared individual Fuzheng TCMIs combined with CT against CT alone; no direct head-to-head RCTs between different TCMIs were available. Although transitivity was supported by clinical homogeneity across studies in terms of patient populations and outcome definitions, the inability to statistically verify consistency introduces uncertainty when interpreting relative efficacy among TCMIs. Moreover, the substantial heterogeneity observed in most outcomes may be attributed to variations in baseline patient characteristics, differences in treatment protocols, inconsistencies in outcome measurement methods or timing, and methodological limitations across included studies.

Therefore, to improve the accuracy of NMA results and provide more effective treatment recommendations, we believe that future clinical studies should adhere to standard RCT designs, improve methodological quality, and provide detailed descriptions of key methodological points when publishing results, thereby enhancing the quality of evidence-based medicine. We hope that future research will include the execution of large-scale, multinational trials encompassing ethnically diverse cohorts and directly comparing active TCMI interventions (e.g., Mailuoning injection vs. Ciwujia injection). Such initiatives are essential to overcome the current limitations of geographic homogeneity and insufficient sample sizes, establish closed loops, validate consistency, and strengthen comparative inference, thereby strengthening external validity and elucidating true treatment effects. Despite certain limitations, this study’s NMA analysis evaluates the effects of different TCMIs on various outcome indicators and provides recommendations for the clinical treatment of AIS.

## 5 Conclusion

Based on the findings of this NMA, it can be concluded that TCMIs in conjunction with CT appear to be associated with improvements in multiple outcome domains for AIS, and that MLN + CT and CWJ + CT are often ranked highly across multiple outcomes, representing promising treatment options, but these findings are not definitive due to limited direct comparisons and significant methodological limitations. Although our findings suggest that certain Fuzheng TCMIs may offer benefits in AIS treatment, these results are constrained by the limited quality and scale of the available evidence. More significantly, to confirm our results, future high-quality, multicenter, double-blind RCTs should be conducted. Furthermore, we must standardize medication, closely monitor TCMI adverse drug reactions, and enhance the caliber of TCMI security evaluation in RCTs.

## Data Availability

The original contributions presented in the study are included in the article/Supplementary Material; further inquiries can be directed to the corresponding authors.

## References

[B1] Alonso-Coello P. Schünemann H. J. Moberg J. Brignardello-Petersen R. Akl E. A. Davoli M. (2018). GRADE evidence to decision (EtD) frameworks: a systematic and transparent approach to making well informed healthcare choices. 1: introduction. Gac. Sanit. 32, 166.e1–166.e10. 10.1016/j.gaceta.2017.02.010 28822594

[B2] Barakzie A. Jansen A. J. G. Cavalcante F. Nagy M. Dippel D. W. J. van der Lugt A. (2025). Association of primary and secondary hemostasis biomarkers with acute ischemic stroke outcome in patients undergoing thrombectomy, with or without thrombolytics: post hoc analysis of the multicenter randomized clinical trial of endovascular treatment for acute ischemic stroke in the Netherlands-NO IV. J. Thromb. Haemost. 23, 235–247. 10.1016/j.jtha.2024.10.008 39442626

[B3] Bogenschutz K. M. Fisher D. S. Wright G. W. (2025). Acute ischemic stroke: a guideline-based overview of evaluation and management. JAAPA 38, 13–20. 10.1097/01.JAA.0000000000000203 40197996

[B4] Chen F. Zhou H. Zhang T. Wang L. Chen H. Hu J. (2025). The efficacy of intensive Statin therapy in acute ischemic stroke following intravenous thrombolysis: the CASE II study. CNS Neurosci. Ther. 31, e70186. 10.1111/cns.70186 39803754 PMC11726118

[B5] DeLong J. H. Ohashi S. N. O’Connor K. C. Sansing L. H. (2022). Inflammatory responses after ischemic stroke. Semin. Immunopathol. 44, 625–648. 10.1007/s00281-022-00943-7 35767089

[B6] Ding Y. Jiang L. Wang T. Chen Y. Pan Y. Li X. (2025). Oxidised low-density lipoprotein and adverse outcome in patients with acute mild ischaemic stroke or high-risk TIA: a secondary analysis of the INSPIRES randomised clinical trial. Stroke Vasc. Neurol., Svn-2024-003664. 10.1136/svn-2024-003664 40010751 PMC12573334

[B7] Hu J. Shang H. Li J. Zhang L. Zhang J. Zheng W. (2010). Adverse drug reactions linked to ciwujia injection: a systematic review of 521 cases. J. Evid. Based Med. 3, 37–43. 10.1111/j.1756-5391.2010.01066.x 21349038

[B8] Idicula T. T. Brogger J. Naess H. Waje-Andreassen U. Thomassen L. (2009). Admission C-reactive protein after acute ischemic stroke is associated with stroke severity and mortality: the “Bergen stroke study.” BMC Neurol. 9, 18. 10.1186/1471-2377-9-18 19400931 PMC2680802

[B9] Irie F. Nakamura K. Matsuo R. Wakisaka Y. Ago T. Kitazono T. (2025). Factors related to sex differences in long-term functional decline after acute ischemic stroke. Sci. Rep. 15, 13400. 10.1038/s41598-025-97668-y 40251216 PMC12008407

[B10] Li L. Shao C. Liu Z. Wu X. Yang J. Wan H. (2022). Comparative efficacy of honghua class injections for treating acute ischemic stroke: a Bayesian network meta-analysis of randomized controlled trials. Front. Pharmacol. 13, 1010533. 10.3389/fphar.2022.1010533 36249799 PMC9554475

[B11] Lim A. Ma H. Ly J. Singhal S. Pan Y. Wang Y. (2024). Comparison of dual antiplatelet therapies for minor, nondisabling, acute ischemic stroke: a bayesian network meta-analysis. JAMA Netw. Open 7, e2411735. 10.1001/jamanetworkopen.2024.11735 38753327 PMC11099682

[B12] Liu S. Wu J.-R. Zhang D. Wang K.-H. Zhang B. Zhang X.-M. (2018). Comparative efficacy of Chinese herbal injections for treating acute cerebral infarction: a network meta-analysis of randomized controlled trials. BMC Complement. Altern. Med. 18, 120. 10.1186/s12906-018-2178-9 29615027 PMC5883592

[B13] Liu X. Fan L. Li J. Bai Z. Wang Y. Liu Y. (2023). Mailuoning oral liquid attenuates convalescent cerebral ischemia by inhibiting AMPK/mTOR-associated apoptosis and promoting CREB/BDNF-mediated neuroprotection. J. Ethnopharmacol. 317, 116731. 10.1016/j.jep.2023.116731 37277084

[B14] Liu Y. Niu P. Ji H. Chen Z. Zhai J. Jin X. (2024). The use of Panax notoginseng saponins injections after intravenous thrombolysis in acute ischemic stroke: a systematic review and meta-analysis. Front. Pharmacol. 15, 1376025. 10.3389/fphar.2024.1376025 38898926 PMC11185952

[B15] Lv W. Qi X. Xu X. Wang Y. Liao J. (2025). The optimal timing for initiating oral anticoagulant in ischemic stroke combined with non-valvular atrial fibrillation patients: a real-world big data analysis. Neurotherapeutics 22, e00574. 10.1016/j.neurot.2025.e00574 40121108 PMC12418420

[B16] Ma Q. Li R. Wang L. Yin P. Wang Y. Yan C. (2021). Temporal trend and attributable risk factors of stroke burden in China, 1990-2019: an analysis for the global burden of disease study 2019. Lancet Public Health 6, e897–e906. 10.1016/S2468-2667(21)00228-0 34838196 PMC9047702

[B17] Ma Z. Zhang H. Zhao F. Li K. Dong N. Sang W. (2024). Safety and effectiveness of Salvia miltiorrhiza and ligustrazine injection for acute cerebral infarction in Chinese population: a PRISMA-compliant meta-analysis. Front. Pharmacol. 15, 1425053. 10.3389/fphar.2024.1425053 39687295 PMC11646771

[B18] McGuinness L. A. Higgins J. P. T. (2021). Risk-of-bias VISualization (robvis): an R package and shiny web app for visualizing risk-of-bias assessments. Res. Synth. Methods 12, 55–61. 10.1002/jrsm.1411 32336025

[B19] Nguyen K. T. K. Xu H. Gaynor B. J. McArdle P. F. O’Connor T. D. Perry J. A. (2025). Impact of conventional stroke risk factors on Early- and late-onset ischemic stroke: a mendelian randomization study. Stroke 56, 640–648. 10.1161/STROKEAHA.124.048015 39993026 PMC11856430

[B20] Ning B. Zhu X. Wu X. Zhu W. Wang R. Qi C. (2024). Efficacy of different traditional Chinese medicine decoctions in the treatment of ischemic stroke: a network meta-analysis. Front. Pharmacol. 15, 1486458. 10.3389/fphar.2024.1486458 39555103 PMC11565597

[B21] Nisar T. Hanumanthu R. Khandelwal P. (2019). Symptomatic intracerebral hemorrhage after intravenous thrombolysis: predictive factors and validation of prediction models. J. Stroke Cerebrovasc. Dis. 28, 104360. 10.1016/j.jstrokecerebrovasdis.2019.104360 31501036

[B22] Oh R. Kim S. Park S. H. Jang M. Cho S. H. Kim J. Y. (2025). Elevated triglyceride-glucose index is a risk factor for cardiovascular events in adults with type 1 diabetes: a cohort study. Cardiovasc Diabetol. 24, 150. 10.1186/s12933-025-02712-w 40176060 PMC11966936

[B23] Page M. J. McKenzie J. E. Bossuyt P. M. Boutron I. Hoffmann T. C. Mulrow C. D. (2021). The PRISMA 2020 statement: an updated guideline for reporting systematic reviews. BMJ 372, n71. 10.1186/s13643-021-01626-4 33782057 PMC8005924

[B24] Quinn T. J. Langhorne P. Stott D. J. (2011). Barthel index for stroke trials: development, properties, and application. Stroke 42, 1146–1151. 10.1161/STROKEAHA.110.598540 21372310

[B25] Shahid M. S. Bourgleh M. S. Alharfi A. Albariqi S. Albalawi L. Alohali R. (2025). High-dose statins for the prevention of recurrent ischemic stroke: a systematic review and meta-analysis of randomized controlled trials. Ann. Saudi Med. 45, 112–128. 10.5144/0256-4947.2025.112 40189852 PMC12542917

[B26] Sulter G. Steen C. De Keyser J. (1999). Use of the barthel index and modified rankin scale in acute stroke trials. Stroke 30, 1538–1541. 10.1161/01.str.30.8.1538 10436097

[B27] Sun L.-C.-Y. Li W.-S. Chen W. Ren Z. Li C.-X. Jiang Z. (2024). Thrombolytic therapy for patients with acute ischemic stroke: systematic review and network meta-analysis of randomized trials. Front. Neurol. 15, 1490476. 10.3389/fneur.2024.1490476 39839875 PMC11746078

[B28] Tikhomirova I. A. Oslyakova A. O. Mikhailova S. G. (2011). Microcirculation and blood rheology in patients with cerebrovascular disorders. Clin. Hemorheol. Microcirc. 49, 295–305. 10.3233/CH-2011-1480 22214701

[B29] Tuttolomondo A. Di Raimondo D. Pecoraro R. Arnao V. Pinto A. Licata G. (2012). Inflammation in ischemic stroke subtypes. Curr. Pharm. Des. 18, 4289–4310. 10.2174/138161212802481200 22390641

[B30] Uygun G. G. Caglar S. E. Tutkavul K. Kosem E. G. Karakoc Y. (2025). Investigation of hemorheological parameters in ischemic stroke patients. Int. J. Lab. Hematol. 47, 166–174. 10.1111/ijlh.14373 39376096

[B31] van Swieten J. C. Koudstaal P. J. Visser M. C. Schouten H. J. van Gijn J. (1988). Interobserver agreement for the assessment of handicap in stroke patients. Stroke 19, 604–607. 10.1161/01.str.19.5.604 3363593

[B32] Vasu S. Luis G. Dileep R. Y. (2021). Global epidemiology of stroke and access to acute ischemic stroke interventions. Neurology 97, S6–S16. 10.1212/WNL.0000000000012781 34785599

[B33] Veerbeek J. M. Kwakkel G. van Wegen E. E. H. Ket J. C. F. Heymans M. W. (2011). Early prediction of outcome of activities of daily living after stroke: a systematic review. Stroke 42, 1482–1488. 10.1161/STROKEAHA.110.604090 21474812

[B34] Wang W. Jiang B. Sun H. Ru X. Sun D. Wang L. (2017). Prevalence, incidence, and mortality of stroke in China: results from a nationwide population-based survey of 480 687 adults. Circulation 135, 759–771. 10.1161/CIRCULATIONAHA.116.025250 28052979

[B35] Wang Y. Li M. Jiang Y. Ji Q. (2024). Comparative efficacy of neuroprotective agents for improving neurological function and prognosis in acute ischemic stroke: a network meta-analysis. Front. Neurosci. 18, 1530987. 10.3389/fnins.2024.1530987 39834702 PMC11743486

[B36] Wardlaw J. M. Chabriat H. Leeuw F.-E. Debette S. Dichgans M. Doubal F. (2024). European stroke organisation (ESO) guideline on cerebral small vessel disease, part 2, lacunar ischaemic stroke. Eur. Stroke J. 9, 5–68. 10.1177/23969873231219416 38380638 PMC10916806

[B37] Xiaoqiang L. Kaimin G. Ruili Z. Wenjia W. He S. Ernesto Y. (2022). Exploration of the mechanism of salvianolic acid for injection against ischemic stroke: a research based on computational prediction and experimental validation. Front. Pharmacol. 13, 894427. 10.3389/fphar.2022.894427 35694259 PMC9175744

[B38] Xiong Y. Li S. Wang C. Sun D. Li Z. Gu H. (2025). Chinese stroke association guidelines on reperfusion therapy for acute ischaemic stroke 2024. Stroke Vasc. Neurol., svn-2024-003977. 10.1136/svn-2024-003977 39832918 PMC12573363

[B39] Xu Y. Cao S. Wang S. Hou X. Guo S. Gou X. (2023). Comparative efficacy and safety of Chinese patent medicines of acute ischemic stroke: a network meta-analysis. Med. 102, e35129. 10.1097/MD.0000000000035129 37861561 PMC10589523

[B40] Yang W. Shi Z. Yang H.-Q. Teng J. Zhao J. Xiang G. (2015). Mailuoning for acute ischaemic stroke. Cochrane Database Syst. Rev. 1, CD007028. 10.1002/14651858.CD007028.pub3 25574904 PMC10839631

[B41] Yin X. Li S. Wang J. Wang M. Yang J. (2025). Research progress of active compounds from traditional Chinese medicine in the treatment of stroke. Eur. J. Med. Chem. 291, 117599. 10.1016/j.ejmech.2025.117599 40188582

[B42] Yu W.-Y. Wu H.-M. Guo X.-J. Yan S.-M. Liu X.-J. Wang Z.-J. (2023). Investigation of the chemical components of ciwujia injection using ultra-high performance liquid chromatography-quadrupole-electrostatic field orbitrap high-resolution mass spectrometry. Se Pu 41, 207–223. 10.3724/SP.J.1123.2022.06005 36861204 PMC9982711

[B43] Zhou D. Xie L. Wang Y. Wu S. Liu F. Zhang S. (2020). Clinical efficacy of tonic traditional Chinese medicine injection on acute cerebral infarction: a bayesian network meta-analysis. Evid. Based Complement. Altern. Med. 2020, 8318792. 10.1155/2020/8318792 33299456 PMC7704142

[B44] Zhou S. Mofatteh M. Chen Z. Tang J. Ma J. Ouyang Z. (2025). Predictive outcome of late window ischemic stroke patients following endovascular therapy: a multi-center study. Front. Neurol. 16, 1489714. 10.3389/fneur.2025.1489714 40212610 PMC11983449

[B45] Zhu Y. Zhang X. You S. Cao X. Wang X. Gong W. (2020). Factors associated with pre-hospital delay and intravenous thrombolysis in China. J. Stroke Cerebrovasc. Dis. 29, 104897. 10.1016/j.jstrokecerebrovasdis.2020.104897 32430238

[B46] Zhu H. Hu S. Li Y. Sun Y. Xiong X. Hu X. (2022). Interleukins and ischemic stroke. Front. Immunol. 13, 828447. 10.3389/fimmu.2022.828447 35173738 PMC8841354

