# Supplementary material for: The efficacy and safety of Fuzheng traditional Chinese medicine injections in the treatment of acute ischemic stroke: a systematic review and network meta-analysis
Source: Front Pharmacol. 2025 Nov 14;16:1658367. doi: 10.3389/fphar.2025.1658367 (PMC12660237; doi:10.3389/fphar.2025.1658367)
Supplement: Supplementary file 1 [file Supplementaryfile1.docx]

**Appendix**

[Appendix Table A.1: PRISMA_2020_checklist 7](#_Toc5164)

[Appendix Table A.2: Composition and Pharmacological effects of Botanical drugs 9](#_Toc6333)

[Appendix Table A.3: Extract and extraction process description of TCMIs 13](#_Toc3000)

[Appendix Table A.4: Details about the product information of TCMIs 15](#_Toc48)

[Appendix Table A.5: Search Strategy for network meta-analysis. 16](#_Toc3135)

[Appendix Table A.6: Egger’s regression test 17](#_Toc30367)

[Appendix Table A.7-A.24: Heterogeneity analysis 18](#_Toc27397)

[Table A.7:Heterogeneity analysis: Clinical effectiveness rate 18](#_Toc5554)

[Table A.8: Heterogeneity analysis: NIHSS 19](#_Toc22207)

[Table A.9: Heterogeneity analysis: BI 21](#_Toc1611)

[Table A.10: Heterogeneity analysis: ADL 21](#_Toc15809)

[Table A.11: Heterogeneity analysis: TC 21](#_Toc10879)

[Table A.12 : Heterogeneity analysis: TG 22](#_Toc14997)

[Table A.13: Heterogeneity analysis: TG 22](#_Toc6229)

[Table A.14: Heterogeneity analysis: LDL-C 22](#_Toc25150)

[Table A.15: Heterogeneity analysis:HDL-C 23](#_Toc29553)

[Table A.16: Heterogeneity analysis: HCV 23](#_Toc27072)

[Table A.17: Heterogeneity analysis: LCV 23](#_Toc25872)

[Table A.18: Heterogeneity analysis:PV 24](#_Toc1175)

[Table A.19: Heterogeneity analysis:PV 24](#_Toc11530)

[Table A.20: Heterogeneity analysis:FIB 24](#_Toc11338)

[Table A.21: Heterogeneity analysis: CRP 25](#_Toc23471)

[Table A.22: Heterogeneity analysis:IL-6 25](#_Toc16705)

[Table A.23: Heterogeneity analysis:TNF-α 26](#_Toc952)

[Table A.24: Heterogeneity analysis:ADRs 26](#_Toc14203)

[Appendix Table A.25-A.38: Sensitivity analysis 27](#_Toc27818)

[Table A.25: Sensitivity analysis graph：NIHSS 27](#_Toc21987)

[Table A.26: Sensitivity analysis graph：BI 31](#_Toc20823)

[Table A.27: Sensitivity analysis graph：ADL 34](#_Toc21348)

[Table A.28: Sensitivity analysis graph：TC 35](#_Toc21496)

[Table A.29: Sensitivity analysis graph：TG 36](#_Toc8970)

[Table A.30: Sensitivity analysis graph：LDL-C 36](#_Toc16865)

[Table A.31: Sensitivity analysis graph：HDL-C 37](#_Toc6846)

[Table A.32: Sensitivity analysis graph：HCV 37](#_Toc24277)

[Table A.33: Sensitivity analysis graph：LCV 38](#_Toc1269)

[Table A.34: Sensitivity analysis graph：PV 38](#_Toc30357)

[Table A.35: Sensitivity analysis graph：FIB 39](#_Toc9757)

[Table A.36: Sensitivity analysis graph：CRP 39](#_Toc24742)

[Table A.37: Sensitivity analysis graph：IL-6 40](#_Toc12125)

[Table A.38: Sensitivity analysis graph：TNF-α 40](#_Toc17246)

Appendix [Table A.39: Basic information included in the study. 41](#_Toc30606)

Appendix Table A.1: PRISMA_2020_checklist

| **Section/topic** | **#** | **Checklist item** | **Reported on page #** |
| --- | --- | --- | --- |
| **TITLE** | | |  |
| Title | 1 | Identify the report as a systematic review incorporating a network meta-analysis (or related form of  meta-analysis). | Title |
| **ABSTRACT** | | |  |
| Structured summary | 2 | Provide a structured summary including, as applicable:  Background: main objectives  Methods: data sources; study eligibility criteria, participants, and interventions; study appraisal; and synthesis methods, such as network meta-analysis.  Results: number of studies and participants identified; summary estimates with corresponding confidence/credible intervals; treatment rankings may also be discussed. Authors may choose to summarize pairwise comparisons against a chosen treatment included in their analyses for brevity.  Discussion/Conclusions: limitations; conclusions and implications of findings.  Other: primary source of funding; systematic review registration number with registry name. | Abstract |
| **INTRODUCTION** | | |  |
| Rationale | 3 | Describe the rationale for the review in the context of what is already known, including mention of why a network meta-analysis has been conducted | Introduction |
| Objectives | 4 | Provide an explicit statement of questions being addressed with reference to participants, interventions, comparisons, outcomes, and study design (PICOS). | Introduction |
| **METHODS** | | |  |
| Protocol and registration | 5 | Indicate if a review protocol exists and if and where it can be accessed (e.g., Web address), and, if available, provide registration information including registration number. | 2. Data and methods |
| Eligibility criteria | 6 | Specify study characteristics (e.g., PICOS, length of follow-up) and report characteristics (e.g., years considered, language, publication status) used as criteria for eligibility, giving rationale. Clearly describe eligible treatments included in the treatment network, and note whether any have been clustered or merged into the same node (with justification). | 2.2. Inclusion and exclusion criteria |
| Information sources | 7 | Describe all information sources (e.g., databases with dates of coverage, contact with study authors to identify additional studies) in the search and date last searched. | 2.1. Literature screening |
| Search | 8 | Present full electronic search strategy for at least one database, including any limits used, such that it could be repeated. | Appendix Table A.5 |
| Study selection | 9 | State the process for selecting studies (i.e., screening, eligibility, included in systematic review, and, if applicable, included in the meta-analysis). | 2.1. Literature screening |
| Data collection process | 10 | Describe method of data extraction from reports (e.g., piloted forms, independently, in duplicate) and any processes for obtaining and confirming data from investigators. | 2.3 Data extraction |
| Data items | 11 | List and define all variables for which data were sought (e.g., PICOS, funding sources) and any assumptions and simplifications made. | 2.3 Data extraction |
| Geometry of the network | 12 | Describe methods used to explore the geometry of the treatment network under study and potential biases related to it. This should include how the evidence base has been graphically summarized for presentation, and what characteristics were compiled and used to describe the evidence base to readers | 2.5 Statistical analysis |
| Risk of bias within individual studies | 13 | Describe methods used for assessing risk of bias of individual studies (including specification of whether this was done at the study or outcome level), and how this information is to be used in any data synthesis. | 2.4 Quality assessment |
| Summary measures | 14 | State the principal summary measures (e.g., risk ratio, difference in means). Also describe the use of additional summary measures assessed, such as treatment rankings and surface under the cumulative ranking curve (SUCRA) values, as well as modified approaches used to present summary findings from meta-analyses. | 2.5 Statistical analysis |
| Planned methods of analysis | 15 | Describe the methods of handling data and combining results of studies for each network meta-analysis. This should include, but not be limited to: Handling of multigroup trials; Selection of variance structure; Selection of prior distributions in Bayesian analyses; and Assessment of model fit. | 2.5 Statistical analysis |
| Assessment of inconsistency | 16 | Describe the statistical methods used to evaluate the agreement of direct and indirect evidence in the treatment network(s) studied. Describe efforts taken to address its presence when found. | 2.5 Statistical analysis |
| Risk of bias across studies | 17 | Specify any assessment of risk of bias that may affect the cumulative evidence (e.g., publication bias, selective reporting within studies) | 2.7 Data synthesis and analysis |
| Additional analyses | 18 | Describe methods of additional analyses if done, indicating which were prespecified. This may include, but not be limited to, the following: Sensitivity or subgroup analyses; Meta-regression analyses; Alternative formulations of the treatment network; and Use of alternative prior distributions for Bayesian analyses (if applicable). | 2.5 Statistical analysis |
| **RESULTS** | | |  |
| Study selection | 19 | Give numbers of studies screened, assessed for eligibility, and included in the review, with reasons for exclusions at each stage, ideally with a flow diagram. | 3.1 Literature selection |
| Presentation of network structure | 20 | Provide a network graph of the included studies to enable visualization of the geometry of the treatment network. | 3.2 Study characteristics |
| Summary of network geometry | 21 | Provide a brief overview of characteristics of the treatment network. This may include commentary on the abundance of trials and randomized patients for the different interventions and pairwise comparisons in the network, gaps of evidence in the treatment network, and potential biases reflected by the network structure. | 3.2Study characteristics |
| Study characteristics | 22 | For each study, present characteristics for which data were extracted (e.g., study size, PICOS, follow-up period) and provide the citations. | Appendix Table A.39 |
| Risk of bias within studies | 23 | Present data on risk of bias of each study and, if available, any outcome level assessment. | 3.3 Bias risk assessment chart |
| Results of individual studies | 24 | For all outcomes considered (benefits or harms), present, for each study: 1) simple summary data for each intervention group, and 2) effect estimates and confidence intervals. Modified approaches may be needed to deal with information from larger networks. | 3.4 Results |
| Synthesis of results | 25 | Present results of each meta-analysis done, including confidence/credible intervals. In larger networks, authors may focus on comparisons versus a particular comparator (e.g., placebo or standard care), with full findings presented in an appendix. League tables and forest plots may be considered to summarize pairwise comparisons. If additional summary measures were explored (such as treatment rankings), these should also be presented. | 3.4 Results |
| Exploration for inconsistency | 26 | Describe results from investigations of inconsistency. This may include such information as measures of model fit to compare consistency and inconsistency models, P values from statistical tests, or summary of inconsistency estimates from different parts of the treatment network. | 3.7 GRADE assessment |
| Risk of bias across studies | 27 | Present results of any assessment of risk of bias across studies for the evidence base being studied. | 3.7 GRADE assessment |
| Results of additional analyses | 28 | Give results of additional analyses, if done (e.g., sensitivity or subgroup analyses, meta-regression analyses, alternative network geometries studied, alternative choice of prior distributions for Bayesian analyses, and so forth). | Appendix Table A.25-A.38 |
| **DISCUSSION** | | |  |
| Summary of evidence | 29 | Summarize the main findings, including the strength of evidence for each main outcome; consider their relevance to key groups (e.g., health care providers, researchers, and policymakers). | 4 Discussion |
| Limitations | 30 | Discuss limitations at study and outcome level (e.g., risk of bias), and at review level (e.g., incomplete retrieval of identified research, reporting bias). Comment on the validity of the assumptions, such as transitivity and consistency. Comment on any concerns regarding network geometry (e.g., avoidance of certain comparisons). | 4 Discussion |
| Conclusions | 31 | Provide a general interpretation of the results in the context of other evidence, and implications for future research. | 5 Conclusion |
| **FUNDING** | | |  |
| Funding | 32 | Describe sources of funding for the systematic review and other support (e.g., supply of data); role of funders for the systematic review. This should also include information regarding whether funding has been received from manufacturers of treatments in the network and/or whether some of the authors are content experts with professional conflicts of interest that could affect use of treatments in the network. | Disclosed in submission system only (per journal policy). |

Appendix Table A.2: **Composition and Pharmacological effects of Botanical drugs**

| **Injection name** | **Botanical drug name** | **Pharmacological effects** |
| --- | --- | --- |
| Ciwujia injection (CWJ) | *Eleutherococcus senticosus* (Rupr. & Maxim.) Maxim.[Araliaceae ,*Eleutherococci radix et rhizoma*] | *From a pharmacological perspective, Acanthopanax senticosus injection ameliorates ischemic brain injury primarily through its multi-target mechanisms. Its active constituents, such as eleutheroside B and total flavonoids, confer neuroprotection by potently inhibiting neuronal apoptosis via the activation of the PI3K/Akt signaling pathway, which subsequently downregulates the Bax/Bcl-2 ratio and cleaved-Caspase-3 expression.*  *Furthermore, it exhibits significant anti-inflammatory effects by suppressing the production of pro-inflammatory cytokines like TNF-α. Concurrently, it promotes neurotrophic support and functional recovery by upregulating the expression of neurotrophic factors (NGF and BDNF) and structural proteins such as F-actin.*  *The collective pharmacological actions—including anti-apoptosis, anti-inflammation, and enhanced neuroregeneration—synergistically contribute to a reduction in cerebral infarct volume and an improvement in neurological and cognitive function following ischemic insult.* |
| Huangqi injection (HQ) | *Astragalus mongholicus* Bunge.[Fabaceae,*Astragali radix*] | *It demonstrates a protective effect on the brain and influences the aging of the nervous system.* |
|  |  |  |
| Mailuoning injection (MLN) | *Achyranthes bidentata* Blume.[Amaranthaceae,*Achyranthis bidentatae radix*] *Scrophularia ningpoensis* Hemsl. [ Scrophulariaceae,*Scrophulariae radix*],  *Dendrobium officinale* Kimura & Migo.[Orchidaceae,*Dendrobii caulis*]  *Lonicera japonica* Thunb. [Caprifoliaceae,*Lonicerae japonicae flos*] |  |
|  |  |  |
|  |  | Pharmacological studies have found that MLN has the effects of lowering blood pressure, dilating blood vessels, improving microcirculation, preventing ischemic damage, and protecting heart and brain tissue. |
|  |  |  |
|  |  |  |
|  |  |  |
|  |  |  |
| Shenfu injection (SF) | *Panax ginseng* C.A.Mey.[Araliaceae,*Ginseng radix et rhizoma rubra*]  *Aconitum carmichaelii* Debeaux.[Ranunculaceae,*Aconiti lateralis radix praeparata*] | *Shenfu Injection demonstrates significant neuroprotective efficacy against cerebral ischemia-reperfusion injury. It ameliorates pathological damage and neurological deficits by attenuating neuronal apoptosis. Furthermore, it confers protection by effectively reducing cerebral edema, achieved through the modulation of key apoptotic and extracellular matrix regulatory pathways.* |
| Shenmai injection (SM) | *Panax ginseng* C.A.Mey.[Araliaceae,*Ginseng radix et rhizoma rubra*]  *Ophiopogon japonicus* (L.f.) Ker Gawl. [Asparagaceae,*Ophiopogonis radix*] | *Shenmai injection demonstrates a protective effect against brain injury through multiple mechanisms. It facilitates hematoma absorption and mitigates the hypoxic response by modulating relevant pathways. Additionally, it exerts anti-inflammatory effects by suppressing the expression of specific pro-inflammatory mediators. A key mechanism involves its potent anti-apoptotic action, achieved by regulating the expression of critical apoptosis-related genes, thereby preserving neuronal survival.* |
|  |  |  |
|  |  |  |
|  |  |  |
|  |  |  |
|  |  |  |
|  |  |  |
|  |  |  |
|  |  |  |
|  |  |  |
|  |  |  |
|  |  |  |
|  |  |  |
|  |  |  |
| Shengmai injection (SHM) | *Panax ginseng* C.A.Mey.[Araliaceae,*Ginseng radix et rhizoma rubra*]  *Ophiopogon japonicus* (L.f.) Ker Gawl. [Asparagaceae,*Ophiopogonis radix*]  *Schisandra chinensis* (Turcz.) Baill.[Schisandraceae,*Schisandrae chinensis fructus*] |  |
|  |  | The ginsenosides abundant in Shengmai Injection can promote the secretion of prostaglandins in the body and prevent the formation of blood clots. Additionally, Ophiopogon japonicus has the ability to eliminate free radicals, while Schisandra chinensis can slow down lipid peroxidation. After receiving treatment with Shengmai Injection, patients experience improved microcirculation, regulated blood pressure and blood lipid levels, thereby enhancing hemodynamics. |
|  |  |  |
|  |  |  |
|  |  |  |
| Shenqifuzheng injection (SQFZ) | *Codonopsis pilosula* (Franch.) Nannf. [Campanulaceae,*Codonopsis radix*]  *Astragalus mongholicus* Bunge.[Fabaceae,*Astragali radix*] | *Pretreatment of adult mesenchymal stem cells with Shenqi Fuzheng Injection prior to transplantation enhances their neuronal differentiation potential in vivo and reduces associated tumorigenic risks, thereby improving safety. Separately, the injection has been demonstrated to ameliorate myocardial fibrosis and exert anti-heart failure effects by suppressing the expression of key collagen types.* |
|  |  |  |
|  |  |  |
|  |  |  |
|  |  |  |
|  |  |  |
|  |  |  |
|  |  |  |

Appendix Table A.3: Extract and extraction process description of TCMIs

| **Injection name** | **Extracts and Extraction Process Description** | **Quality control reported** (Y/N) |
| --- | --- | --- |
| Ciwujia injection (CWJ) | Cool the decoction of *Eleutherococcus senticosus* (Rupr. & Maxim.) Maxim.[Araliaceae ,*Eleutherococci radix et rhizoma*] senticosus to ≤30°C, adjust the pH to 10–12 using lime milk, stir thoroughly, then adjust the pH to 5.0–6.0 using sulfuric acid, stir thoroughly, let stand for over 4 hours, and filter the supernatant; centrifuge the filtrate at high speed; Concentrate the centrifuge supernatant; cool the concentrate to ≤30°C, add ethanol to achieve an ethanol content of 83–85%, stirring continuously while adding, and maintain the system temperature below 30°C; the ethanol addition rate is 60–80 L/min; let it stand for over 12 hours; Filter; centrifuge the filtrate at high speed; take the centrifuge supernatant and perform ultrafiltration using a hollow fiber ultrafiltration membrane with a molecular weight cutoff of 8,000–15,000; further process the resulting ultrafiltrate into Eleutherococcus senticosus injection solution. | Y.  Z20033181 issued by China Food and Drug Administration |
| Huangqi injection (HQ) | Extract *Astragalus mongholicus* Bunge.[Fabaceae,*Astragali radix*] extract, weigh the raw materials according to the formula; Filter the *Astragalus mongholicus* Bunge.[Fabaceae,*Astragali radix*] extract using a titanium rod, boil the filtrate, and cool it; subject the obtained filtrate to microporous pre-filtration, followed by microporous ultrafiltration, add surfactant and mix thoroughly, and adjust the pH to 7.8–8.0; add injectable water to the total volume, mix thoroughly, filter, and fill the filtrate into containers; during filling, pass nitrogen through the system, and sterilize the obtained solution. | Y.  Z20003189 issued by China Food and Drug Administration |
| Mailuoning injection (MLN) | Combine the four herbs—*Achyranthes bidentata* Blume.[Amaranthaceae,*Achyranthis bidentatae radix*],*Scrophularia ningpoensis* Hemsl. [ Scrophulariaceae,*Scrophulariae radix*], *Dendrobium officinale* Kimura & Migo.[Orchidaceae,*Dendrobii caulis*],*Lonicera japonica* Thunb. [Caprifoliaceae,*Lonicerae japonicae flos*]—in the following weight ratios: *Achyranthes bidentata* Blume.[Amaranthaceae,*Achyranthis bidentatae radix*] 10–40%, *Scrophularia ningpoensis* Hemsl. [Scrophulariaceae,*Scrophulariae radix*] 15–35%, *Dendrobium officinale* Kimura & Migo.[Orchidaceae,*Dendrobii caulis*] 40–15%, and *Lonicera japonica* Thunb. [Caprifoliaceae,*Lonicerae japonicae flos*] 35–10%. The mixture is then boiled in water, and the decoction is concentrated into a semi-solid state. Ethanol precipitation is performed, and the ethanol is recovered from the ethanol solution. The extract is then extracted with ethyl acetate, dissolved in distilled water, filtered, and the aqueous solution is adjusted to a pH of 8.5–9.0 with an appropriate amount of Tween-80 and 20% NaOH solution, yielding the final product. | Y.  Z32021102 issued by China Food and Drug Administration |
| Shenfu injection (SF) | *Panax ginseng* C.A.Mey.[Araliaceae,*Ginseng radix et rhizoma rubra*]  is extracted with ethanol, filtered, and the filtrate is mixed with ethanol and allowed to stand. The filtrate is filtered again, and the precipitate is set aside for later use. The ethanol is recovered completely. The filtrate is extracted with diethyl ether, and the aqueous phase is extracted with n-butanol. The n-butanol is recovered, yielding ginsenoside extract; The filter residue is water-extracted, filtered, and the filtrate is boiled with tannic acid, filtered, concentrated, and then ethanol is added and allowed to stand. The filtrate is filtered to collect the precipitate, the ethanol is recovered from the filtrate, the precipitates are combined, dissolved in injectable water, ultrafiltrated, concentrated, and set aside; *Aconitum carmichaelii* Debeaux.[Ranunculaceae,*Aconiti lateralis radix praeparata*] is decocted, filtered, concentrated, and then ethanol is added and allowed to stand. Ethanol is recovered to obtain the *Aconitum carmichaelii* Debeaux.[Ranunculaceae,*Aconiti lateralis radix praeparata*] extract. The polysaccharide solution and extracts are combined, the pH is adjusted, and the solution is filtered to obtain Solution 1. HS-15 is dissolved in injectable water, combined with Solution 1, filtered, and injectable water is added to the full volume. The solution is bottled and sealed, yielding the final product. | Y.  Z20043117 issued by China Food and Drug Administration |
| Shenmai injection (SM) | First, wash the herbs such as *Panax ginseng* C.A.Mey.[Araliaceae,*Ginseng radix et rhizoma rubra*]and *Ophiopogon japonicus* (L.f.) Ker Gawl. [Asparagaceae,*Ophiopogonis radix*] thoroughly, add an appropriate amount of water, and place them in a sandpot for decoction. Filter the decocted liquid and extract the juices from the *Panax ginseng* C.A.Mey.[Araliaceae,*Ginseng radix et rhizoma rubra*]and *Ophiopogon japonicus* (L.f.) Ker Gawl. [Asparagaceae,*Ophiopogonis radix*]. Formulate the herbs such as *Panax ginseng* C.A.Mey.[Araliaceae,*Ginseng radix et rhizoma rubra*] and *Ophiopogon japonicus* (L.f.) Ker Gawl. [Asparagaceae,*Ophiopogonis radix*] in the specified proportions.Place the herbal extracts and the prepared herbal mixture into a concentration tank and concentrate until the desired concentration is achieved.Fill the injection solution into bottles, seal them tightly, and then undergo high-temperature sterilization. | Y.  Z20053302 issued by China Food and Drug Administration |
| Shengmai injection (SHM) | *Schisandra chinensis* (Turcz.) Baill.[Schisandraceae,*Schisandrae chinensis fructus*] was subjected to steam distillation to obtain the distillate (Liquid 1). The residue remaining after steam distillation was decocted with water, and the resulting decoction was filtered, concentrated into a syrup-like substance, precipitated with alcohol, filtered, and the filtrate was concentrated. Water was added, and the mixture was refrigerated to obtain a water precipitate. After precise filtration, the resulting filtrate was the *Schisandra chinensis* (Turcz.) Baill.[Schisandraceae,*Schisandrae chinensis fructus*] water extract (Liquid 2). The same method is used to prepare the *Ophiopogon japonicus* (L.f.) Ker Gawl. [Asparagaceae,*Ophiopogonis radix*] water extract (Liquid 3). *Panax ginseng* C.A.Mey.[Araliaceae,*Ginseng radix et rhizoma rubra*] is macerated in alcohol to obtain the alcohol extract, which is then refrigerated, filtered, and concentrated into a syrup-like substance. Water is added, and the mixture is refrigerated and separated. The filtrate is mixed with an appropriate amount of Tween-80 to obtain the *Panax ginseng* C.A.Mey.[Araliaceae,*Ginseng radix et rhizoma rubra*] water extract (Liquid 4). Mix liquids 2, 3, and 4, add 50% of liquid 1, adjust the pH to 5–8 with NaOH to obtain the initial Shengmai solution, filter the filtrate, heat and sterilize it to obtain the refined filtrate, and finally bottle, sterilize, and test for clarity to obtain the Shengmai injection. | Y.  Z20043172 issued by China Food and Drug Administration |
| Shenqifuzheng injection  (SQFZ) | *Codonopsis pilosula* (Franch.) Nannf. [Campanulaceae,*Codonopsis radix*]  and *Astragalus mongholicus* Bunge.[Fabaceae,*Astragali radix*] water extract, ethanol precipitation once, filtration, ethanol recovery, addition of excipients, pH adjustment, addition of injectable water, filtration through a microporous membrane filter, followed by filtration through a hollow fiber nano-membrane with a molecular weight cutoff of 60,000, filling, sterilization, or freeze-drying, yielding the final product; or: water extraction of *Codonopsis pilosula* (Franch.) Nannf. [Campanulaceae,*Codonopsis radix*] and *Astragalus mongholicus* Bunge.[Fabaceae,*Astragali radix*], filtration, filtration of the filtrate through a microporous membrane, followed by filtration through a hollow fiber nano-membrane with a molecular weight cutoff of 20,000–40,000, addition of excipients, adjustment of pH, sterilization by filtration through a microporous membrane, and finally, filling into vials or freeze-drying to obtain the product. | Y.  Z19990065 issued by China Food and Drug Administration |

**Appendix Table A.4: Details about the product information of TCMIs**

| **Injection name** | **Adverse drug reactions** | **Contraindication or Precautions** |
| --- | --- | --- |
| Ciwujia injection (CWJ) | Not yet clear. | Not yet clear. |
|  |  |  |
| Huangqi injection (HQ) | 1. Systemic effects: allergic reactions, anaphylactic shock, chills, fever, pallor, etc.  2. Respiratory system: dyspnea, cyanosis, asthma, cough.  3. Cardiovascular system: palpitations, chest tightness.  4. Gastrointestinal system: Nausea, vomiting.  5. Skin and appendages: Sweating, rash, itching.  6. Nervous system: Dizziness, headache. | 1. This product is contraindicated for individuals with a history of allergy or severe adverse reactions to this product or formulations containing Astragalus.  2. This product contains polysorbate-80. It is contraindicated for individuals with allergies to polysorbate-80 or similar substances.  3. This product is contraindicated for pregnant women and infants.  4. This product is a warming and nourishing agent. It is contraindicated for individuals with heat syndromes, such as those with external pathogenic factors, qi stagnation and dampness obstruction, food stagnation, yin deficiency with yang hyperactivity, initial stages of abscesses or ulcers with residual heat toxins, as well as those with “excessive heat in the heart and liver, and damp-heat in the spleen and stomach.” |
|  |  |  |
| Mailuoning injection (MLN) | Respiratory system damage, such as difficulty breathing, shortness of breath, laryngeal edema, etc.; systemic damage, such as allergic reactions, chills, fever, anaphylactic shock, etc.; cardiovascular system damage, such as chest tightness, cyanosis, hypotension, hypertension, etc. | Not yet clear. |
|  |  |  |
|  |  |  |
|  |  |  |
|  |  |  |
| Shenfu injection (SF) | Not yet clear. | Not yet clear. |
| Shenmai injection  (SM) | Not yet clear. | Not yet clear. |
| Shengmai injection  (SHM) | Systemic damage, primarily manifested as fever, chills, anaphylactic shock, and allergic reactions; respiratory system damage, primarily manifested as dyspnea, chest tightness, shortness of breath, and laryngeal edema; cardiovascular system damage, primarily manifested as palpitations, cyanosis, arrhythmia, and hypertension; skin and skin appendage damage, primarily manifested as rash and exfoliative dermatitis. | Not yet clear. |
| Shenqifuzheng injection  (SQFZ) | Not yet clear. | Not yet clear. |

**Appendix Table A.5: Search Strategy for network meta-analysis.**

| **PubMed** |
| --- |
| #1 shenqi fuzheng injection[Title] OR huangqi injection[Title] OR ciwujia injection[Title] OR shenfu injection[Title] OR shenmai injection[Title] OR shengmai injection[Title] OR mailuoning injection[Title]  #2 Ischemic Strokes Stroke[Title] OR Ischaemic Stroke[Title] OR Acute Ischemic Stroke[Title] OR Acute Ischemic Strokes[Title] OR Ischemic Stroke[Title] OR Acute Stroke[Title] OR Acute Ischemic Cryptogenic[Title] OR Cryptogenic Stroke[Title] OR Cryptogenic Ischemic Stroke[Title] OR Cryptogenic Embolism Stroke[Title] OR Cryptogenic Embolism Strokes[Title] OR Embolism Stroke[Title] OR Cryptogenic Stroke[Title] OR Cryptogenic Embolism[Title] OR Cryptogenic Stroke[Title] OR Cryptogenic Strokes [Title] OR Cryptogenic Wake-up Stroke [Title] OR Wake up Stroke[Title] OR Wake-up Strokes[Title]  #3 #1 OR #2 |
| **Web of Science** |
| #1 Stroke (Topic) or Acute Ischemic Strokes (Topic) or Ischemic Stroke (Topic) or Acute Stroke (Topic) or Acute Ischemic Cryptogenic (Topic) or Cryptogenic Stroke (Topic) or Cryptogenic Ischemic Stroke (Topic) or Cryptogenic Embolism Stroke (Topic) or Cryptogenic Embolism Strokes (Topic) or Embolism Stroke (Topic) or Cryptogenic Stroke (Topic) or Cryptogenic Embolism (Topic) or Cryptogenic Stroke (Topic) or Cryptogenic Strokes (Topic) or Cryptogenic Wake-up Stroke (Topic) or Wake up Stroke (Topic) or Wake-up Strokes (Topic)  #2 TS=(shenqi fuzheng injection) OR TS=(huangqi injection)OR TS=(ciwujia injection)OR TS=(shenfu injection)OR TS=(shenmai injection)OR TS=(shengmai injection) OR TS=(mailuoning injection)  #3 #1 AND #2 |
| **Embase** |
| #1'Acute Ischemic Strokes':ab,ti OR 'Embolism Stroke' :ti OR 'Cryptogenic Stroke' :ti OR 'Ischemic Stroke' :ti OR 'Wake-up Strokes' :ti OR 'Stroke' :ti OR 'Cryptogenic Embolism Stroke'  #2'mailuoning injection':ti OR 'shengmai injection' :ti OR 'shenmai injection' :ti OR 'ciwujia injection':ti OR 'huangqi injection' :ti OR 'shenfu injection' :ti OR 'shenqi fuzheng injection':ti  #3:#1 AND #2 |
| **Cochrane Library** |
| #1(mailuoning injection):ab,kw,ti OR (shengmai injection):ab,kw,ti OR (shenmai injection):ab,kw,ti OR (ciwujia injection):ab,kw,ti OR (huangqi injection):ab,kw,ti OR (shenfu injection):ab,kw,ti OR (shenqi fuzheng injection):ab,kw,ti  #2(Acute Ischemic Stroke):ab,kw,ti OR (Embolism Stroke):ab,kw,ti OR (Cryptogenic Stroke):ab,kw,ti OR (Ischemic Stroke):ab,kw,ti OR (Wake-up Strokes):ab,kw,ti OR (Stroke):ab,kw,ti OR (Cryptogenic Embolism Stroke):ab,kw,ti  #3:#1 AND #2 |
| **CNKI** |
| （SU %= '急性缺血性脑卒中' OR SU %= '中风急性期' OR SU %= '急性缺血性中风' OR SU %= '急性缺血性脑梗死' OR SU %= '脑梗死急性期' OR SU %= '急性脑梗'） AND （SU %= '参芪扶正注射液' OR SU %= '黄芪注射液' OR SU %= '刺五加注射液' OR SU %= '参附注射液' OR SU %= '参麦注射液' OR SU %= '生脉注射液' OR SU %= '脉络宁注射液'） |
| **WanFang** |
| （题名或关键词:(急性缺血性脑卒中) or 题名或关键词:(中风急性期) or 题名或关键词:(急性缺血性中风) or 题名或关键词:(急性缺血性脑梗死) or 题名或关键词:(脑梗死急性期) or 题名或关键词:(急性脑梗)） and （题名或关键词:(参芪扶正注射液)or 题名或关键词:(参麦注射液) or 题名或关键词:(生脉注射液)or 题名或关键词:(脉络宁注射液) or 题名或关键词:(黄芪注射液) or 题名或关键词:(刺五加注射液) or 题名或关键词:(参附注射液) ） |
| **VIP** |
| （M=(急性缺血性脑卒中 or 中风急性期 or 急性缺血性中风 or 急性缺血性脑梗死 or 脑梗死急性期 or 急性脑梗)） and （M=（参芪扶正注射液 or 黄芪注射液 or 刺五加注射液 or 参附注射液 or 参麦注射液 or 生脉注射液 or 脉络宁注射液）） |
| **SinoMed** |
| ("脉络宁注射液"[标题:智能] OR "生脉注射液"[标题:智能] OR "参麦注射液"[标题:智能] OR "参附注射液"[标题:智能] OR "刺五加注射液"[标题:智能] OR "黄芪注射液"[标题:智能] OR "当归注射液"[标题:智能] OR "参芪扶正注射液"[标题:智能]) AND ("急性缺血性脑卒中"[标题:智能] OR "中风急性期"[标题:智能] OR "急性缺血性中风"[标题:智能] OR "急性缺血性脑梗死"[标题:智能] OR "脑梗死急性期"[标题:智能] OR "急性脑梗"[标题:智能]) |

**Appendix Table A.6:** Egger’s regression test

| **Clinical effectiveness rate**: Number of studies = 52 | | | | Root MSE = 0.38 | | |
| --- | --- | --- | --- | --- | --- | --- |
| Std_Eff | Coef. | Std. Err. | t | | P>\|t\| | 95% CI |
| Slope bias | -0.03 | 0.09 | -0.35 | | 0.73 | -0.20,0.14 |
|  | 0.62 | 0.28 | 2.21 | | 0.03 | 0.06,1.18 |
| **NIHSS**: Number of studies = 52 | | | | Root MSE = 4.33 | | |
| Std_Eff | Coef. | Std. Err. | t | | P>\|t\| | 95% CI |
| Slope bias | 0.75 | 0.71 | 1.05 | | 0.30 | -0.68,2.18 |
|  | -11.48 | 2.15 | -5.35 | | 0.00 | -15.80,-7.17 |
| **FIB**: Number of studies = 14 | | | | Root MSE =2.93 | | |
| Std_Eff | Coef. | Std. Err. | t | | P>\|t\| | 95% CI |
| Slope bias | 2.83 | 0.79 | 3.57 | | 0.004 | 1.10,4.55 |
|  | -18.15 | 2.08 | -8.74 | | 0.000 | -22.67,-13.63 |
| **IL-6**: Number of studies = 11 | | | | Root MSE = 4.74 | | |
| Std_Eff | Coef. | Std. Err. | t | | P>\|t\| | 95% CI |
| Slope bias | 1.61 | 1.68 | 0.96 | | 0.36 | -2.20,5.42 |
|  | -12.91 | 4.98 | -2.59 | | 0.03 | -24.18,-1.64 |
| **TNF-α**: Number of studies =14 | | | | Root MSE = 7.90 | | |
| Std_Eff | Coef. | Std. Err. | t | | P>\|t\| | 95% CI |
| Slope bias | 0.13 | 3.23 | 0.04 | | 0.97 | -6.89,7.16 |
|  | -7.34 | 8.47 | -0.87 | | 0.40 | -25.80,11.11 |
| **ADRs**: Number of studies =17 | | | | Root MSE =0.82 | | |
| Std_Eff | Coef. | Std. Err. | t | | P>\|t\| | 95% CI |
| Slope bias | -0.59 | 0.41 | -1.41 | | 0.18 | -1.47,0.30 |
|  | 0.51 | 0.52 | 0.97 | | 0.35 | -0.60,1.61 |

Appendix Table A.7-A.24: Heterogeneity analysis

Table A.7:Heterogeneity analysis: Clinical effectiveness rate

| Study | Weight (%) | OR (95% CI) |
| --- | --- | --- |
| CAO CY 2016 | 0.6% | 2.09 [0.18, 24.61] |
| CHEN HY 2015 | 15.0% | 0.30 [0.13, 0.68] |
| CHEN JB 2019 | 3.3% | 5.43 [2.25, 13.10] |
| CHEN ZM 2018 | 2.2% | 3.94 [1.30, 11.97] |
| CHENG WY 2011 | 1.0% | 5.52 [1.11, 27.43] |
| CHU SJ 2019 | 1.1% | 4.62 [0.92, 23.04] |
| DENG Y 2004 | 1.4% | 3.27 [0.77, 13.83] |
| DENG ZT 2011 | 0.9% | 3.25 [0.53, 19.82] |
| DONG XF 2020 | 0.6% | 3.17 [0.31, 31.86] |
| FENG ZW 2023 | 1.1% | 4.71 [0.94, 23.67] |
| FU KQ 2010 | 2.0% | 4.76 [1.51, 15.06] |
| FU P 2024 | 0.5% | 9.51 [1.14, 79.60] |
| GAO YM 2015 | 0.3% | 7.76 [0.38, 157.14] |
| HE JX 1998 | 1.4% | 3.96 [0.97, 16.16] |
| HE YP 2001 | 1.4% | 3.88 [0.94, 15.89] |
| HE ZC 2010 | 2.1% | 3.50 [1.11, 11.02] |
| HU LJ 2021 | 1.1% | 4.57 [0.92, 22.73] |
| HU M 2018 | 2.5% | 3.07 [1.02, 9.29] |
| HU RX 2008 | 0.9% | 4.59 [0.84, 25.16] |
| HUANG AH 1999 | 1.0% | 2.62 [0.44, 15.39] |
| HUANG X 2009 | 1.6% | 4.48 [1.30, 15.50] |
| HUANG ZG 2014 | 1.7% | 7.06 [2.21, 22.52] |
| JIANG KJ 2004 | 1.2% | 3.13 [0.60, 16.25] |
| LI HZ 2019 | 0.9% | 5.87 [1.16, 29.83] |
| LI XP 2019 | 1.1% | 4.75 [0.94, 23.98] |
| LIANG XL 2018 | 1.5% | 3.58 [0.89, 14.39] |
| LIU D 2017 | 2.1% | 3.50 [1.06, 11.57] |
| LIU JQ 2013 | 0.4% | 17.50 [1.99, 153.97] |
| LIU R 2020 | 5.0% | 0.21 [0.04, 1.05] |
| LIU YP 2020 | 3.6% | 2.10 [0.77, 5.71] |
| LIU ZH 2024 | 0.6% | 7.79 [0.93, 65.43] |
| LU R 2013 | 2.6% | 2.27 [0.72, 7.16] |
| MO YF 2016 | 1.0% | 5.48 [1.11, 27.22] |
| MU J 2017 | 6.5% | 0.70 [0.27, 1.83] |
| PENG JS 2018 | 1.6% | 3.95 [1.01, 15.39] |
| SHEN AN 2006 | 0.5% | 3.67 [0.35, 38.34] |
| SHEN LY 2016 | 2.6% | 3.02 [1.01, 9.05] |
| SUN XX 2020 | 0.5% | 8.40 [0.98, 72.15] |
| TANG WH 2020 | 1.6% | 3.96 [1.02, 15.48] |
| WANG JX 2014 | 1.0% | 3.55 [0.65, 19.37] |
| WU BR 2009 | 3.8% | 1.73 [0.63, 4.76] |
| WU JL 2023 | 1.0% | 5.00 [0.97, 25.77] |
| YIN WW 2017 | 1.3% | 5.24 [1.30, 21.10] |
| YUAN Z 2017 | 1.6% | 5.57 [1.52, 20.34] |
| ZHAI HZ 2016 | 1.1% | 4.51 [0.91, 22.31] |
| ZHAI YC 2010 | 2.8% | 3.33 [1.21, 9.20] |
| ZHANG BG 2015 | 0.6% | 2.07 [0.18, 24.15] |
| ZHANG FF 2018 | 3.3% | 3.60 [1.41, 9.19] |
| ZHANG L 2023 | 1.1% | 4.59 [0.92, 22.83] |
| ZHANG Y 2015 | 1.0% | 8.29 [1.77, 38.86] |
| ZHANG Y 2018 | 3.3% | 2.40 [0.88, 6.56] |
| ZHEN HX 2019 | 1.0% | 5.40 [1.09, 26.65] |
| Heterogeneity chi-squared = 71.35 (d.f. =51) P =0.03 | | |
| I-squared (variation in SMD attributable to heterogeneity) =29% | | |
| Test of SMD=0 : z= 12.39 P<0.00001 | | |
| Notes：Weights are from fixed effects analysis | | |

Table A.8: Heterogeneity analysis: NIHSS

| Study | Weight (%) | SMD (95% CI) |
| --- | --- | --- |
| CHEN JB 2019 | 1.9% | -6.73 [-7.45, -6.01] |
| CHENG PR 2015 | 1.9% | -4.25 [-5.02, -3.47] |
| CHU SJ 2019 | 2.0% | -2.17 [-2.68, -1.65] |
| DENG QP 2010 | 1.8% | -1.78 [-2.85, -0.71] |
| DONG XF 2020 | 2.0% | -2.37 [-2.96, -1.79] |
| DU XL 2004 | 2.0% | -1.75 [-2.35, -1.15] |
| FENG ZW 2023 | 1.9% | -5.57 [-6.54, -4.61] |
| FU P 2024 | 2.0% | -1.44 [-1.90, -0.97] |
| FU ZH 2008 | 2.0% | -1.82 [-2.42, -1.21] |
| GAO YM 2015 | 1.9% | -2.49 [-3.17, -1.81] |
| GUO SC 2013 | 1.9% | -5.82 [-6.79, -4.85] |
| HE ZC 2010 | 2.0% | -2.12 [-2.76, -1.48] |
| HU M 2018 | 2.0% | -4.14 [-4.80, -3.48] |
| HUANG X 2009 | 2.0% | -1.95 [-2.47, -1.43] |
| HUANG YB 2015 | 1.9% | -2.31 [-2.98, -1.65] |
| HUANG ZG 2014 | 1.9% | -6.15 [-7.07, -5.22] |
| JIANG KJ 2004 | 1.8% | -8.24 [-9.44, -7.05] |
| LI XL 2022 | 1.9% | -2.99 [-3.68, -2.29] |
| LI XP 2019 | 1.9% | -3.48 [-4.18, -2.77] |
| LI YY 2017 | 1.9% | -4.43 [-5.43, -3.43] |
| LIANG XL 2018 | 2.0% | -0.53 [-0.98, -0.08] |
| LIN YQ 2021 | 2.0% | -3.37 [-4.01, -2.72] |
| LIU D 2017 | 2.0% | -4.16 [-4.80, -3.51] |
| LIU H 2016 | 2.0% | -2.28 [-2.75, -1.82] |
| LIU YP 2020 | 1.9% | -4.28 [-4.94, -3.61] |
| LIU ZH 2024 | 1.9% | -5.26 [-6.03, -4.50] |
| LU R 2013 | 1.9% | -3.09 [-3.83, -2.36] |
| MO YF 2016 | 1.9% | -3.37 [-4.06, -2.69] |
| MU J 2017 | 1.9% | -5.26 [-6.18, -4.34] |
| PENG JS 2018 | 1.9% | -4.54 [-5.31, -3.77] |
| SHEN AN 2006 | 2.0% | -1.02 [-1.66, -0.39] |
| SHEN LY 2016 | 2.0% | -2.90 [-3.40, -2.40] |
| SUN XX 2020 | 1.8% | -6.90 [-8.12, -5.67] |
| TANG WH 2020 | 2.0% | -2.06 [-2.56, -1.56] |
| WANG JX 2014 | 2.0% | -1.88 [-2.52, -1.25] |
| WEN J 2009 | 2.0% | -1.65 [-2.27, -1.04] |
| WU FH 2015 | 1.9% | -2.72 [-3.43, -2.01] |
| WU HK 2018 | 1.9% | -7.41 [-8.42, -6.39] |
| WU J 2022 | 1.9% | 5.29 [4.29, 6.30] |
| WU JL 2023 | 1.9% | -4.60 [-5.55, -3.64] |
| XIAO Y 2005 | 1.9% | -2.51 [-3.20, -1.82] |
| YIN WW 2017 | 1.9% | -4.37 [-5.29, -3.46] |
| YU HT 2020 | 2.0% | -2.29 [-2.73, -1.84] |
| YUAN Z 2017 | 1.9% | -8.10 [-9.11, -7.09] |
| ZHAI YC 2010 | 2.0% | -2.90 [-3.36, -2.45] |
| ZHANG BG 2015 | 1.5% | -10.80 [-12.87, -8.74] |
| ZHANG FF 2018 | 1.9% | -5.19 [-5.89, -4.49] |
| ZHANG L 2023 | 1.9% | -3.92 [-4.60, -3.23] |
| ZHANG LH 2013 | 1.9% | -3.68 [-4.37, -2.99] |
| ZHANG WG 2016 | 2.0% | -2.23 [-2.61, -1.84] |
| ZHANG Y 2015 | 1.9% | -5.30 [-6.12, -4.48] |
| ZHANG Y 2018 | 2.0% | -1.12 [-1.59, -0.65] |
| Heterogeneity chi-squared = 1450.74 (d.f. =51) P <0.00001 | | |
| I-squared (variation in SMD attributable to heterogeneity) =96% | | |
| Estimate of between-study variance Tau-squared = 3.04 | | |
| Test of SMD=0 : z= 14.21 P<0.00001 | | |
| Notes：Weights are from random effects analysis | | |

Table A.9: Heterogeneity analysis: BI

| Study | Weight (%) | SMD (95% CI) |
| --- | --- | --- |
| CHEN ZM 2018 | 9.6% | 4.48 [3.71, 5.25] |
| CHENG PR 2015 | 9.4% | 4.72 [3.89, 5.56] |
| CHU SJ 2019 | 10.3% | 2.54 [1.99, 3.09] |
| LI XP 2019 | 10.0% | 3.00 [2.35, 3.65] |
| LIN YQ 2021 | 10.2% | 2.75 [2.17, 3.32] |
| WANG JX 2014 | 9.9% | 2.37 [1.67, 3.06] |
| YU HT 2020 | 10.4% | 3.05 [2.54, 3.56] |
| ZHANG FF 2018 | 9.8% | 5.27 [4.56, 5.98] |
| ZHANG L 2023 | 9.6% | 4.72 [3.94, 5.50] |
| ZHANG WG 2016 | 10.7% | 2.36 [1.97, 2.76] |
| Heterogeneity chi-squared = 101.93 (d.f. =9) P <0.00001 | | |
| I-squared (variation in SMD attributable to heterogeneity) =91% | | |
| Estimate of between-study variance Tau-squared = 1.00 | | |
| Test of SMD=0 : z= 10.94 P<0.00001 | | |
| Notes：Weights are from random effects analysis | | |

Table A.10: Heterogeneity analysis: ADL

| Study | Weight (%) | SMD (95% CI) |
| --- | --- | --- |
| WU HK 2018 | 25.0% | -5.37 [-6.15, -4.59] |
| LU R 2013 | 25.1% | 2.05 [1.44, 2.66] |
| LIU YP 2020 | 25.0% | 6.80 [5.84, 7.75] |
| LIU D 2017 | 24.9% | 7.84 [6.77, 8.91] |
| Heterogeneity chi-squared =554.83 (d.f. =3) P <0.00001 | | |
| I-squared (variation in SMD attributable to heterogeneity) =99% | | |
| Estimate of between-study variance Tau-squared =32.52 | | |
| Test of SMD=0 : z=0.99 P =0.32 | | |
| Notes：Weights are from random effects analysis | | |

Table A.11: Heterogeneity analysis: TC

| Study | Weight (%) | SMD (95% CI) |
| --- | --- | --- |
| CHEN JB 2019 | 16.7% | -4.91 [-5.47, -4.35] |
| DU XL 2004 | 16.7% | -0.74 [-1.27, -0.22] |
| FU P 2024 | 16.7% | -2.22 [-2.75, -1.69] |
| LIANG XL 2018 | 16.6% | -2.99 [-3.63, -2.34] |
| SHEN AN 2006 | 16.6% | -0.23 [-0.83, 0.36] |
| WU J 2022 | 16.8% | 0.54 [0.07, 1.01] |
| Heterogeneity chi-squared = 267.12 (d.f. =5) P <0.00001 | | |
| I-squared (variation in SMD attributable to heterogeneity) =98% | | |
| Estimate of between-study variance Tau-squared = 4.10 | | |
| Test of SMD=0 : z=2.11 P=0.04 | | |
| Notes：Weights are from random effects analysis | | |

Table A.12 : Heterogeneity analysis: TG

| Study | Weight (%) | SMD (95% CI) |
| --- | --- | --- |
| CHEN JB 2019 | 19.4% | -11.52 [-12.69, -10.35] |
| DU XL 2004 | 20.1% | -0.38 [-0.89, 0.13] |
| FU P 2024 | 20.2% | -1.27 [-1.73, -0.82] |
| LIANG XL 2018 | 20.1% | -1.52 [-2.02, -1.02] |
| WU J 2022 | 20.2% | 0.91 [0.42, 1.39] |
| Heterogeneity chi-squared =378.88 (d.f. =4) P <0.00001 | | |
| I-squared (variation in SMD attributable to heterogeneity) =99% | | |
| Estimate of between-study variance Tau-squared = 7.23 | | |
| Test of SMD=0 : z=2.22 P =0.03 | | |
| Notes：Weights are from random effects analysis | | |

Table A.13: Heterogeneity analysis: TG

| Study | Weight (%) | SMD (95% CI) |
| --- | --- | --- |
| CHEN JB 2019 | 19.4% | -11.52 [-12.69, -10.35] |
| DU XL 2004 | 20.1% | -0.38 [-0.89, 0.13] |
| FU P 2024 | 20.2% | -1.27 [-1.73, -0.82] |
| LIANG XL 2018 | 20.1% | -1.52 [-2.02, -1.02] |
| WU J 2022 | 20.2% | 0.91 [0.42, 1.39] |
| CHEN JB 2019 | 19.4% | -11.52 [-12.69, -10.35] |
| Heterogeneity chi-squared =378.88 (d.f. =4) P <0.00001 | | |
| I-squared (variation in SMD attributable to heterogeneity) =99% | | |
| Estimate of between-study variance Tau-squared = 7.23 | | |
| Test of SMD=0 : z=2.22 P =0.03 | | |
| Notes：Weights are from random effects analysis | | |

Table A.14: Heterogeneity analysis: LDL-C

| Study | Weight (%) | SMD (95% CI) |
| --- | --- | --- |
| CHEN JB 2019 | 24.8% | -9.06 [-9.99, -8.12] |
| LIANG XL 2018 | 25.1% | -2.06 [-2.61, -1.52] |
| SHEN AN 2006 | 25.0% | -2.01 [-2.75, -1.27] |
| WU J 2022 | 25.2% | 0.45 [-0.01, 0.92] |
| Heterogeneity chi-squared =319.74 (d.f. =3) P <0.00001 | | |
| I-squared (variation in SMD attributable to heterogeneity) =99% | | |
| Estimate of between-study variance Tau-squared =10.99 | | |
| Test of SMD=0 : z=1.89 P =0.06 | | |
| Notes：Weights are from random effects analysis | | |

Table A.15: Heterogeneity analysis:HDL-C

| Study | Weight (%) | SMD (95% CI) |
| --- | --- | --- |
| CHEN JB 2019 | 24.7% | 9.89 [8.87, 10.90] |
| LIANG XL 2018 | 25.1% | 2.40 [1.82, 2.98] |
| SHEN AN 2006 | 25.1% | 1.00 [0.37, 1.63] |
| WU J 2022 | 25.2% | -0.37 [-0.84, 0.10] |
| Heterogeneity chi-squared =334.47 (d.f. =3) P <0.00001 | | |
| I-squared (variation in SMD attributable to heterogeneity) =99% | | |
| Estimate of between-study variance Tau-squared =11.24 | | |
| Test of SMD=0 : z=1.90 P =0.06 | | |
| Notes：Weights are from random effects analysis | | |

Table A.16: Heterogeneity analysis: HCV

| Study | Weight (%) | SMD (95% CI) |
| --- | --- | --- |
| CHEN HY 2015 | 16.5% | -13.14 [-14.44, -11.85] |
| DONG XF 2020 | 17.0% | -2.65 [-3.26, -2.03] |
| FU P 2024 | 16.9% | -3.95 [-4.67, -3.23] |
| LI YY 2017 | 15.8% | -9.82 [-11.78, -7.86] |
| MU J 2017 | 17.0% | -0.63 [-1.07, -0.19] |
| SHEN AN 2006 | 16.7% | -3.65 [-4.64, -2.66] |
| Heterogeneity chi-squared =402.64 (d.f. =5) P <0.00001 | | |
| I-squared (variation in SMD attributable to heterogeneity) =99% | | |
| Estimate of between-study variance Tau-squared = 12.52 | | |
| Test of SMD=0 : z=3.80 P =0.0001 | | |
| Notes：Weights are from random effects analysis | | |

Table A.17: Heterogeneity analysis: LCV

| Study | Weight (%) | SMD (95% CI) |
| --- | --- | --- |
| CHEN HY 2015 | 16.5% | -11.56 [-12.70, -10.41] |
| DONG XF 2020 | 16.9% | -1.33 [-1.82, -0.84] |
| FU P 2024 | 16.5% | -7.55 [-8.75, -6.35] |
| LI YY 2017 | 16.4% | -5.76 [-6.99, -4.54] |
| MU J 2017 | 16.8% | -3.38 [-4.06, -2.71] |
| SHEN AN 2006 | 16.8% | -1.29 [-1.94, -0.63] |
| Heterogeneity chi-squared =360.66 (d.f. =5) P <0.00001 | | |
| I-squared (variation in SMD attributable to heterogeneity) =99% | | |
| Estimate of between-study variance Tau-squared = 11.44 | | |
| Test of SMD=0 : z=3.66 P =0.0002 | | |
| Notes：Weights are from random effects analysis | | |

Table A.18: Heterogeneity analysis:PV

| Study | Weight (%) | SMD (95% CI) |
| --- | --- | --- |
| DONG XF 2020 | 14.5% | -3.13 [-3.80, -2.46] |
| FU P 2024 | 14.6% | -1.31 [-1.76, -0.85] |
| LI XL 2022 | 14.4% | -3.24 [-3.97, -2.52] |
| LIN YQ 2021 | 14.4% | -4.55 [-5.33, -3.76] |
| SHEN AN 2006 | 14.1% | -3.81 [-4.84, -2.79] |
| TANG WH 2020 | 14.0% | -6.56 [-7.60, -5.52] |
| YUAN Z 2017 | 14.0% | -8.59 [-9.66, -7.53] |
| Heterogeneity chi-squared =216.39 (d.f. =6) P <0.00001 | | |
| I-squared (variation in SMD attributable to heterogeneity) =97% | | |
| Estimate of between-study variance Tau-squared =5.20 | | |
| Test of SMD=0 : z=5.04 P <0.00001 | | |
| Notes：Weights are from random effects analysis | | |

Table A.19: Heterogeneity analysis:PV

| Study | Weight (%) | SMD (95% CI) |
| --- | --- | --- |
| DONG XF 2020 | 14.5% | -3.13 [-3.80, -2.46] |
| FU P 2024 | 14.6% | -1.31 [-1.76, -0.85] |
| LI XL 2022 | 14.4% | -3.24 [-3.97, -2.52] |
| LIN YQ 2021 | 14.4% | -4.55 [-5.33, -3.76] |
| SHEN AN 2006 | 14.1% | -3.81 [-4.84, -2.79] |
| TANG WH 2020 | 14.0% | -6.56 [-7.60, -5.52] |
| YUAN Z 2017 | 14.0% | -8.59 [-9.66, -7.53] |
| Heterogeneity chi-squared =216.39 (d.f. =6) P <0.00001 | | |
| I-squared (variation in SMD attributable to heterogeneity) =97% | | |
| Estimate of between-study variance Tau-squared =5.20 | | |
| Test of SMD=0 : z=5.04 P <0.00001 | | |
| Notes：Weights are from random effects analysis | | |

Table A.20: Heterogeneity analysis:FIB

| Study | Weight (%) | SMD (95% CI) |
| --- | --- | --- |
| CHEN HY 2015 | 6.7% | -18.28 [-20.06, -16.50] |
| CHEN ZM 2018 | 7.1% | -6.34 [-7.35, -5.33] |
| DONG XF 2020 | 7.2% | -3.64 [-4.38, -2.91] |
| DU XL 2004 | 7.0% | -6.98 [-8.36, -5.59] |
| FENG ZW 2023 | 7.2% | -4.81 [-5.67, -3.95] |
| FU P 2024 | 7.2% | -5.78 [-6.73, -4.82] |
| HUANG ZG 2014 | 7.3% | -1.94 [-2.40, -1.47] |
| LI XL 2022 | 7.2% | -3.61 [-4.38, -2.83] |
| LI YY 2017 | 7.2% | -3.63 [-4.50, -2.76] |
| LIN YQ 2021 | 7.3% | -3.83 [-4.52, -3.13] |
| MU J 2017 | 7.3% | -0.63 [-1.07, -0.20] |
| TANG WH 2020 | 7.0% | -8.49 [-9.79, -7.18] |
| YUAN Z 2017 | 6.9% | -11.80 [-13.24, -10.37] |
| ZHANG FF 2018 | 7.3% | -3.97 [-4.54, -3.39] |
| Heterogeneity chi-squared =747.35 (d.f. =13) P <0.00001 | | |
| I-squared (variation in SMD attributable to heterogeneity) =98% | | |
| Estimate of between-study variance Tau-squared =8.65 | | |
| Test of SMD=0 : z=7.37 P <0.00001 | | |
| Notes：Weights are from random effects analysis | | |

Table A.21: Heterogeneity analysis: CRP

| Study | Weight (%) | SMD (95% CI) |
| --- | --- | --- |
| DONG XF 2020 | 17.0% | -1.57 [-2.08, -1.06] |
| HUANG YB 2015 | 16.9% | -1.05 [-1.59, -0.51] |
| LIU D 2017 | 16.5% | -5.12 [-5.87, -4.37] |
| SHEN AN 2006 | 16.8% | -0.20 [-0.80, 0.39] |
| SUN XX 2020 | 16.7% | -2.89 [-3.55, -2.23] |
| WANG JX 2014 | 16.2% | -3.71 [-4.60, -2.83] |
| Heterogeneity chi-squared =137.63 (d.f. =5) P <0.00001 | | |
| I-squared (variation in SMD attributable to heterogeneity) =96% | | |
| Estimate of between-study variance Tau-squared = 2.75 | | |
| Test of SMD=0 : z=3.48 P =0.0005 | | |
| Notes：Weights are from random effects analysis | | |

Table A.22: Heterogeneity analysis:IL-6

| Study | Weight (%) | SMD (95% CI) |
| --- | --- | --- |
| DENG QP 2010 | 8.9% | -1.12 [-2.08, -0.17] |
| DONG XF 2020 | 9.2% | -2.65 [-3.26, -2.03] |
| FENG ZW 2023 | 9.3% | -2.55 [-3.14, -1.97] |
| LI XL 2022 | 8.8% | -5.51 [-6.56, -4.46] |
| LIU D 2017 | 9.2% | -4.37 [-5.03, -3.70] |
| LIU H 2016 | 9.4% | -1.98 [-2.42, -1.54] |
| LIU YP 2020 | 8.5% | -9.88 [-11.21, -8.55] |
| LU R 2013 | 9.1% | -3.74 [-4.57, -2.91] |
| SHEN AN 2006 | 9.2% | 0.50 [-0.10, 1.10] |
| SUN XX 2020 | 9.3% | -1.51 [-2.02, -0.99] |
| ZHANG L 2023 | 9.1% | -4.96 [-5.77, -4.14] |
| Heterogeneity chi-squared =346.40(d.f. =10) P <0.00001 | | |
| I-squared (variation in SMD attributable to heterogeneity) =97% | | |
| Estimate of between-study variance Tau-squared =3.98 | | |
| Test of SMD=0 : z=5.50 P <0.00001 | | |
| Notes：Weights are from random effects analysis | | |

Table A.23: Heterogeneity analysis:TNF-α

| Study | Weight (%) | SMD (95% CI) |
| --- | --- | --- |
| CHEN ZM 2018 | 7.0% | -8.69 [-10.03, -7.36] |
| DENG QP 2010 | 7.1% | -1.22 [-2.20, -0.25] |
| DONG XF 2020 | 7.1% | 6.93 [5.73, 8.13] |
| FENG ZW 2023 | 7.2% | -4.71 [-5.56, -3.87] |
| HE ZC 2010 | 7.3% | 0.21 [-0.30, 0.72] |
| LI XL 2022 | 7.1% | -5.88 [-6.99, -4.77] |
| LI XP 2019 | 6.7% | -13.54 [-15.50, -11.57] |
| LIU D 2017 | 7.2% | -4.27 [-5.04, -3.51] |
| LIU R 2020 | 7.3% | -1.13 [-1.69, -0.57] |
| LIU YP 2020 | 7.2% | -4.11 [-4.75, -3.47] |
| LU R 2013 | 7.2% | -4.07 [-4.95, -3.19] |
| SHEN AN 2006 | 7.2% | 1.17 [0.53, 1.81] |
| SUN XX 2020 | 7.2% | -3.28 [-3.99, -2.57] |
| ZHANG FF 2018 | 7.2% | -6.32 [-7.14, -5.50] |
| Heterogeneity chi-squared =909.90 (d.f. =13) P <0.00001 | | |
| I-squared (variation in SMD attributable to heterogeneity) =99% | | |
| Estimate of between-study variance Tau-squared =11.17 | | |
| Test of SMD=0 : z=3.81 P =0.0001 | | |
| Notes：Weights are from random effects analysis | | |

Table A.24: Heterogeneity analysis:ADRs

| Study | Weight (%) | OR (95% CI) |
| --- | --- | --- |
| CHEN JB 2019 | 14.7% | 0.21 [0.04, 0.98] |
| FU KQ 2010 | 0.8% | 2.82 [0.11, 70.26] |
| FU P 2024 | 10.7% | 0.07 [0.00, 1.22] |
| GAO YM 2015 | 1.6% | 2.07 [0.18, 24.15] |
| HU M 2018 | 7.5% | 1.22 [0.35, 4.26] |
| LI HZ 2019 | 1.6% | 1.00 [0.06, 16.71] |
| LI XP 2019 | 1.8% | 1.63 [0.14, 18.85] |
| LIU D 2017 | 1.9% | 2.79 [0.30, 25.88] |
| LIU YP 2020 | 2.8% | 5.22 [1.08, 25.30] |
| MU J 2017 | 1.6% | 2.05 [0.18, 23.51] |
| PENG JS 2018 | 3.3% | 0.49 [0.04, 5.58] |
| SUN XX 2020 | 1.6% | 1.00 [0.06, 16.61] |
| WU J 2022 | 5.9% | 1.00 [0.23, 4.35] |
| ZHANG FF 2018 | 27.7% | 0.12 [0.03, 0.44] |
| ZHANG L 2023 | 5.0% | 1.47 [0.35, 6.17] |
| ZHANG LH 2013 | 7.3% | 1.20 [0.34, 4.26] |
| ZHANG Y 2015 | 4.3% | 3.14 [0.80, 12.35] |
| Heterogeneity chi-squared =27.61 (d.f. =16) P <0.04 | | |
| I-squared (variation in SMD attributable to heterogeneity) =42% | | |
| Test of SMD=0 : z=0.67 P =0.50 | | |
| Notes：Weights are from fixed effects analysis | | |

Appendix Table A.25-A.38: Sensitivity analysis

Table A.25: Sensitivity analysis graph：NIHSS

Table A.26: Sensitivity analysis graph：BI

Table A.27: Sensitivity analysis graph：ADL

Table A.28: Sensitivity analysis graph：TC

Table A.29: Sensitivity analysis graph：TG

Table A.30: Sensitivity analysis graph：LDL-C

Table A.31: Sensitivity analysis graph：HDL-C

Table A.32: Sensitivity analysis graph：HCV

Table A.33: Sensitivity analysis graph：LCV

Table A.34: Sensitivity analysis graph：PV

Table A.35: Sensitivity analysis graph：FIB

Table A.36: Sensitivity analysis graph：CRP

Table A.37: Sensitivity analysis graph：IL-6

Table A.38: Sensitivity analysis graph：TNF-α

Table A.39: Basic information included in the study.

| **No.** | **Study** | **Design** | **Average age(yr)** | **Sample-size** | **Interventions** | **Course** | **Outcome** |
| --- | --- | --- | --- | --- | --- | --- | --- |
| **1** | CAO CY 2016[1] | RCT | - | T:25 C:25 | T:SM+CT  C:CT | 14d | ① |
| **2** | SHEN AN 2006[2] | RCT | T：68.78±8.31 C：66.24±8.57 | T:23 C:21 | T:MLN+CT  C:CT | 14d | ①②⑤⑦⑧⑨⑩⑪⑬⑭⑮ |
| **3** | SHEN LY 2016[3] | RCT | T：72.49±6.53 C：71.35±5.81 | T:63 C:63 | T:SF+CT  C:CT | 14d | ①② |
| **4** | CHEN HY 2015[4] | RCT | T：60.27±5.97 C：62.32±5.24 | T:106 C:106 | T:SM+CT  C:CT | 14d | ①⑨⑩⑫ |
| **5** | CHEN JB 2019[5] | RCT | - | T:100 C:100 | T:CWJ+CT  C:CT | 20d | ①②⑤⑥⑦⑧⑰ |
| **6** | CHEN ZM 2018[6] | RCT | T：78.47±5.62 C：78.91±6.13 | T:47 C:47 | T:SM+CT  C:CT | 14d | ①③⑫⑮ |
| **7** | CHENG PR 2015[7] | RCT | T：78.47±5.62 C：78.91±6.13 | T:44 C:42 | T:SHM+CT  C:CT | 20d | ②③ |
| **8** | CHENG WY 2011[8] | RCT | - | T:40 C:40 | T:SM+CT  C:CT | 10d | ① |
| **9** | CHU SJ 2019[9] | RCT | T：67.8±5.4 C：68.2±5.3 | T:47 C:47 | T:SM+CT  C:CT | 14d | ①②③ |
| **10** | DENG QP 2010[10] | RCT | T：74.50±4.40 C：73.80±3.61 | T:10 C:10 | T:HQ+CT  C:CT | 7d | ②⑭⑮ |
| **11** | DENG Y 2004[11] | RCT | T：65 C：66 | T:30 C:30 | T:HQ+CT  C:CT | 20d | ① |
| **12** | DENG ZT 2011[12] | RCT | T：62.4 C：60.2 | T:28 C:20 | T:SM+CT  C:CT | 10-14d | ① |
| **13** | ZHAI HZ 2016[13] | RCT | T：52.85±5.15 C：53.42±5.71 | T:55 C:55 | T:SF+CT  C:CT | 14d | ① |
| **14** | ZHAI YC 2010[14] | RCT | T：62.4 C：60.2 | T:86 C:70 | T:SF+CT  C:CT | 15d | ①② |
| **15** | DONG XF 2020[15] | RCT | T：59.38±6.40 C：60.05±6.18 | T:39 C:39 | T:SF+CT  C:CT | 4w | ①②⑨⑩⑪⑫⑬⑭⑮ |
| **16** | DU XL 2004[16] | RCT | T：68.9±7.8 C：67.3±6 | T:30 C:30 | T:CWJ+CT  C:CT | 14d | ②⑤⑥⑫ |
| **17** | FENG ZW 2023[17] | RCT | T：53.00±4.70 C：53.14±4.76 | T:42 C:42 | T:MLN+CT  C:CT | 10d | ①②⑫⑭⑮ |
| **18** | FU KQ 2010[18] | RCT | - | T:84 C:78 | T:SM+CT  C:CT | 14d | ①⑰ |
| **19** | FU P 2024[19] | RCT | T：52.29±3.46 C：52.23±3.81 | T:45 C:45 | T:CWJ+CT  C:CT | 14d | ①②⑤⑰⑥⑨⑩⑪⑫ |
| **20** | FU ZH 2008[20] | RCT | - | T:32 C:28 | T:MLN+CT  C:CT | 14d | ② |
| **21** | GAO YM 2015[21] | RCT | T：69.8 C：70.0 | T:30 C:30 | T:SF+CT  C:CT | 14d | ①②⑰ |
| **22** | GUO SC 2013[22] | RCT | T：65.8±1.3 C：65.7±1.4 | T:44 C:44 | T:HQ+CT  C:CT | 4w | ② |
| **23** | HE YP 2001[23] | RCT | T：68 C：64 | T:34 C:33 | T:MLN+CT  C:CT | 14d | ① |
| **24** | HE ZC 2010[24] | RCT | T：61.3±6.7 C：62.7±7.3 | T:30 C:30 | T:HQ+CT  C:CT | 14d | ①②⑮ |
| **25** | HE JX 1998[25] | RCT | T：61.3±6.7 C：62.7±7.3 | T:36 C:34 | T:CWJ+CT  C:CT | 14d | ① |
| **26** | HU LJ 2021[26] | RCT | T：53.18±2.92 C：52.34±3.12 | T:50 C:50 | T:SM+CT  C:CT | 14d | ① |
| **27** | HU M 2018[27] | RCT | T：60.11±10.24 C：61.24±10.43 | T:57 C:57 | T:SM+CT  C:CT | 14d | ①②⑰ |
| **28** | HU RX 2008[28] | RCT | - | T:23 C:23 | T:SHM+CT  C:CT | 14d | ① |
| **29** | HUANG AH 1999[29] | RCT | - | T:36 C:30 | T:CWJ+CT  C:CT | 14-21d | ① |
| **30** | HUANG X 2009[30] | RCT | - | T:48 C:38 | T:SQFZ+CT  C:CT | 14d | ①② |
| **31** | HUANG YB 2015[31] | RCT | T：64.27±5.82 C：66.12±5.46 | T:30 C:30 | T:SF+CT  C:CT | 7d | ②⑬ |
| **32** | HUANG ZG 2014[32] | RCT | T：56.7±13.8 C：56.5±14.5 | T:52 C:54 | T:SF+CT  C:CT | 14d | ①②⑫ |
| **33** | JIANG KJ 2004[33] | RCT | T：63.0±6.7 C：65.7±7.39 | T:52 C:54 | T:CWJ+CT  C:CT | 14d | ①② |
| **34** | LI HZ 2019[34] | RCT | T：62.5±5.3 C：61.8±4.8 | T:32 C:32 | T:MLN+CT  C:CT | 15d | ① |
| **35** | LI XL 2022[35] | RCT | T：61.24±4.57 C：61.32±4.61 | T:35 C:35 | T:SM+CT  C:CT | 4W | ②⑪⑫⑭⑮ |
| **36** | LI XP 2019[36] | RCT | T：64.4±7.1 C：65.3±6.5 | T:40 C:40 | T:SM+CT  C:CT | 14d | ①②③⑮⑰ |
| **37** | LI YY 2017[37] | RCT | T：53.68±10.14 C：54.02±10.17 | T:28 C:28 | T:SHM+CT  C:CT | 14d | ②⑨⑩⑫ |
| **38** | LIANG XL 2018[38] | RCT | T：58.80±14.87 C：56.90±15.24 | T:40 C:40 | T:SM+CT  C:CT | 21d | ①②⑤⑥⑦⑧ |
| **39** | LIN YQ 2021[39] | RCT | T：64.42±3.17 C：64.23±3.14 | T:46 C: 46 | T:SHM+CT  C:CT | 14d | ②③⑪⑫ |
| **40** | LIU D 2017[40] | RCT | T：60.34±2.31 C：61.13±2.25 | T:60 C:60 | T:SF+CT  C:CT | 14d | ①②④⑬⑭⑮⑰ |
| **41** | LIU H 2016[41] | RCT | T：65.11±9.74 C：66.33±5.09 | T:60 C:60 | T:SM+CT  C:CT | 10d | ②⑭ |
| **42** | LIU JQ 2013[42] | RCT | T：58.3±2.3 C：57.9±2.5 | T:22 C:22 | T:SM+CT  C:CT | 7d | ① |
| **43** | LIU R 2020[43] | RCT | - | T:37 C:37 | T:HQ+CT  C:CT | 14d | ①⑮ |
| **44** | LIU ZH 2024[44] | RCT | T：71.71±4.29 C：72.41±3.72 | T:60 C: 60 | T:MLN+CT  C:CT | 10d | ①② |
| **45** | MO YF 2016[45] | RCT | T：62.8±4.6  C：62.5±4.9 | T:41 C:41 | T:SF+CT  C:CT | 14d | ①② |
| **46** | MU J 2017[46] | RCT | T： 58.2±4.3  C： 56.1±6.2 | T:42 C:42 | T:SF+CT  C:CT | 14d | ①②⑨⑫⑰ |
| **47** | PENG JS 2017[47] | RCT | T：66.4±3.5 C： 65.8±3.0 | T:48 C: 48 | T:SF+CT  C:CT | 14d | ①②⑩⑰ |
| **48** | SUN XX 2020[48] | RCT | T：65.7±6.2 C：66.1±6.1 | T:37 C: 37 | T:SM+CT  C:CT | 14d | ①②⑬⑭⑮⑰ |
| **49** | TANG WH 2020[49] | RCT | T：66.45±8.28 C：67.38±8.37 | T:47 C:47 | T:MLN+CT  C:CT | 14d | ①②⑪⑫⑯ |
| **50** | WANG JX 2014[50] | RCT | T：62.5 ± 8.2 C：59.6 ±8.5 | T:28 C:28 | T:SM+CT  C:CT | 14d | ①②③⑬ |
| **51** | WEN J 2009[51] | RCT | - | T:28 C: 28 | T:SM+CT  C:CT | 14d | ② |
| **52** | WU BR 2009[52] | RCT | T：62.2 C：65.5 | T:52 C:46 | T:SM+CT  C:CT | 15d | ① |
| **53** | WU HK 2018[53] | RCT | T：64.3±9.4  C：65.8±9.5 | T:60 C:60 | T:SM+CT  C:CT | 14d | ②④ |
| **54** | WU JL 2023[54] | RCT | T：63.15±5.88  C：62.74±6.05 | T:32 C:32 | T:SM+CT  C:CT | 14d | ①② |
| **55** | WU J 2022[55] | RCT | T：67.91±11.99  C：64.71±9.84 | T:36 C:36 | T:SM+CT  C:CT | 10d | ②⑤⑥⑦⑧⑯⑰ |
| **56** | WU PH 2015[56] | RCT | T：63.5±9.94 C：67.26±9.85 | T:30 C: 30 | T:SHM+CT  C:CT | 14d | ② |
| **57** | XIAO Y 2005[57] | RCT | - | T:30 C: 30 | T:SHM+CT  C:CT | 14d | ② |
| **58** | LU R 2013[58] | RCT | T：57.5±8.2  C：58.4±7.8 | T:32 C: 32 | T:SM+CT  C:CT | 14d | ①②④⑭⑮ |
| **59** | YIN WW 2017[59] | RCT | T：59.26±5.91 C：58.65±5.69 | T:33 C:32 | T:MLN+CT  C:CT | 15d | ①② |
| **60** | YU HT 2020[60] | RCT | T： 71.96±6.72  C：72.63±6.86 | T:62 C:68 | T:SF+CT  C:CT | 14d | ②③ |
| **61** | LIU YP 2020[61] | RCT | T：63.05±5.1 C：62.54±5.2 | T:59 C:59 | T:SM+CT  C:CT | 28d | ①②④⑭⑮⑰ |
| **62** | YUAN Z 2017[62] | RCT | T：66.36±2.57 C：66.38±2.59 | T:71 C:71 | T:MLN+CT  C:CT | 21d | ①②⑪⑫ |
| **63** | ZHANG BG 2015[63] | RCT | T：64.27±5.82  C：66.12±5.46 | T:30 C:30 | T:SF+CT  C:CT | 14d | ①② |
| **64** | ZHANG FF 2018[64] | RCT | T：61.3±4.6  C：62.1±5.3 | T:70 C:70 | T:SM+CT  C:CT | 14d | ①②③⑫⑮⑯⑰ |
| **65** | ZHANG LH 2013[65] | RCT | - | T:45 C:44 | T:SF+CT  C:CT | 14d | ②⑰ |
| **66** | ZHANG L 2023[66] | RCT | T：52.42±9.56 C：52.39±9.91 | T:49 C:49 | T:CWJ+CT  C:CT | 14d | ①②③⑭⑰ |
| **67** | ZHANG WG 2016[67] | RCT | T：67.5 C：65.0 | T:84 C:84 | T:SHM+CT  C:CT | 28d | ②③ |
| **68** | ZHANG Y 2015[68] | RCT | T：56.7±11.9  C：58.2±12.4 | T:53 C:53 | T:SF+CT  C:CT | 14d | ①②⑰ |
| **69** | ZHANG Y 2018[69] | RCT | - | T:40 C:40 | T:SF+CT  C:CT | 14d | ①② |
| **70** | ZHEN HX 2019[70] | RCT | T：68.75±3.56  C：67.88±4.51 | T:44 C:44 | T:SM+CT  C:CT | 14d | ① |

Note：T, test group; C, control group;①Clinical efficacy rates;②NIHSS;③BI;④ADL;⑤TC;⑥TG;⑦LDL-C;⑧HDL-C;⑨HCV;⑩LCV;⑪ PV;⑫ FIB;⑬ CRP;⑭ IL-6;⑮TNF-α;⑯mRS;⑰ADRs

[1] 曹春艳,黄丽娜.参麦注射液联合西药治疗急性脑梗死的疗效及不良反应分析[J].中国继续医学教育,2016,8(04):201-202.

[2] 沈安娜.清热通络法治疗动脉粥样硬化性疾病的临床及机理研究[D].广州中医药大学,2006.

[3] 沈流燕,周莉.脑苷肌肽注射液联合参附注射液对老年急性脑梗死患者神经功能恢复的影响[J].中国实用神经疾病杂志,2016,19(22):7-8.

[4] 陈红英,郭俐宏,王文杰,等.参麦注射液联合长春西汀治疗脑梗死的临床研究[J].中国医药导报,2015,12(18):126-129.

[5] 陈继斌,郑定容,王淑嫒,等.刺五加注射液对急性脑梗死患者内皮功能的改善及疗效分析[J].江西中医药,2019,50(11):45-47.

[6] 陈中明,刘文兵.参麦注射液辅助治疗对老年急性脑梗死患者红细胞免疫功能、凝血功能及细胞因子的影响[J].中国老年学杂志,2018,38(05):1043-1045.

[7] 程平荣,蒋元模,何凤麟,等.生脉注射液治疗脑梗塞急性期的临床研究[J].黑龙江中医药,2015,44(04):19-20.

[8] 程文宜,郭景瑞.参麦注射液治疗急性脑梗死40例[J].陕西中医学院学报,2011,34(04):35+39.

[9] 楚尚军.探讨参麦注射液联合rt-PA静脉溶栓对急性缺血性脑卒中患者神经功能缺损评分及日常生活能力的影响[J].首都食品与医药,2019,26(08):66.

[10] 邓庆平.益气活血法对急性脑梗死合并全身炎症反应综合征的炎症反应研究[D].广州中医药大学,2010.

[11] 邓艳.重用黄芪注射液治疗急性脑梗塞30例[J].浙江中西医结合杂志,2001,(05):28-29.

[12] 邓祝庭.波立维联合参麦注射液治疗急性脑分水岭梗死的疗效观察[J].首都医药,2011,18(10):40.

[13] 翟红珍.简述参附注射液联合西药治疗脑梗死的疗效观察[J].中国医药指南,2016,14(13):196.

[14] 翟永超.参附针治疗脑分水岭梗死疗效观察[J].中国实用神经疾病杂志,2010,13(09):64-66.

[15] 董兴富.参麦注射液联合丁苯酞治疗急性脑梗死伴糖尿病临床研究[J].新中医,2020,52(10):43-46.

[16] 杜贤兰,贾长海,秦绪,等.刺五加对急性脑梗死患者血脂、纤维蛋白原及全血粘度变化的临床意义[J].中国医药导刊,2004,(06):431-432.

[17] 冯紫薇,吴斌,韩敏,等.脉络宁注射液联合己酮可可碱治疗急性脑梗死的临床研究[J].现代药物与临床,2023,38(10):2464-2468.

[18] 伏开全,王言里.参麦注射液治疗急性脑梗死84例临床观察[J].浙江中医杂志,2010,45(07):543.

[19] 付鹏.刺五加注射液联合注射用奥扎格雷钠治疗急性脑梗死的临床疗效及对血液流变学的影响[J].临床合理用药,2024,17(33):50-53.

[20] 傅志慧,王娜,丁有钦.脉络宁注射液对缺血性中风急性期患者炎症因子的影响[J].中国中医急症,2008,(02):188-189+192.

[21] 高英梅,薛红莲.参附注射液联合依达拉奉治疗脑分水岭梗死疗效观察[J].现代中西医结合杂志,2015,24(19):2091-2093.

[22] 郭生春.黄芪注射液与血栓通注射液联合应用治疗急性脑梗死的临床分析[J].中国医药指南,2013,11(30):519-520.

[23] 何宇平,应荣斌,潘光成.低分子量肝素与脉络宁合用治疗急性脑梗死的临床疗效观察——附34例报告[J].新医学,2001,(06):335-336+339.

[24] 何正初,华力明.黄芪注射液对脑梗死急性期血清中肿瘤坏死因子α和内皮素1的影响及意义[J].临床荟萃,2010,25(03):196-199.

[25] 贺敬贤,张宏.中西医结合治疗急性脑梗塞36例疗效观察[J].中国厂矿医学,1998,(01):60-61.

[26] 胡丽娟.参麦注射液治疗急性脑梗死患者的效果及对血液流变学指标的影响[J].临床合理用药杂志,2021,14(08):89-91.

[27] 胡密.参麦注射液联合奥扎格雷对急性缺血性脑卒中患者NIHSS评分及血清Hcy、LP(a)水平变化的影响[J].医学理论与实践,2018,31(07):943-945.

[28] 胡荣鑫,张贺,王政琨.中西医结合治疗分水岭脑梗死临床分析[J].实用中医内科杂志,2008,22(12):59-60.

[29] 黄爱和,管健.刺五加注射液治疗脑卒中疗效观察[J].现代中西医结合杂志,1999,(06):913.

[30] 黄霞.参芪扶正注射液治疗急性脑梗死的临床效果[J].海南医学,2009,20(04):88-89.

[31] 黄郁斌,张保国,温淑端,等.参附注射液对脑梗死急性期血浆D-二聚体及CRP的影响[J].实用中医药杂志,2015,31(06):547-548.

[32] 黄志庚.参附注射液联合西药治疗脑梗塞疗效观察[J].陕西中医,2014,35(06):665-666.

[33] 姜克家,茆华武,潘怀富.西医常规疗法加刺五加注射液治疗急性脑梗死的临床观察[J].中国中西医结合急救杂志,2003,(06):384.

[34] 李洪州,王奎,任洪军.脉络宁辅助治疗急性脑梗死的效果研究[J].当代医药论丛,2019,17(23):113-114.

[35] 李小丽,戴中慈.参麦注射液治疗急性缺血性脑卒中患者的临床研究[J].现代医学与健康研究电子杂志,2022,6(10):24-27.

[36] 李晓培.参麦注射液联合丁苯酞氯化钠注射液治疗急性脑梗死的疗效及对血清炎性指标的影响[J].中国疗养医学,2019,28(06):623-626.

[37] 李贻勇.生脉注射液治疗脑梗塞的临床分析[J].实用妇科内分泌杂志(电子版),2017,4(34):115-116.

[38] 梁小龙,王文华,石庆新,等.参麦注射液联用瑞舒伐他汀钙治疗急性缺血性脑卒中临床观察[J].新中医,2018,50(11):62-66.

[39] 林涌泉,李薛莹,陈明凤.生脉注射液辅助治疗对低灌注/栓子清除能力下降型脑梗死患者脑血流动力学的影响[J].基层医学论坛,2021,25(16):2240-2243.

[40] 刘冬,张盛,唐平易.参附注射液与依达拉奉注射液联合治疗急性脑梗死的疗效观察[J].中国医院用药评价与分析,2017,17(10):1360-1362.

[41] 刘辉,刘美香,马丽丽.参麦注射液对脑梗死患者血白细胞介素6的影响[J].中国实用神经疾病杂志,2016,19(12):124.

[42] 刘建青.急性脑梗死分水岭类型临床治疗效果分析[J].当代医学,2013,19(12):79-80.

[43] 刘润,王小智,于航.黄芪注射液联合亚低温对急性脑梗死溶栓术后缺血再灌注患者脑保护作用[J].中国临床研究,2020,33(03):299-302.

[44] 刘子辉,刘会,徐清华,等.脉络宁注射液联合西药对脑梗死患者血管再生及大脑动脉血流速度的影响[J].中国中医急症,2024,33(10):1819-1821.

[45] 莫云芳. 参附注射液治疗脑梗死急性期(元气虚衰证)的临床研究[J]. 医药前沿,2016,6(5):365-366.

[46] 穆婧,刘承玄,李云逸.参附注射液联合奥扎格雷钠注射液治疗进展性脑梗死临床观察[J].中国药业,2017,26(08):52-54.

[47] 彭景拾. 脑苷肌肽注射液联合参附注射液治疗老年急性脑梗死效果及安全性[J]. 国际医药卫生导报,2017,23(23):3742-3744.

[48] 孙小雪.参麦注射液联合丁苯酞注射液治疗急性分水岭脑梗死患者的效果观察[J].中国现代药物应用,2020,14(11):114-115.

[49] 唐文华,姜秀云. 脉络宁注射液联合阿替普酶对急性缺血性脑卒中患者血液流变学的影响[J]. 国际医药卫生导报,2020,26(16):2392-2394.

[50] 王家祥.参麦注射液对急性缺血性脑卒中患者血清C反应蛋白的影响[J].河南中医,2014,34(04):638-639.

[51] 文洁.中西医结合治疗脑分水岭梗塞56例疗效观察[J].海南医学,2009,20(09):42-43.

[52] 吴宝荣,张小平.参麦注射液治疗低血压性脑梗死52例临床疗效观察[J].现代医药卫生,2009,25(24):3712-3713.

[53] 吴海科,梁艳桂,陈杰,等.参麦注射液对分水岭脑梗死患者气虚、阴虚证候评分及肢体运动功能的影响[J].中国中西医结合杂志,2018,38(08):950-953.

[54] 吴佳琳,李月红.依达拉奉联合参麦注射液治疗脑梗死合并冠心病患者的疗效及对血小板活化、认知功能的影响[J].心血管病防治知识,2023,13(13):6-8+12.

[55] 吴婧.基于apelin通路探讨益气扶正法对静脉溶栓后脑梗死患者的疗效及机制[D].北京中医药大学,2022.

[56] 吴佩华,王爱梅,郑秀利.生脉注射液对低灌注/栓子清除能力下降型脑梗死脑血流动力学的影响[J].山西中医学院学报,2015,16(01):46-47.

[57]肖雁,唐冬良,江拥军.生脉注射液治疗急性脑梗死的疗效观察[J].人民军医,2005,(09):501-502.

[58] 鲁锐,杨平.参麦注射液对急性缺血性脑卒中患者血清TNF-α和IL-6的影响[J].中西医结合心脑血管病杂志,2013,11(11):1399-1400.

[59] 尹文伟,陈思,裴璐,等.脉络宁注射液辅助治疗急性脑梗死的临床观察[J].中国药房,2017,28(20):2834-2837.

[60] 于海涛,刘秋皖,吴君仓.参附注射液治疗非心源性脑卒中疗效观察及对超敏C反应蛋白、胱抑素C的影响[J].安徽医药,2020,24(05):989-992.

[61] 刘云平,赵洪海,张贺齐,等.参麦注射液治疗急性缺血性脑卒中早期神经功能恶化的疗效及对血清细胞因子水平的影响[J].中国医院用药评价与分析,2020,20(03):286-289.

[62] 袁州.脉络宁注射液联合脑苷肌肽治疗急性缺血性脑血管病的临床研究[J].现代药物与临床,2017,32(12):2363-2367.

[63] 张保国,黄郁斌,杨小兰.参附注射液治疗脑梗死急性期(元气虚衰证)的临床观察[J].中国中医急症,2015,24(08):1438-1440.

[64] 张飞飞,孙妍,张丽,等. 参麦注射液联合丁苯酞治疗急性脑梗死的临床研究[J]. 现代药物与临床,2018,33(7):1635-1639.

[65] 张良红.参附注射液联合克林澳治疗急性脑梗死临床分析[J].吉林医学,2013,34(12):2263-2264.

[66] 张亮,刘万周,赵菁,等.刺五加注射液联合阿加曲班治疗急性脑梗死的临床研究[J].现代药物与临床,2023,38(12):2990-2995.

[67] 张卫国,王素洁,刘海燕.生脉注射液对轻中度脑梗死超时间窗rt-PA溶栓患者预后的影响[J].中国药房,2016,27(21):2918-2920.

[68] 张颖.克林澳联合参附注射液治疗急性脑梗死疗效评价[J].中国实用神经疾病杂志,2015,18(23):114-115.

[69] 张宇,郭楠,孔令博,等.参附注射液在治疗内分水岭脑梗死患者的临床疗效[J].世界中医药,2018,13(09):2166-2170.

[70] 甄海旭. 参麦注射液联合阿替普酶静脉溶栓治疗急性缺血性脑卒中患者的疗效分析[J]. 糖尿病天地,2019,16(11):30.
